# Biological significance of *MYC* and *CEBPD* coamplification in urothelial carcinoma: Multilayered genomic, transcriptional and posttranscriptional positive feedback loops enhance oncogenic glycolysis

**DOI:** 10.1002/ctm2.674

**Published:** 2021-12-26

**Authors:** Ti‐Chun Chan, Yi‐Ting Chen, Kien Thiam Tan, Chia‐Ling Wu, Wen‐Jeng Wu, Wei‐Ming Li, Ju‐Ming Wang, Yow‐Ling Shiue, Chien‐Feng Li

**Affiliations:** ^1^ Department of Medical Research Chi Mei Medical Center Tainan Taiwan; ^2^ National Institute of Cancer Research National Health Research Institutes Tainan Taiwan; ^3^ Department of Biotechnology and Bioindustry Sciences College of Bioscience and Biotechnology National Cheng Kung University Tainan Taiwan; ^4^ ACT Genomics Co., Ltd. Taipei Taiwan; ^5^ Graduate Institute of Clinical Medicine College of Medicine Kaohsiung Medical University Kaohsiung Taiwan; ^6^ Department of Urology Kaohsiung Medical University Hospital Kaohsiung Taiwan; ^7^ Department of Urology School of Medicine College of Medicine Kaohsiung Medical University Kaohsiung Taiwan; ^8^ Department of Urology Ministry of Health and Welfare Pingtung Hospital Pingtung Taiwan; ^9^ Institute of Precision Medicine National Sun Yat‐Sen University Kaohsiung Taiwan; ^10^ Department of Pathology School of Medicine College of Medicine Kaohsiung Medical University Kaohsiung Taiwan

**Keywords:** CEBPD, coamplification, glycolysis, MYC, urothelial carcinoma

## Abstract

**Background and purpose:**

The aim of this study is to decipher the underlying mechanisms of CCAAT/enhancer‐binding protein delta (CEBPD)‐enhanced glycolysis as well as the biological significance of *CEBPD* and *MYC* coamplification in urothelial carcinoma (UC).

**Methods:**

In vitro analyses were conducted to examine the effects of altered CEBPD or MYC expression on UC cells. The in vivo effects of CEBPD overexpression in a high‐glucose environment on tumour growth were investigated in xenografted induced diabetic severe combined immunodeficiency/beige mice. Data mining was used to cross‐validate the associations between *CEBPD* and *MYC* copy number and transcriptional expression, quantitative reverse transcription‐polymerase chain reaction, immunohistochemistry, chromogenic in situ hybridization, and in situ hybridization targeting microRNA were performed on 635 UC patient samples and xenograft samples. UC patient survival in relation to diabetes was validated by using the National Health Insurance Research Database.

**Results:**

*CEBPD* and *MYC* coamplification (29.6%) occurred at a high frequency, MYC expression promoted chromosomal instability, facilitating *CEBPD* copy number gain and expression. CEBPD promoted glucose uptake and lactate production by upregulating SLC2A1 and HK2, leading to mitochondrial fission, increased extracellular acidification rate and decreased oxygen consumption rate to fuel cell growth. CEBPD upregulated HK2 expression through multiple regulation pathways including MYC stabilization, suppression of *FBXW7* transactivation and MYC‐independent transcriptional suppression of hsa‐miR‐429. Clinical and xenografted experiments confirmed the growth advantage of CEBPD in relation to glucose metabolic dysregulation and the significant correlations between the expression of these genes.

**Conclusions:**

We confirmed that CEBPD has an oncogenic role in UC by activating AKT signalling and initiating metabolic reprogramming from mitochondrial oxidative phosphorylation to glycolysis to satisfy glucose addiction. These novel CEBPD‐ and MYC‐centric multilayered positive feedback loops enhance cancer growth that could complement theranostic approaches.

## Graphical abstract

• *MYC* and *CEBPD* coamplification predicts poor outcomes in urothelial carcinoma.

• MYC enhances amplification‐driven CEBPD expression and CEBPD stabilize MYC protein through transcriptional suppression of *FBXW7*, forming a positive feedback loop.

• CEBPD facilitates the switch from mitochondrial oxidative phosphorylation to glycolysis in both MYC‐dependent and a Myc‐independent CEBPD ‐ hsa‐miR‐429 ‐ HK2 regulatory axis that confers pro‐proliferative effect aggravated by high glucose condition.

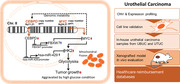

## 1 INTRODUCTION

Urothelial carcinoma (UC), also known as transitional cell carcinoma, is a highly prevalent malignancy worldwide. Urinary bladder UC (UBUC) accounts for the majority (90%–95%) of UCs, while the remaining 5%–10% of UCs are upper urinary tract urothelial cancer (UTUC).^1^ UTUC exhibits an unusual prevalence in some countries, such as Taiwan.^2^ Combining standard treatment with molecularly matched targeted therapy based on biomarkers is emerging as a viable strategy to improve cancer management. However, the high genetic and histological heterogeneity of UC presents a challenge in terms of its diagnosis, conventional therapy response, and prognostication.

Environmental toxins, infections and genetic aberrations impact the progression of UC.^3^, ^4^, ^5^ Genomic instability (CIN) is a cancer hallmark featured by an increased tendency of genome alteration during cell division owing to the defects in DNA damage checkpoint, DNA repair machinery as well as a mitotic checkpoint.^6^ Of these, CIN is suggested as a consequence of MYC‐induced DNA replication aberration and cell division dysregulation.^7^ Numerical CIN, which is related to the gain/loss of whole chromosomes, or structural CIN, which is involved in the amplification/deletion/inversion/translocation of chromosomal regions, have been shown to have a profound impact on the carcinogenesis of UC and have become prospective therapeutic targets.^8^, ^9^

Recently, we showed that the CIN involving 8q11.21 is linked to poor outcomes in our array comparative genomic hybridization (aCGH) UBUC cohort (*n* = 40). Within that CIN region, amplification of CCAAT/enhancer‐binding protein delta (*CEBPD*) was linked to an adverse prognosis due to rapid tumour proliferation and metastasis.^10^ *CEBPD* encodes a transcription factor responsible for physiological processes, including cell differentiation, proliferation, metabolism, inflammation, growth arrest, and cell death.^11^ Of note, CEBPD has dual functions as a tumour suppressor in breast cancer and pancreas cancer^12^, ^13^ but an oncogene in glioblastoma and UC^10^, ^14^ depending on the environmental stimulus and cancer type.^15^ Surprisingly, in the present study, we reanalysed our previously published UBUC aCGH dataset and TCGA dataset with special attention to the chromosome 8q which is the most frequently gained region. *CEBPD* amplification is frequently accompanied by MYC protooncogene (*MYC*) amplification (both *p *< .01). Oncogenic *MYC* is located on chromosome 8q24.21, which is 80 Mb away from *CEBPD*, and contributes to tumour progression via metabolic reprogramming (i.e., aerobic glycolysis).^16^, ^17^ Dysregulation of MYC has also been shown to be involved in genome instability,^18^ where *MYC* amplification has been reported to be closely linked to *ERBB2* proto‐oncogene (mapped to 17q12) amplification to strongly activate cell proliferation in breast cancer.^19^ In addition, MYC overexpression drives dihydrofolate reductase (*DHFR*, mapped to 5q14.1) amplification,^20^ which increases metastatic risk.^21^ Because *MYC* amplification occurs earlier and more frequently than *CEBPD* amplification in our cohort, we hypothesized that *CEBPD* amplification might be closely associated with and at least partly driven by *MYC* and that both collaborate in the metabolic dysregulation of UC, which has not been mentioned previously. Accordingly, the current work aimed to determine the effect of CEBPD on tumour development and clinicopathological features and to decipher the positive feedback loops driven by the crosstalk between CEBPD and MYC at the genomic, transcriptional and posttranscriptional levels in vitro through quantitative polymerase chain reaction/reverse transcription‐polymerase chain reaction (PCR/RT‐PCR), western blot, glucose and lactate quantification, cell viability assay, Mito‐Red mitochondrial image analysis, Seahorse assays for extracellular acidification rate (ECAR) and oxygen consumption rate (OCR) detection, luciferase reporter assays, and RNA‐seq targeting small RNA in two distinct UC cells. In addition, in vivo study via data mining, quantitative RT‐PCR, immunohistochemistry (IHC), chromogenic in situ hybridization (CISH), and in situ hybridization targeting microRNA in 635 UC patients and xenografted severe combined immunodeficiency (SCID)/beige mice was as conducted. The correlation between UC patient survival and diabetes was validated in a population‐based manner in National Health Insurance Research Database (NHIRD).

## 2 MATERIALS AND METHODS

### 2.1 Tumour tissue sample sets

In this study, we performed IHC and CISH of well‐established sample sets consisting of 295 UBUC and 340 UTUC samples from the biobank of Chi Mei Medical Center. The archived tumour samples were collected after surgery with curative intent between January 1996 and May 2004 as previously described.^22^ A total of 32 snap‐frozen UC samples with paired non‐tumour urothelium were evaluated for *CEBPD* mRNA and protein expression. This study was approved by the institutional review board of Chi Mei Medical Center (IRB10207‐001).

### 2.2 Reanalysis of aCGH data

Reanalysis of our published aCGH dataset containing 40 UBUC samples^10^ was performed by using Nexus Copy Number software (BioDiscovery, USA) as previously described^23^ to profile the status of *CEBPD* and *MYC* coamplification. For this analysis, the copy‐number gain was defined as log2 ratio >0.2.

### 2.3 Data mining

We deciphered the correlation between the *CEBPD* and *MYC* gene dosages in The Cancer Genome Atlas (TCGA)‐bladder cancer (BLCA) dataset and the association between the *CEBPD* and *HK2* gene expression in the GSE13507 dataset through the Oncomine platform (research premium edition).

### 2.4 Cell culture

Four human UC‐derived cell lines including RT4 (American Type Tissue Culture Collection [ATCC], USA), HT1197 (ATCC, USA), TCCSUP (ATCC, USA) and BFTC909 (Food Industry Research and Development Institute, Taiwan) were utilized in this paper. RT4 was incubated in McCoy's 5A Medium with 10% fetal bovine serum (FBS) and 1% penicillin/streptomycin (P/S). HT1197 was maintained in minimum essential media (MEM) with 10% FBS, 1% non‐essential amino acid and 1X antibiotic‐antimycotic (100X). BFTC909 and TCCSUP were cultured in Dulbecco's modified eagle medium (DMEM) supplemented with 10% FBS and 1% P/S. All cells were incubated in a humidified incubator containing 5% CO_2_ at 37°C. The cell culture media, fetal bovine serum and antibiotics were all purchased from Gibco.

### 2.5 Exogenous gene overexpression in cell lines

Phoenix‐AMPHO cells (ATCC, USA) were cotransfected with lentiviral vector contained gene of interest, lentiviral packaging plasmids (psPAX2) and lentiviral envelope plasmid (pMD2.G) dissolved in the Opti‐MEM I reduced‐serum medium (Gibco) through PolyJet transfection reagents (SignaGen Laboratories). The viral supernatant was harvested 48 h post‐transfection. Cell lines were infected with viral particles carrying the target gene and became stable clones through the selection with puromycin (2 μg/ml). Detailed information is shown in Supporting Information.

### 2.6 Plasmids and oligomers

pLKO‐AS3w‐eGFP, pLKO‐AS3w‐*CEBPD*‐eGFP^10^ and Lenti‐*MYC*‐Myc‐DDK (RC201611L1, Origene) were used for the production of viral particles carrying indicated target genes. pLV‐mitoDsRed vector (44386, Addgene, USA) embracing a mitochondrial targeting sequence fused to a recombinant red fluorescent protein (dsRed) was used to detect mitochondrial fission and fusion events. SignalSilence *c‐Myc* small interfering RNAs (siRNAs) (6341, 6552; Cell Signaling Technology, USA) were used for the knockdown of MYC expression. Analysis of promoter activity was conducted by using the following reporters: F‐box and WD repeat domain‐containing 7 (*FBXW7*) promoter (S700873; Active Motif, USA), hsa‐miR‐429‐wild‐type (WT) promoter‐reporter (HPRM 22169‐PG04; Genecopoeia), hsa‐miR‐429‐mutant promoter‐reporter (TopGen, Taiwan), hsa‐miR‐429 mimic (TopGen), hsa‐miR‐429 inhibitor (TopGen), pKM2L‐ph*HKII* (RDB05882; Riken, Japan), pGL4.54[luc2/TK] vector served as a control vector (E5061; Promega, USA), WT‐*HK2*‐3′‐untranslated region (UTR) and mutant‐*HK2*‐3′‐UTR cloned into the pMIR‐REPORT Luciferase MicroRNA (miRNA) Expression Reporter Vector contains firefly luciferase (TopGen).

### 2.7 miRNA sequencing

Note that, 1 μg of total RNA extracted by TRI Reagent RNA Isolation Reagent (Sigma, USA) was used to construct a small RNA library with a TruSeq Small RNA Sample Preparation Kit (Illumina, US) according to its manual instructions. The libraries were applied for sequencing with an Illumina NextSeq sequencer (Illumina, CA, USA). After that, the FASTX‐Toolkit (http://hannonlab.cshl.edu/fastx_toolkit) was used for the quality control process. The obtained information was profiled by using miRBase v21 following alignment. An expectation‐maximization algorithm was used to normalize miRNA expression data. Finally, the expression level of each miRNA was divided by the total number of aligned reads for precise quantification. The downregulated miRNAs in the CEBPD‐overexpressing group with a fold change no less than log2 ratio ‐1 in at least one cell and log2 ratio exceeding ‐0.3 in another cell with a significance of *p *< .05 in duplication tests were selected for further analysis to ensure the significance of the miRNA candidates. The entire protocol was presented in the supplementary materials and methods.

### 2.8 Real‐time quantitative RT‐PCR to quantify the mRNA and hsa‐miRNA levels

Total RNA extraction from cell lines was utilized by Quick‐RNA Miniprep Kit (Zymo Research, CA, USA) according to the manufacturer's instructions. For the evaluation of mRNA expression, 1 μg of total mRNA was subjected to cDNA synthesis using the Maxima First Strand cDNA Synthesis Kit (Thermo Scientific) with the following thermal cycling steps: 25°C for 10 min, 50°C for 30 min and a final incubation at 85°C for 5 min. The mixture containing cDNA, predesigned TaqMan assay reagent (probe and primer set: *CEBPD* [Hs00270931_m1], *MYC* [Hs00153408_m1], *FBXW7* [Hs00217794_m1] and *HK2* [Hs01034055_g1]; Applied Biosystems) and TaqMan Fast Advanced Master Mix (Applied Biosystems) were subjected to quantitative RT‐PCR to determine the mRNA level via a StepOne Plus System (Applied Biosystems) at the indicated thermal cycling: 20 s at 95℃, followed by 40 cycles of 95℃ for 1 s and 60℃ for 20 s. The cycle threshold (Ct) value of the target gene was normalized to that of the reference gene *POLR2A*, which served as the ∆Ct value. The relative mRNA expression of the target gene to the control gene was estimated using equation 2^–∆∆CT^. For the determination of miRNA expression, a TaqMan Advanced miRNA cDNA Synthesis Kit (Thermo Scientific) was utilized to prepare the cDNA template of miRNA. Afterwards, the mixture of cDNA, predesigned TaqMan assay mixture (probe and primer set targeting hsa‐miR‐429, U6 snRNA; TopGen) and distilled water were subjected to quantitative RT‐PCR in the following thermal cycling conditions: 20 s at 95°C, followed by 40 cycles of 95°C for 1 s and 60°C for 20 s. The relative miRNA level was calculated by the 2^–∆∆CT^ formula and U6 snRNA was used as a reference gene. The detailed information was indicated in the supplementary materials and methods.

### 2.9 Cell viability and cell proliferation assays

2,3‐Bis‐(2‐methoxy‐4‐nitro‐5‐sulfophenyl) 2H‐tetrazolium‐5‐carboxanilide (XTT) was used to evaluate cell viability. Cells incubated in a 96‐well microplate at indicated hours were treated with a mixture of XTT and N‐methyl dibenzopyrazine methyl sulfate. Afterwards, the absorbance was measured at a 450 nm wavelength with a reference wavelength at 600 nm in an ELISA reader (GM3000; Promega). Cell proliferation was evaluated by a Cell Proliferation Assay Kit (Fluorometric; Biovision). The fluorescence value proportional to cell number was evaluated at excitation/emission wavelengths of 480/538 nm. The complete procedure is described as Supporting Information.

### 2.10 Western blot assays

Total protein was isolated using PRO‐PREP Protein Extraction Solution (iNtRON). Thirty micrograms of protein were separated on a NuPAGE Bis‐Tris Gel (Invitrogen) and transferred onto an Immobilon‐P polyvinylidene difluoride membrane (Millipore). After blocking with skim milk (Sigma), membranes were incubated with indicated primary and appropriate secondary antibodies. Enhanced chemiluminescence (Thermo Scientific) is used for visualizing the proteins of interest.

Primary and secondary antibodies are listed below. Primary antibodies : phospho‐MAPK3/1 (Erk1 [pT202/pY204] + Erk2 [pT185/pY187], Abcam, ab50011), MAPK3/1 (Erk1/2) (Cell Signaling, 4695S), phospho‐PI3K (Tyr 607, Abcam, ab182651), PI3K (Cell Signaling, 4292S), phospho‐AKT (Ser473, Cell Signaling, 4060), AKT (Cell Signaling, 4691), phospho‐MTOR (Ser2448, Abcam, ab51044,), MTOR (Abcam, ab32028), phospho‐RPS6 (Ser235, Abcam, ab80158), RPS6 (Abcam, ab137826,), phospho‐4E‐BP1 (Ser65, Cell Signaling, 9451), 4E‐BP1 (Cell Signaling, 9644), caspase‐3 (active) (EPITOMICS, 1476‐1), FBXW7 (Abcam, ab105752), SKP2 (Abcam, ab124799), MYC (Abcam, ab32072), SLC2A1 (Cell Signaling, ab105752), HK2 (Cell Signaling, 2867), and GAPDH (Abcam, ab181602). Secondary antibody: HRP Donkey anti‐rabbit IgG (BioLegend, 406401), HRP goat anti‐mouse IgG H&L (Abcam, ab97023). The detailed protocol is exhibited in the Supporting Information.

### 2.11 Flow cytometric analysis

For cell cycle analysis, harvested cells proceeded to fix by 70% of ethanol at –20°C overnight. After washing with PBS, cells were resuspended with propidium iodide (PI)/ribonuclease staining buffer (550825; BD Biosciences, USA) protected from light for 15 min at room temperature. The cell cycle distribution was analysed by using a Novocyte flow cytometer (ACEA Biosciences, USA). For apoptosis assay, live cells were stained with 5 μl of PI and 5 μl of APC Annexin V (51‐66211E, 559925; BD Biosciences) followed by the incubation for 5 min in the dark. The number of apoptotic cells was evaluated by a Novocyte flow cytometer.

### 2.12 Glucose uptake assays

Glucose uptake was evaluated by a Glucose Uptake Assay Kit (Colorimetric, Abcam). Briefly, glucose transporters of glucose‐starved cells were activated by insulin. Note that, 2‐deoxyglucose (2‐DG), an analogue of glucose, was taken up by glucose transporters and was metabolized to 2‐DG‐6‐phosphate (2‐DG6P). Accumulated 2‐DG6P in cells is proportional to the amount of glucose uptake by glucose transporters. Note that, 2‐DG6P was oxidized to produce NADPH and underwent an enzymatic recycling amplification reaction to generate a coloured product. The absorbances were measured at a wavelength of 405 nm with an ELISA reader (GM3000, Promega). The complete process was presented in the supplementary materials and methods.

### 2.13 Lactate level analysis

Lactate production was assessed by using an L‐lactate assay kit (Colorimetric, ab65331; Abcam).

Briefly, the intracellular lactate was extracted from the homogenized cells lysed by the lactate assay buffer and was catalysed by lactate dehydrogenase to produce a material that reacted with a substrate to generate a coloured product. The absorbances were evaluated at a wavelength of 450 nm with an ELISA reader (GM3000; Promega). The detailed procedure is shown in the Supporting Information.

### 2.14 ECAR and OCR assays

The ECAR and OCR were assessed by an Agilent Seahorse XFp Analyzer (Seahorse Bioscience; USA) according to its manufacturer's instructions. Briefly, for ECAR assay, cells were seeded in an 8‐well cell culture miniplate for 24 h and underwent glucose starvation for 30 min.

Three compounds including glucose, oligomycin, 2‐DG were sequentially loaded into the 8‐well cell culture miniplate with glucose‐starved cells at indicated time point to evaluate the maximal glycolytic capacity based on the value of ECAR. For the OCR test, three reagents containing oligomycin, carbonyl cyanide‐p‐trifluoromethoxyphenylhydrazone and rotenone/antimycin A were orderly injected into cells seeded into an 8‐well miniplate to directly evaluate the OCR of cells. The complete method is indicated as Supporting Information.

### 2.15 Luciferase reporter assays

The *FBXW7* promoter‐reporter (optimized Renilla luciferase) or pGL4.54[luc2/TK] vector (firefly luciferase) was transfected into cells by using PolyJet In Vitro DNA Transfection Reagent according to its protocol. The activity of optimized Renilla luciferase and firefly luciferase were assessed by LightSwitch Luciferase Assay Kit (Active Motive) and ONE‐Glo Luciferase Assay System (Promega) according to manufacturer's instruction (Supporting Information), respectively.

The Renilla luminescence value was normalized by the firefly luminescence value and represented the activity of *the FBXW7* promoter.

The hsa‐miR‐429 promoter activity was conducted under a dual‐reporter system comprising an hsa‐miR‐429 promoter with gaussian luciferase (GLuc) reporter and a secreted alkaline phosphatase (SEAP) reporter served as an internal control. After transfection of the hsa‐miR‐429 promoter dual‐reporter in cells, the activity of secreted GLuc and SEAP was detected by Secrete‐Pair Dual Luminescence Assay (Genecopoeia) in line with the manufacturer's instructions.

Firefly luciferase reporter vector containing WT‐*HK2*‐3′‐untranslated region (UTR) or mutant‐*HK2*‐3′‐UTR was cotransfected with pGL4.74[hRluc/TK] into cells. The luminescence signal was measured by a Dual‐Glo Luciferase Assay System (Promega) according to the manufacturer's instructions. The firefly luminescence units were normalized by the Renilla luminescence units to reflect the activity of each *HK2*‐3′‐UTR reporter.

Mock‐ and CEBPD‐overexpressing cells were cotransfected with pKM2L‐ph*HKII* (Renilla luciferase) and pGL4.54[luc2/TK] vectors, followed by the treatment of hsa‐miR‐429 mimic or control mimic. The luminescence signal was measured by a Dual‐Glo Luciferase Assay System (Promega). Finally, the Renilla luminescence units were normalized by the firefly luminescence units to indicate *HK2* promoter activity. Complete information above is shown in Supporting Information.

### 2.16 Analysis of copy number variation by qPCR

Genomic DNA was extracted using the QIAamp DNA Mini Kit (Qiagen) according to the manufacturer's instructions. The mixture including genomic DNA, TaqMan SNP Genotyping Assay probes (PIK3R1, Hs06028467_cn; CEBPD, Hs01845884_cn; Hs01879749_cn; Hs02239340_cn) and TaqMan Genotyping Master Mix (Applied Biosystems) was subjected to quantitative RT‐PCR under StepOne Plus System (Applied Biosystems). The thermal cycling conditions were as follows: 10 min at 95 ℃, followed by 40 cycles of 95℃ for 15 s and 60℃ for 1 min. The Ct value of *CEBPD* was normalized to that of the *PIK3R1* (reference gene), and this value was defined as ∆Ct. Relative quantification (RQ) was performed by using the 2^–ΔΔCt^ formula, and the copy number was represented as 2 × RQ.

### 2.17 Next‐generation sequencing‐based loss of heterozygosity analysis

To further elucidate whether MYC expression enhances CIN, which is assumably the causal factor of *CEBPD* amplification, we use next‐generation sequencing (NGS)‐based ACTHRD panel (ACT Genomics) to measure the loss of heterozygosity (LOH) in cell lines. For this assay, genomic DNA from cells was extracted and amplified using primer pairs targeting coding exons of 24 homologous recombination repair (HRR)‐related genes (Table S1) and 8833 single nucleotide polymorphisms (SNPs) evenly distributed across the entire genome. After library preparation, the product was sequenced and analysed for mutations, copy number variations, and LOH (Supporting Information).

### 2.18 Chromatin immunoprecipitation

A SimpleChIP Enzymatic Chromatin IP Kit (Cell Signaling) was used for chromatin immunoprecipitation (ChIP) assay. Briefly, fragmented cross‐linked chromatin extracted from cells was immunoprecipitated with primary antibody against CEBPD (Santa Cruz, sc‐365546) and protein G magnetic beads at 4°C. Afterwards, the precipitated DNA was purified from the antibody/protein G complex and was subjected to quantitative RT‐PCR under the thermal cycle: 3 min at 95℃, followed by 40 cycles of 95℃ for 15 s and 60℃ for 1 min. The primers targeting CEBPD‐responsive element binding site on the hsa‐miR‐429 promoter were shown as below: forward: 5′‐GGTTCTTCCCTGGGCTTC‐3′; reverse: 5′‐AGTGTTAGAGTCAAGCTGGGAAAT‐3′.

### 2.19 High‐throughput chromosome conformation capture

To systemically explore the possibility of long‐range regulation of the MYC gene on CEBPD, we evaluate the CEBPD‐interacting genes using the high‐throughput chromosome conformation capture (Hi‐C) technique. We performed Hi‐C on both BFTC909 and TCCSUP cells using the Arima Hi‐C Kit (A510008; Arima Genomics, USA). Hi‐C experiments were performed as previously described.^24^ The library generated was submitted to a NextSeq 2000 for sequencing. Raw sequencing data were mapped and processed to the UCSC Hg19 reference genome using HiC‐Pro v3.1.0 pipeline,^25^ and restriction fragment BED file was generated by using digest_genome.py script from HiC‐Pro utilities. The R package HiTC^26^ was used to perform data normalization and to visualize the interaction map.

### 2.20 Immunohistochemistry

Formalin‐fixed, paraffin‐embedded (FFPE) tissue sections were deparaffinized and rehydrated by using xylene and ethanol, respectively. After retrieval with citrate buffer at pH 6.0, the endogenous peroxidase activity of specimens was quenched by peroxidase‐blocking solution (Dako). The sections were incubated with the primary antibody against the target protein as indicated below: MYC (Abcam, ab32072), CEBPD (Abcam, ab184911), HK2 (Cell Signaling, 2867), FBXW7 (Abcam, ab105752), phospho‐AKT (Cell Signaling, 4060), phospho‐mTOR (Abcam, ab51044), phospho‐4E‐BP1 (Cell Signaling, 9644), phospho‐RPS6 (Abcam, ab80158), and MKI67 (Abcam, ab66155), followed by the incubation of a secondary antibody (REAL EnVision/HRP, rabbit/mouse [ENV], Dako). Staining was visualized with EAL DAB+ Chromogen diluted in REAL Substrate Buffer (Dako). Hematoxylin was used for the nuclear stain. Finally, the slices were dehydrated by soaking in various concentrations of ethanol and were mounted in UltraCruz Aqueous Mounting Medium with DAPI. The IHC staining results were examined with an optical microscope and quantified into H‐scores by three expert pathologists (Chien‐Feng Li, Tzu‐Ju Chen and Wan‐Shan Li ) as previously mentioned.^22^ Detailed information is shown in Supporting Information.

### 2.21 Chromogenic in situ hybridization

The human *MYC* gene was detected by a CISH assay. Briefly, FFPE slides were deparaffinized and rehydrated by xylene and ethanol. After the treatment with 0.2 N hydrochloric acid, the slides were soaked with pretreatment buffer followed by pepsin dissolved in protease buffer. The slides were dehydrated with various concentrations of ethanol following the fixed with 4% paraformaldehyde.

The slices were incubated with Zyto*Dot*SPEC MYC probe overnight and were targeted with anti‐digoxigenin antibody (NBP2‐31191; Novus). The signal of *MYC* locus was visualized using EAL™ DAB+ Chromogen diluted in REAL Substrate Buffer (Dako) following the treatment of the secondary antibody (ENV, Dako). Hematoxylin was used for nuclear staining. The dehydration, mounting process and detailed process are described as Supporting Information.

### 2.22 In situ hybridization detection of miRNAs

The IsHyb In Situ Hybridization Kit (BioChain) was used to detect the level of hsa‐miR‐429 according to the manufacturer's instructions. Briefly, the FFPE slides proceeded to the process of deparaffinization and rehydration. After the fixation of 4% Diethyl pyrocarbonate (DEPC)‐paraformaldehyde, the slices were treated with a prehybridization solution followed by the incubation of digoxigenin‐labeled RNA oligo probe against hsa‐miR‐429 (Exiqon). The signal of hsa‐miR‐429 was visualized following the incubation with AP‐conjugated anti‐digoxigenin antibody and a mixture of nitro‐blue tetrazolium and 5‐bromo‐4‐chloro‐3‐indolyl phosphate (BCIP). The nuclear Fast Red solution (Sigma) was used for the nuclear counterstaining. Mounting, dehydration and detailed procedures were indicated in Supporting Information.

### 2.23 Xenograft animal models

SCID/beige male mice were purchased from BioLASCO (Taiwan). The animal study was approved by the Institutional Animal Care Committee of Chi‐Mei Medical Center (Approval number 109041701). After one week of feeding the normal diet (OYDMFG22; BioLASCO, Taiwan), two of these four groups were switched to a high‐fat diet regimen (58Y1; TestDiet) for the duration of the study to induce type 2 diabetes mellitus (DM), as described in previous research.^27^ The body weight and fasting blood glucose of mice were robustly increased upon switching to a high‐fat diet (Figure S1). *Mock*‐ or *CEBPD*‐overexpressing BFTC909 cells mixed with high‐concentration Matrigel (354248; Corning Life Sciences, USA) were injected subcutaneously into groups with normal diet or high‐fat diet. The tumour diameters were measured at the indicated time using callipers and calculated by the following equation: V (mm^3^) = (π/6) × width (mm^2^) × length (mm). The mice were sacrificed with asphyxiation. The tumours were dissected and fixed in 10% formalin for further analysis.

### 2.24 Analysis of data from the NHIRD

We conducted a population‐based longitudinal observational cohort study in Taiwan from 2000 to 2010 to analyse whether DM comorbidity impacts the prognosis of UBUC and UTUC by using the NHIRD database. Catastrophic illness registry data were also included in this database, and comorbidity information of patients was collected. The inclusion criteria of the BLCA cohort group were as follows: age over 20 years old and UBUC diagnosis (International Classification of Disease [ICD]‐9 code 188.9) from 2000 to 2008 or UTUC diagnosis (ICD‐9 code 189.1 or 189.2). The exclusion criteria were as follows: age less than 20 years and incomplete demographic data. From both our BLCA and UTUC groups, patients with simultaneous DM (ICD‐9 code 250.X0 and 250.X2) were identified. After matching age, sex, index year, and all comorbidities, we included three times the number of patients without DM to use as a comparison group. The diagnosed comorbidities included chronic kidney disease (CKD) (ICD‐9 code 580–589), hypertension (ICD‐9 code 401–405), chronic obstructive pulmonary disease (COPD) (ICD‐9 code 490–496), stroke (ICD‐9 code 430–438), cardiovascular disease (ICD‐9 code 410–414), peripheral vascular disease (ICD‐9 code 250.7, 785.4, 443.81, 440–448), hyperlipidemia (ICD‐9 code 272.0‐272.4), urinary tract disease (ICD‐9 code 590–599), and benign prostatic hyperplasia (ICD‐9 code 600).

### 2.25 Statistics

The correlations and associations between targeted genes or proteins and clinicopathologic variables, as well as comparisons for various functional and animal studies, were evaluated by Spearman's rank correlation coefficient, Chi‐square test, or Mann‐Whitney U test as appropriate by using SPSS software (version 14.0; IBM, USA). Disease‐specific survival (DSS) and metastasis‐free survival (MeFS) were estimated and plotted by means of log‐rank tests and Kaplan‐Meier curves, respectively. A multivariate Cox proportional hazards model was used to evaluate the independent prognostic impacts of selected parameters. Kaplan‐Meier curves were drawn and the cumulative survival rate and log‐rank statistic for the NHIRD data were assessed by using SAS version 9.3 (SAS Institute, Cary, NC). Both univariate and multivariate Cox proportional hazards regressions were performed after adjusting for comorbidities to measure the risk of UBUC and UTUC. For all analyses, differences with a two‐tailed *p*‐value lower than .05 were considered significant.

## 3 RESULTS

### 3.1 CEBPD confers mTORC1‐driven metabolic reprogramming and glucose addiction in UC

**FIGURE 1:**
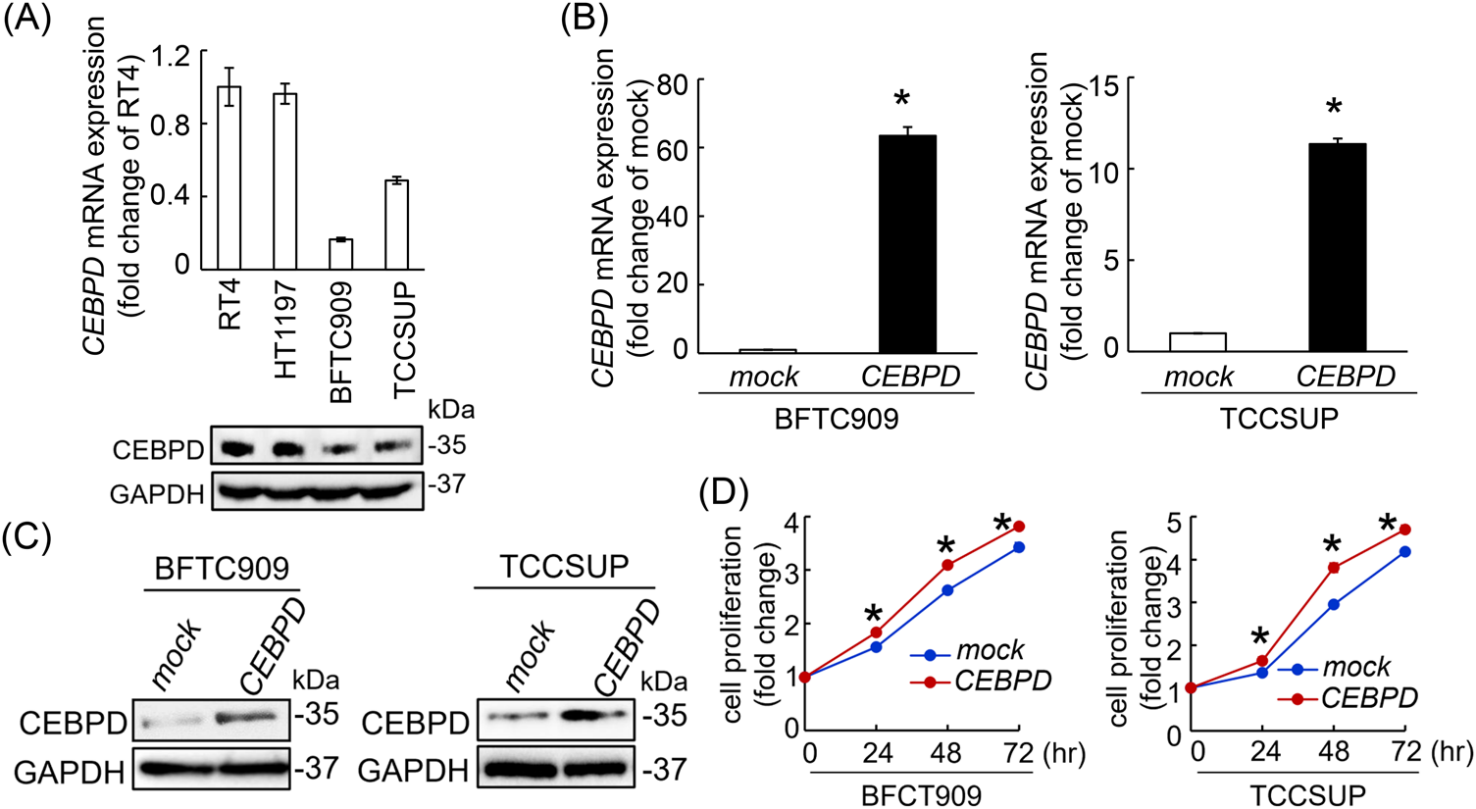
Overexpression of CCAAT/enhancer‐binding protein delta (CEBPD) confers a pro‐proliferative phenotype in BFTC909 and TCCSUP cells. Quantitative reverse transcription‐polymerase chain reaction (RT‐PCR) and immunoblotting (A) showed that the transcript and protein levels of CEBPD were lower in the BFTC909 and TCCSUP cells among the four urothelial carcinoma (UC)‐derived cell lines. Hence, Exogenous CEBPD expression was performed in BFTC909 and TCCSUP cells to examine the biological impact of CEBPD on tumorigenesis. The mRNA (B) and protein (C) levels of CEBPD were significantly upregulated in BFTC909 and TCCSUP cells after successful exogenous expression of the *CEBPD* gene compared with the mock‐transfected cell lines. (D) Proliferation assay showed that overexpression of CEBPD in BFTC909 and TCCSUP significantly increased pro‐proliferative phenotype at 24–72 h after seeding compared to the mock‐transfected cells. All experiments were conducted in triplicate and results were represented as the mean ± SEM. For immunoblot assay, one representative image was shown and GAPDH was regarded as a loading control. Statistical significance: * *p* < .05

To clarify the oncogenic property of CEBPD, the expression of CEBPD on clinical specimens was examined. A higher level of *CEBPD* transcript was observed in the UCs compared to their non‐tumour counterparts. Particularly, the mRNA level of *CEBPD* showed a stepwise escalation from non‐tumour urothelium, non‐muscle invasive bladder cancer (NMIBC), to muscle invasive bladder cancer (MIBC) (NMIBC vs. Normal, *p *< .001; MIBC vs. Normal, *p *= .048; MIBC vs. NMIBC, *p *= .006; Figure S2A). Consistently, UC specimens also represented higher CEBPD protein levels than non‐tumour urothelium (Figure S2B). In the following experiments, we utilized four UC‐derived cell lines (RT4, HT1197, BFTC909 and TCCSUP) to explore the molecular mechanism underlying the poor prognostic impact of CEBPD. BFTC909 and TCCSUP cells had the lowest level of CEBPD (Figure 1A); therefore, CEBPD overexpression was induced in these cell lines. Compared to lentiviral infection with the mock sequence, lentiviral infection with the *CEBPD* gene significantly increased the mRNA and protein levels of CEBPD in BFTC909 and TCCSUP cells (Figure 1B,C). We previously identified that overexpression of CEBPD contributes to increased cell viability in UC‐derived cell lines.^28^ Furthermore, higher cell proliferation was also observed in CEBPD‐overexpressing BFTC909 and TCCSUP cells than in mock‐transfected BFTC909 and TCCSUP cells (Figure 1D).

**FIGURE 2:**
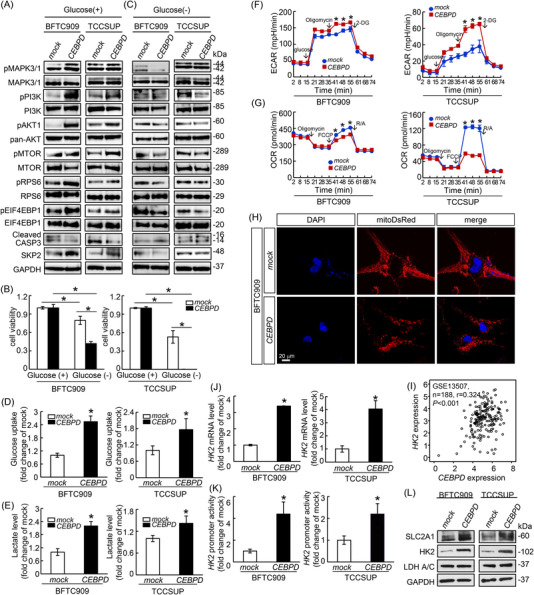
CCAAT/enhancer‐binding protein delta (CEBPD) overexpression confers mTORC1‐driven metabolic reprogramming and leads to glucose addiction. The potential relationship between CEBPD and proteins associated with the mTOR pathway was explored by immunoblotting analysis. (A) pMAPK3/1, pPI3K, pAKT1, pMTOR, pRPS6, pEIF4EBP1 and SKP2 were significantly upregulated, while cleaved CASP3 was notably decreased in CEBPD‐overexpressing BFTC909 and TCCSUP cells treated with culture medium containing glucose compared to mock‐expressing cells. (B) 2,3‐Bis‐(2‐methoxy‐4‐nitro‐5‐sulfophenyl) 2H‐tetrazolium‐5‐carboxanilide (XTT) assay indicated that cell viability was notably inhibited in mock‐expressing BFTC909 and TCCSUP cells under glucose starvation (culture medium without glucose) for 72 h compared to mock‐expressing cells in a complete cell culture medium. Furthermore, CEBPD overexpression further exacerbated glucose withdrawal‐induced cell viability suppression in these two cell lines. (C) Immunoblotting data indicated that glucose deficiency suppresses the effect of CEBPD on the upregulation of pAKT1, pMTOR, pRPS6, pEIF4EBP1 and SKP2 protein and the downregulation of CASP3 in these two distinct UC‐derived cells. However, the protein levels of pMAPK3/1 and pPI3K were not comparable in CEBPD‐overexpressing BFTC909 and TCCSUP cells under glucose starvation conditions. Glucose uptake assay (D), lactate level analysis (E), Seahorse XFp Analyzer assessment (F–G), OCR, and MitoDsRed‐tagged fluorescent mitochondrial staining (H) were performed to evaluate the cellular glucose uptake, lactate production, oxygen consumption rate (OCR), extracellular acidification rate (ECAR) and mitochondrial status. The data showed that stable overexpression of CEBPD notably increased glucose uptake (D), lactate production (E), the ECAR (F) and mitochondrial fragmentation/fission (H) but decreased the OCR (G) in BFTC909 and TCCSUP cells compared to mock‐expressing cells. To clarify the metabolic switch from mitochondrial oxidative phosphorylation (OXPHOS) to glycolysis under CEBPD regulation, the relationship between *CEBPD* and hexokinase 2 (*HK2*) was estimated through analysis of the Gene Expression Omnibus (GEO) database (GSE13507 dataset, *n*  =  188), indicating a significantly positive correlation between the mRNA levels of *CEBPD* and *HK2* in bladder cancer specimens (I). The result was validated by quantitative reverse transcription‐polymerase chain reaction (RT‐PCR) (J), promoter activity assay (K), and immunoblotting (L) and indicated that the transcript (J) and protein (L) levels of HK2 were markedly upregulated in CEBPD‐overexpressing BFTC909 and TCCSUP cells versus mock‐transfected cells via the upregulation of *HK2* promoter activity (K). Additionally, CEBPD overexpression increased the protein level of SLC2A1 but not LDH A/C (L). All experiments were performed in triplicate and data was represented as the mean ± SE. For immunoblot assay and fluorescent image data, one representative image is displayed. GAPDH was served as a loading control for immunoblot assay. Statistical significance: * *p* < .05

Given that mTOR is a master regulator of cell growth,^29^, ^30^ the potential correlations between CEBPD and mTOR signalling‐related proteins were examined. The phosphorylation levels of mTOR and its upstream activators, including MAPK3/1, PI3K, and AKT1, as well as downstream regulators, including RPS6 and EIF4EBP1, were notably upregulated, while the expression level of cleaved CASP3, a marker of apoptosis, was downregulated in CEBPD‐overexpressing BFTC909 and TCCSUP cells (Figure 2A). Moreover, increased expression of CEBPD promoted the expression of S‐phase kinase‐associated protein 2 (SKP2) (Figure 2A), which is an E3 ubiquitin ligase that activates AKT1 through the ubiquitination process.^30^ The above data suggest that CEBPD activates mTOR downstream targets through MAPK3/1‐ and PI3K/AKT‐dependent pathways.

mTOR signalling is one of the major hubs of the glucose‐sensing pathway, and large amounts of glucose are essential for tumour progression. Glucose deprivation is related to the inhibition of mTORC1 activity, anabolic processes, cell proliferation and the cause of cell death.^31^, ^32^, ^33^ Therefore, the impact of CEBPD on the cell survival and downstream regulation of the mTOR pathway under glucose‐deprived conditions was examined. The cell viability of mock‐infected BFTC909 and TCCSUP was notably decreased after glucose deprivation (Figure 2B). However, CEBPD overexpression exacerbated glucose deficiency‐induced cell viability inhibition (Figure 2B). CEBPD overexpression also failed to induce the increased pAKT1, pMTOR, pRPS6, pEIF4EBP1 and SKP2 after the glucose deprivation in these two distinct UC cells. (Figure 2C). Nevertheless, the effects of glucose deprivation on pMAPK3/1 and pPI3K protein levels were not comparable in CEBPD‐overexpressing BFTC909 and TCCSUP cells. Cleaved CASP3 was upregulated under glucose‐deficient conditions in both CEBPD‐overexpressing cell lines. In cell cycle analysis, exogenous CEBPD inhibited the sub G1 fraction in the BFTC909 and TCCSUP treated with general culture medium (Figure S3A,B,E–G,J). Reversely, CEBPD overexpression increased a sub G1 population in cells under glucose deprivation than in mock‐group (Figure S3C–E,H–J). Additionally, exogenous CEBPD also led to S phase arrest in the glucose‐deprived BFTC909 and TCCSUP (Figure S3C–E,H–J). The distribution of cells in the G1 or G2 phase was not comparable in mock‐ and CEBPD‐infected BFTC909 and TCCSUP treated with general culture medium or glucose deprivation (Figure S3). Annexin V/PI assay showed the apoptotic cells were mildly decreased after CEBPD overexpression in BFTC909 and TCCSUP (Figure S4A,B,E–G,J) while were drastically escalated in CEBPD‐infected cells with glucose removal (Figure S4C,D,H–J). These data suggested that UC‐derived cell lines with CEBPD overexpression are more susceptible to glucose deprivation (i.e., these cells exhibit glucose addiction) and could result in the suppression of mTORC1 activity, retarded cell proliferation and increased apoptosis.

We next aimed to examine the effect of exogenous CEBPD overexpression in BFTC909 and TCCSUP cells on cell metabolism. Compared with the mock‐transfected cells, CEBPD‐overexpressing BFTC909 and TCCSUP cells showed increases in glucose uptake (Figure 2D), cellular lactate production (Figure 2E), and the ECAR (Figure 2F), a decrease in the cellular OCR (Figure 2G), and a shift from mitochondrial fusion to fission (Figure 2H). These findings suggest that CEBPD‐overexpressing cells undergo glycolysis and a metabolic switch from mitochondrial respiration to aerobic glycolysis.

Increased glycolysis is inevitably driven by the upregulation of the glycolysis‐related gene profile which consists of solute carrier family 2 member 1 (*SLC2A1*, also known as glucose transporter 1, *GLUT‐1*) and Hexokinase 2 (*HK2*) as the major elements.^34^ Of these, *HK2*, which encodes the enzyme that catalyses the formation of glucose 6‐phosphate (G6P) from glucose in the first step in glycolysis, is a critical regulator of aerobic glycolysis.^35^ Accordingly, we tried to explore whether the preference of oxidative phosphorylation (OXPHOS) to aerobic glycolysis promoted by CEBPD is linked to the dysregulation of glycolysis‐related gene profile, with special attention to *HK2* expression. An analysis of BLCA specimens in the Gene Expression Omnibus Database (GSE13507 dataset, *n*  =  188) using the Oncomine Research Premium Edition tool (http://oncomine.org) revealed a marked positive correlation between the mRNA levels of *CEBPD* and *HK2* (r = 0.324, *p* < .001; Figure 2I). We then examined the mRNA level of these two genes in BFTC909 and TCCSUP cells, and the mRNA level and promoter activity of *HK2* were increased upon CEBPD overexpression (Figure 2J,K). Western blotting showed that the overexpression of exogenous CEBPD markedly elevated the protein levels of HK2 and SLC2A1 but not that of lactate dehydrogenase A/C (LDH A/C) in BFTC909 and TCCSUP cells (Figure 2L), indicating that CEBPD increases the expression of SLC2A1 and HK2 to meet the demands of rapid glycolytic flux. These data disclosed that CEBPD enhances glucose influx through the upregulation of SLC2A1 and HK2 to fuel the mTOR signalling under glucose addiction which is conferred by the metabolic reprogramming from mitochondrial respiration to aerobic glycolysis triggered by mTOR signalling.

### 3.2 Positive crosstalk between CEBPD and MYC in UC

**FIGURE 3:**
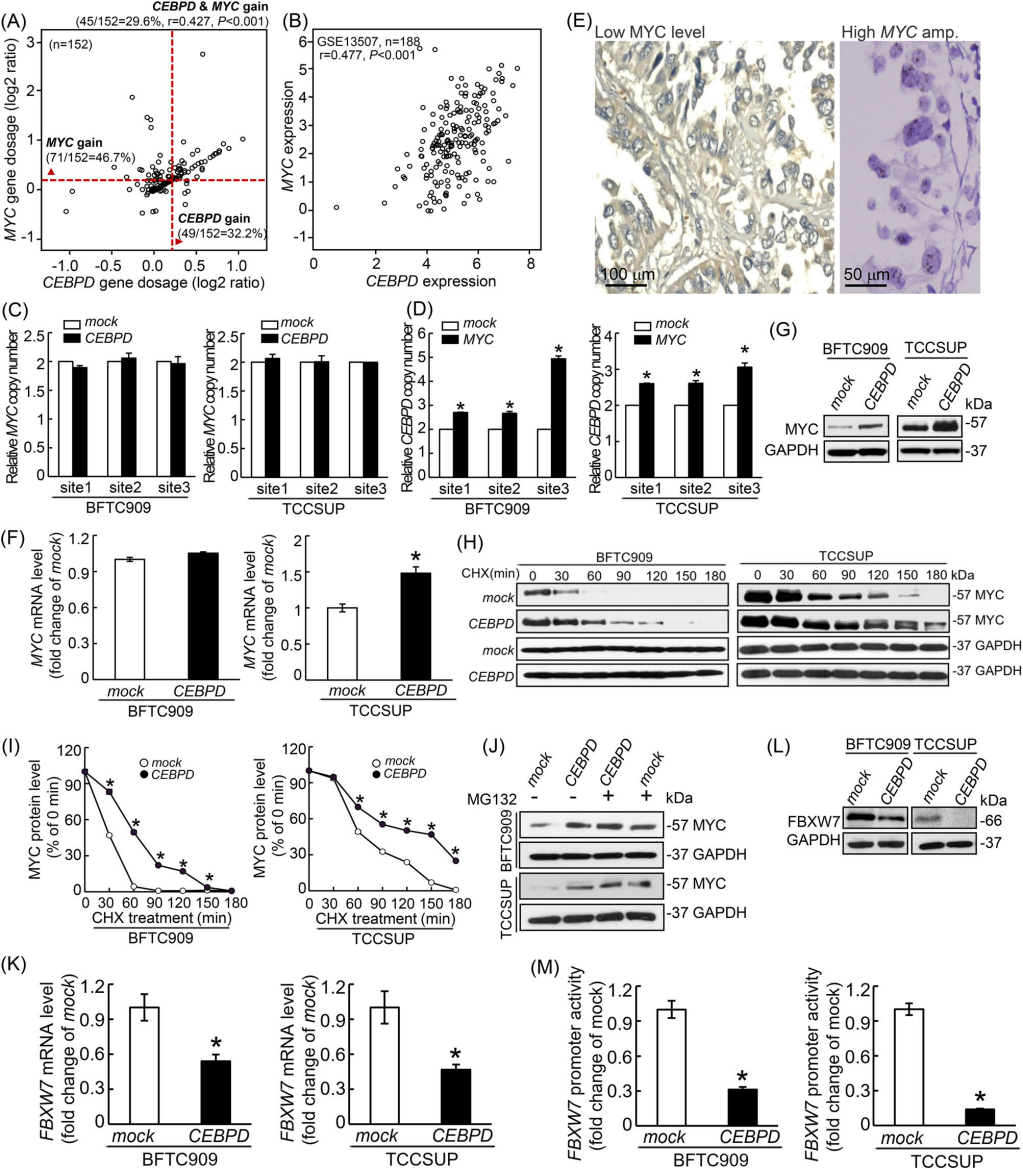
Collaboration of CCAAT/enhancer‐binding protein delta (CEBPD) and MYC in the regulation of SLC2A1 and HK2. (A) To assess the crosstalk between the dosage/gene expression of *CEBPD* and *MYC*, we reassessed The Cancer Genome Atlas (TCGA) bladder cancer dataset and found that 32.2% (49/152) and 46.7% (71/152) of urothelial carcinomas (UCs) showed copy number gains (CNGs) involving *CEBPD* and *MYC*, respectively. Moreover, a notably positive correlation (45/152, *r* = 0.427, *p *< .001) between *CEBPD* and *MYC* dosage was observed. The Red dashed line indicated the log2 ratio of 0.2, which represent the cut‐off of copy number gain. (B) Assessment of the GEO database (GSE13507 dataset, n = 188) identified a markedly positive relation between the transcript levels of *MYC* and *CEBPD* (*r* = 0.477, *p* < .001). Copy number variation (CNV) profiled by real‐time PCR using three distinct probes targeting three different regions of the *MYC* and *CEBPD* chromosomes indicated that the overexpression of MYC through viral delivery systems could induce *CEBPD* amplification in BFTC909 and TCCSUP (D) cells but not vice versa (C). (E) Immunohistochemistry (IHC; left) and chromogenic in situ hybridization (CISH; right) assays revealed an inconsistency between the expression and gene dosage of *MYC* in a representative case of UTUC with *MYC* amplification but a low MYC expression. (F) The effect of CEBPD overexpression on the promotion of *MYC* transcription was not comparable in BFTC909 and TCCSUP cells, while (G) CEBPD overexpression robustly increased the protein level of MYC in both cell lines, suggesting the possibility that CEBPD exerts posttranslational regulation of MYC expression. (H) Evaluation of protein stability by cycloheximide (CHX) chase assays coupled with immunoblotting shows that MYC protein expression was restored in CEBPD‐expressing BFTC909 and TCCSUP cells compared to mock cells. The statistical results were shown in a line graph (I). Moreover, the MYC protein levels were abundantly increased in the mock‐transfected BFTC909 and TCCSUP groups treated with MG132, a ubiquitin‐proteasome inhibitor, compared to the vehicle‐treated group. MYC protein had the same effect between MG132 or vehicle treatment in CEBPD‐overexpressing BFTC909 and TCCSUP cells (J). CEBPD overexpression notably inhibited the mRNA (K) and protein (L) levels of FBXW7 in BFTC909 and TCCSUP cells. (M) CEBPD overexpression significantly suppressed *FBXW7* promoter activity in BFTC909 and TCCSUP cells, confirming that CEBPD upregulates MYC expression by depleting FBXW7‐mediated MYC degradation. All experiments were executed in triplicate and the results were represented as the mean ± SEM. For immunoblot assay, IHC and CISH data, one representative image was displayed. GAPDH was shown as a loading control for immunoblot assay. Statistical significance: * *p* < .05

MYC has been also elucidated to involve in the upregulation of key glycolysis‐related genes including SLC2A1 and HK2,^36^ therefore, it appeared that some kind of interplay between CEBPD and MYC on glycolysis. To clarify the correlation between CEBPD and MYC, we reanalysed our previously published aCGH data,^10^ aside from the amplification of *CEBPD*, copy number gain (CNG) of the *MYC*, which is also mapped to chromosome 8q24.21, was also preferentially detected in UBUC patients who experienced disease‐specific death (69.23% vs. 18.52% in patients with no events, *p *= .0034) and distal metastasis (75.0% vs. 8.33% patients with no events, *p *< .0001) and was highly associated with *CEBPD* CNG (*p *< .001) (Table S2). Reassessment of the TCGA dataset through the Oncomine platform showed that 32.2% (49/152) and 46.7% (71/152) of UBUC samples had CNGs (log2 ratios ≥ 0.2) involving *CEBPD* and *MYC*, respectively, and there was a significant positive correlation between the *CEBPD* and *MYC* gene dosages (*r *= 0.427, *p *< .001) (Figure 3A and Figure S5). Moreover, there was a significant positive correlation between *CEBPD* and *MYC* transcript expression in UBUC (GSE13507, *n* = 188, *r* = 0.477, *p *< .001) (Figure 3B and Figure S6). By using fluorescence in situ hybridization, CISH and IHC, we also identified significant positive correlations between *CEBPD* CNG/expression and *MYC* CNG/expression in our UBUC and UTUC cohort (Tables 1 and 2). Nevertheless, CEBPD overexpression in BFTC909 and TCCSUP cells did not induce *MYC* CNG (Figure 3C), but elevated *CEBPD* CNG was observed upon MYC overexpression (Figure 3D), implying that *MYC* overexpression precedes *CEBPD* CNG and that overexpression is probably driven by *MYC*‐mediated chromosomal instability (CIN).

**TABLE 1:** Correlations between CCAAT/enhancer‐binding protein delta (CEBPD) and MYC amplification/expression and miR‐429 and HK2 expression and other important clinicopathological parameters in urinary bladder urothelial carcinoma (UBUC)

**Parameter**	**Category**	**Case No**.	***MYC* amplification**			**MYC expression**			**miR‐429 expression**			**HK2 expression**		
			**Non‐amp**	**Amp**	***p*‐value**	**Low**	**High**	***p*‐value**	**Low**	**High**	***p*‐value**	**Low**	**High**	***p*‐value**
Sex^&^	Male	216	175	41	.627	153	63	.825	138	78	.262	165	51	.937
	Female	79	62	17		57	22		56	23		60	19	
Age (years) ^&^	<65	121	95	26	.510	84	37	.577	79	42	.886	94	27	.634
	≥65	174	142	32		126	48		115	59		113	43	
Diabetes mellitus^&^	Negative	240	193	47	.944	169	71	.542	154	86	.288	186	54	.300
	Positive	55	44	11		41	14		40	15		39	16	
Primary tumor (T)^&^	Ta	84	84	0	**<.001** *	79	5	**<.001** *	20	64	**<.001** *	80	4	**<.001** *
	T1	88	82	6		72	16		56	32		73	15	
	T2–T4	123	71	52		59	64		118	5		72	51	
Nodal metastasis^&^	Negative (N0)	266	222	44	**<.001** *	198	68	**<.001** *	166	100	**<.001** *	212	54	**<.001** *
	Positive (N1–N2)	29	15	14		12	17		28	1		13	16	
Histological grade ^&^	Low grade	56	55	1	**<.001** *	48	8	**.008** *	15	41	**<.001** *	50	6	**.011** *
	High grade	239	182	57		162	77		179	60		175	64	
Vascular invasion^&^	Absent	246	207	39	**<.001** *	190	56	**<.001** *	147	99	**<.001** *	197	49	**.001** *
	Present	49	30	19		20	29		47	2		28	21	
Perineural invasion^&^	Absent	275	225	50	**.018** *	203	72	**<.001** *	174	101	**.001** *	214	61	**.021** *
	Present	20	12	8		7	13		20	0		11	9	
Mitotic rate (per 10 high power fields)^#^		295	13.8 +/−14.22	16.8 +/−13.11	**.035** *	13.7 +/−14.51	16.4 +/−12.66	**.013** *	16.1 +/−13.39	11.1 +/−14.70	**<.001** *	13.2 +/−14.15	18.3 +/−13.02	**<.001** *
*CEBPD* gene amplification^&^	Non‐amp	232	208	24	**<.001** *	199	33	**<.001** *	135	97	**<.001** *	204	28	**<.001** *
	Amp	62	29	34		11	52		59	4		21	42	
CEBPD expression^&^	Low expression	207	187	20	**<.001** *	193	14	**<.001** *	117	90	**<.001** *	197	10	**<.001** *
	High expression	88	50	38		17	71	–	11	11		28	60	
*MYC* amplification^&^	Non‐amp	237				188	49	**<.001** *	140	97	**<.001** *	195	42	**<.001** *
	Amp	58				22	36		54	4		30	28	
MYC expression^&^	Low expression	210							115	95	**<.001** *	192	18	**<.001** *
	High expression	85							79	6		33	52	
miR‐429 expression^&^	Low expression	194										131	63	**<.001** *
	High expression	101										94	7	

^&^ Chi‐square test.

^#^ Mann‐Whitney U test; all other comparisons.

* Statistical significance. Tumour samples were taken from the biobank of Chi Mei Medical Center.

**TABLE 2:** Correlations between CCAAT/enhancer‐binding protein delta (CEBPD) and MYC amplification/expression and miR‐429 and HK2 expression and other important clinicopathological parameters in upper urinary tract urothelial cancer (UTUC)

**Parameter**	**Category**	**Case No**.	***MYC* amplification**			**MYC expression**			**miR‐429 expression**			**HK2 expression**		
			**Non‐amp**	**Amp**	***p*‐value**	**Low**	**High**	***p*‐value**	**Low**	**High**	***p*‐value**	**Low**	**High**	***p*‐value**
Sex^&^	Male	158	129	29	.554	122	36	.763	117	41	.539	119	39	.640
	Female	182	153	29		143	39		140	42		141	41	
Age (years) ^&^	<65	138	112	26	.470	107	31	.882	96	42	**.033** *	102	36	.358
	≥65	202	170	32		158	44		161	41		158	44	
Diabetes mellitus^&^	Negative	276	230	46	.690	216	60	.768	202	74	**.032** *	212	64	.758
	Positive	64	52	12		49	15		55	9		48	16	
Multifocality^&^	Single	273	231	47	.874	219	59	.431	206	72	.176	215	63	.425
	Multifocal	62	51	11		46	16		51	11		45	17	
Primary tumor (T) ^&^	Ta	89	82	7	**<.001** *	84	5	**<.001** *	38	51	**<.001** *	75	14	**<.001** *
	T1	92	83	9		79	13		88	4		83	9	
	T2–T4	159	117	42		102	57		131	28		102	57	
Nodal metastasis^&^	Negative (N0)	312	267	45	**<.001** *	254	58	**<.001** *	232	80	.078	246	66	**.001** *
	Positive (N1–N2)	28	15	13		11	17		25	3		14	14	
Histological grade ^&^	Low grade	56	54	2	**.003** *	52	4	**.003** *	25	31	**<.001** *	51	5	**.005** *
	High grade	284	228	56		213	71		232	52		209	75	
Vascular invasion^&^	Absent	234	205	29	**.001** *	203	31	**<.001** *	165	69	**.001** *	191	43	**.001** *
	Present	106	67	39		62	44		92	14		69	37	
Perineural invasion^&^	Absent	321	271	50	**.003** *	259	62	**<.001** *	239	82	**.046** *	253	68	**<.001** *
	Present	19	11	8		6	13		18	1		7	12	
Mitotic rate (per 10 high power fields)^#^		340	12.2 +/− 12.49	12.7 +/−11.31	.474	11.9 +/−12.21	13.7 +/−12.51	.090	12.8 +/−11.82	10.9 +/−13.50	**.013** *	12.0 +/−13.02	12.4+/−9.48	**.010** *
*CEBPD* gene amplification^&^	Non‐amp	264	256	8	**<.001** *	253	11	**<.001** *	184	80	**<.001** *	239	25	**<.001** *
	Amp	76	26	50		12	64		73	3		21	55	
CEBPD expression^&^	Low expression	251	243	8	**<.001** *	239	12	**<.001** *	170	81	**<.001** *	240	11	**<.001** *
	High expression	89	39	50		26	63	–	87	2		20	69	
*MYC* amplification^&^	Non‐amp	282				258	24	**<.001** *	203	79	**.001** *	245	37	**<.001** *
	Amp	58				7	51		54	4		15	43	
MYC expression^&^	Low expression	265							187	78	**<.001** *	238	27	**<.001** *
	High expression	75							70	5		22	53	
miR‐429 expression^&^	Low expression	257										188	69	**.011** *
	High expression	83										72	11	

^&^ Chi‐square test.

^#^ Mann‐Whitney U test.

* Statistical significance. Tumour samples were taken from the biobank of Chi Mei Medical Center.

To determine whether MYC enhances chromosomal instability, we compared the LOH of cell lines with and without exogenous MYC overexpression using the targeted NGS approach. Interestingly, with the targeted NGS focusing on 24 HRR‐related genes, we identified several mutations in the parental BFTC909 cell including *BRIP1* p.N196S (VUS), *BRCA1* exon 1–3 replication, and *CDK12* heterozygous deletion (Tables S3 and 4) but none in the parental TCCSUP cell. Accordingly, we used the TCCSUP cell as a model to computerize MYC‐enhancing chromosomal instability to avoid confounders. The analysis of heterozygous SNPs showed that MYC overexpression leads to a 17.5% and 9.0% increase in the genomic LOH in the BFTC909 and TCCSUP cell lines, respectively. (Table S5). The above‐mentioned findings suggested MYC can drive CIN in UC that probably the causal factor of MYC‐associated *CEBPD* gain in UC.

To systemically exclude the possibility that *MYC* upregulates *CEBPD* directly through long‐range chromatin interaction, we performed NGS‐based Hi‐C experiments. The Capture Hi‐C were processed using HiC‐Pro pipeline, the raw contact frequency was normalized using iterative correction and eigenvector decomposition method, and the normalized interactions map were visualized at 40 kb bin size resolution using the HiTC R package (Figure S7), which showed there are no predicted intra‐TAD loops between *CEBPD* and *MYC* gene in both TCCSUP and BFTC909 cells.

The expression of MYC is not always proportional to the level of the *MYC* dosage in UC.^37^ In line with the previous study, we also found that 37.9% (22 out of 58) of *MYC*‐amplified UBUCs and 12.1% (7 out of 58) of *MYC*‐amplified UTUCs showed low MYC expression, while 20.7% (49 out of 237) of *MYC*‐nonamplified UBUCs and 8.5% (24 out of 282) of *MYC*‐nonamplified UTUCs showed high MYC expression (Tables 1 and 2 and Figure 3E), suggesting that amplification‐independent mechanisms regulate MYC expression. In vitro, we also identified that the amount of upregulated MYC protein in CEBPD‐overexpressing BFTC909 and TCCSUP is not comparable to those of the mRNA level (Figure 3F,G), which indicates that CEBPD proceeds through an alternative pathway apart from transcriptional regulation to manage MYC expression. Therefore, the effect of CEBPD on MYC protein stability was measured using a cycloheximide (CHX) assay. Compared to that in mock‐transfected cells, the half‐life of endogenous MYC protein after treatment with CHX in CEBPD‐overexpressing BFTC909 and TCCSUP cells was notably prolonged (Figure 3H,I). Furthermore, the ubiquitin‐proteasome inhibitor MG132 significantly prevented the degradation of the MYC protein in the mock‐transfected cells compared with the vehicle‐treated mock‐transfected cells, but the effect of MG132 on the MYC protein level was mild in CEBPD‐overexpressing cells (Figure 3J). Based on the above findings, CEBPD could stabilize MYC protein expression by protecting MYC from ubiquitin‐mediated proteasomal degradation instead of regulating the transcription of the *MYC* gene.

Next, given that FBXW7, a critical substrate recognition component of the SKP1‐CUL1‐F‐box protein (SCF) E3 ubiquitin‐protein ligase complex that mediates proteasomal degradation through phosphorylation‐dependent ubiquitination, is well known to target important oncoproteins such as MYC,^38^, ^39^, ^40^ we next clarified whether CEBPD stabilizes MYC by inhibiting FBXW7. The mRNA and protein levels of FBXW7 and the *FBXW7* promoter activity were strikingly decreased after CEBPD overexpression in BFTC909 and TCCSUP cells (Figure 3K–M), suggesting that CEBPD directly suppresses FBXW7 through transcriptional regulation. These data indicate that chromosomal instability of *MYC* and *CEBPD* is concomitant and represents a positive correlation in UC. We also disclosed that there is a potent positive feedback loop between MYC and CEBPD to collaboratively regulate oncogenesis: Upregulated expression of CEBPD following the *CEBPD* amplification could be subsequent to the *MYC*‐dependent genome instability. In addition, increased CEBPD also displayed a protective role to stabilize the protein level of MYC through the inhibition of the FBXW7‐mediated proteasomal degradation.

### 3.3 CEBPD promotes the level of HK2 via inhibition of hsa‐miR‐429 and is partially independent of MYC regulation

**FIGURE 4:**
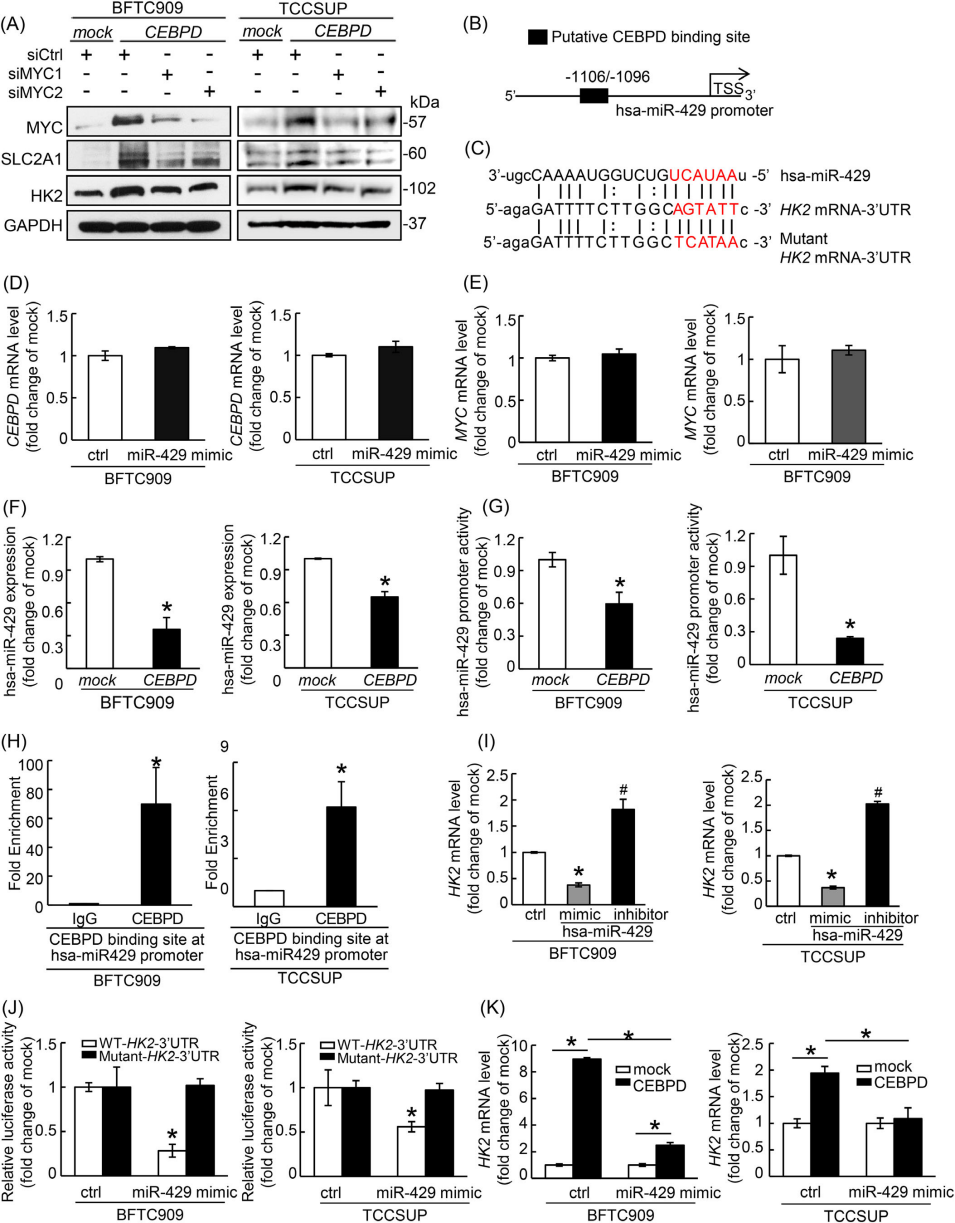
CCAAT/enhancer‐binding protein delta (CEBPD) promotes HK2 expression through the transcriptional suppression of hsa‐miR‐429. (A) Immunoblotting assays confirmed that MYC, SLC2A1 and HK2 expression was markedly increased by exogenous CEBPD expression in BFTC909 and TCCSUP cells. The increase in MYC, SLC2A1 and HK2 expression induced by CEBPD overexpression was partially attenuated by two distinct MYC siRNAs in BFTC909 and TCCSUP cells, confirming the critical role of MYC in CEBPD‐driven glycolysis. (B) A putative CEBPD binding site at the hsa‐miR‐429 promoter was predicted by the JASPAR database (http://jaspar.genereg.net/). (C) Moreover, a predicted targeting site of hsa‐miR‐429 on the 3′‐untranslated region (3′‐UTR) of *HK2* mRNA was identified. The red font indicated the seed sequence of miRNA to the mRNA. Lowercase letters mean unmatched position while capital letters showed the matched region between miRNA and mRNA. To evaluate the regulation of HK2 expression by hsa‐miR‐429, a mutant‐*HK2* mRNA‐3′‐UTR was constructed as indicated. We identified that treatment with the miR‐429 mimic did not affect *CEBPD* (D) or *MYC* (E) transcription in BFTC909 and TCCSUP cells. Exogenous CEBPD overexpression significantly inhibited the expression (F) and promoter transactivity (G) of hsa‐miR‐429 through quantitative RT‐PCR and luciferase reporter assays, respectively, in BFTC909 and TCCSUP cells. (H) Chromatin immunoprecipitation (ChIP) indicated that CEBPD is evidently recruited to the hsa‐miR‐429 promoter region in BFTC909 and TCCSUP cells, confirming the direct binding and transcriptional regulation of CEBPD on hsa‐miR‐429. (I) Interestingly, the transcription of HK2 was robustly diminished by treatment with the miR‐429 mimic but promoted after treatment with the hsa‐miR‐429 inhibitor in BFTC909 and TCCSUP cells. Moreover, miR‐429 mimic treatment significantly inhibited the relative luciferase activity of BFTC909 and TCCSUP cells transfected with pMIR‐WT‐*HK2*‐3′‐UTR ‐Luc but not pMIR‐mutant‐*HK2*‐3′‐UTR‐Luc (J). (K) The transcription of HK2 was increased in CEBPD‐overexpressing BFTC909 and TCCSUP cells compared to mock‐expressing cells. However, compared with the vehicle, the miR‐429 mimic robustly inhibited the increase in *HK2* transcripts in CEBPD‐overexpressing BFTC909 and TCCSUP cells. The aforementioned evidence revealed that CEBPD promotes HK2 expression through the transcriptional suppression of hsa‐miR‐429. All experiments were performed in triplicate and the results were represented as the mean ± SEM. For immunoblot assay, one representative image was displayed. GAPDH was shown as a loading control for immunoblot assay. Statistical significance: *^#^ *p* < .05

To evaluate the cooperative regulation of CEBPD and MYC on glycolytic‐related genes, the protein level of SLC2A1 and HK2 was examined after the CEBPD overexpression or/and the treatment of the MYC siRNAs. The protein levels of SLC2A1 and HK2 were elevated after CEBPD overexpression in BFTC909 and TCCSUP cells but were partially downregulated after transfection of cells with two distinct MYC siRNAs (siMYC1 and siMYC2) which substantially depleted the protein level of MYC (Figure 4A), indicating that CEBPD could also induce glycolysis‐related protein expression in MYC‐independent manners. Accordingly, other potential mechanisms by which CEBPD regulates the glycolytic pathway were further examined by RNA‐seq. In total, Top 7 miRNAs met the selection criteria (log2 ratio ← 0.3 in both cell lines). hsa‐miR‐429 was the most significantly downregulated in BFTC909 cells (log2 ratio ‐1.985) and was also significantly downregulated in TCCSUP cells (log2 ratio ‐0.439) as well as the most remarkable mean fold change (log2 ratio ‐1.2127) (Table S6). As hsa‐miR‐429 has been previously predicted to target the *HK2* gene based on the target‐prediction algorithm from miRanda (http://cbio.mskcc.org/miRNA2003/miranda.html), we then clarified the correlation between CEBPD and hsa‐miR‐429 and its relation to glycolysis. In silico analysis revealed one putative CEBPD binding site at the promoter of hsa‐miR‐429 (Figure 4B, based on http://jaspar.genereg.net/). Interestingly, not only *HK2* (Figure 4C) but also *CEBPD* and *MYC* were predicted or validated as potential targets of hsa‐miR‐429 by miRanda. Therefore, we first confirmed that the *CEBPD* and *MYC* transcripts were not altered by hsa‐miR‐429 in BFTC909 and TCCSUP cells (Figure 4D,E). Subsequently, the probable regulatory relationship between CEBPD and hsa‐miR‐429, which was predicted to regulate *HK2* expression, was further examined. An in vitro experiment revealed that CEBPD overexpression inhibits the expression and promoter activity of hsa‐miR‐429 in BFTC909 and TCCSUP cells (Figure 4F,G). ChIP assays showed that endogenous CEBPD was recruited to the putative CEBPD binding site at the hsa‐miR‐429 promoter region in both cell types (Figure 4H). In addition, *HK2* transcript was notably diminished after treatment with the hsa‐miR‐429 mimic but was upregulated after transfection with the hsa‐miR‐429 inhibitor in BFTC909 and TCCSUP cells (Figure 4I).

To clarify whether hsa‐miR‐429 inhibits the mRNA expression of *HK2* by directly binding to its 3′‐UTR, we cotransfected BFTC909 and TCCSUP cells with a miRNA negative control or an hsa‐miR‐429 mimic and WT‐ or mutant‐*HK2* 3′‐UTR firefly luciferase reporter (Figure 4B). Compared with the miRNA negative control, the hsa‐miR‐429 mimic robustly abolished the luciferase activity of the WT‐*HK2* 3′‐UTR in BFTC909 and TCCSUP cells, yet neither the miRNA negative control nor the hsa‐miR‐429 mimic induced the loss of mutant‐*HK2* 3′‐UTR luciferase activity (Figure 4J). Furthermore, CEBPD upregulated the mRNA level of *HK2* upon transfection with the miRNA negative control but failed to robustly induce *HK2* transcript expression after transfection of BFTC909 and TCCSUP cells with the hsa‐miR‐429 mimic (Figure 4K). These data suggest that CEBPD protects against the degradation of the *HK2* transcript through the directly transcriptional suppression of hsa‐miR‐429.

### 3.4 High expression levels of MYC, CEBPD and HK2 are significantly associated with low hsa‐miR‐429 expression levels, and these expression patterns predict poor outcomes in UBUC and UTUC patients

**FIGURE 5:**
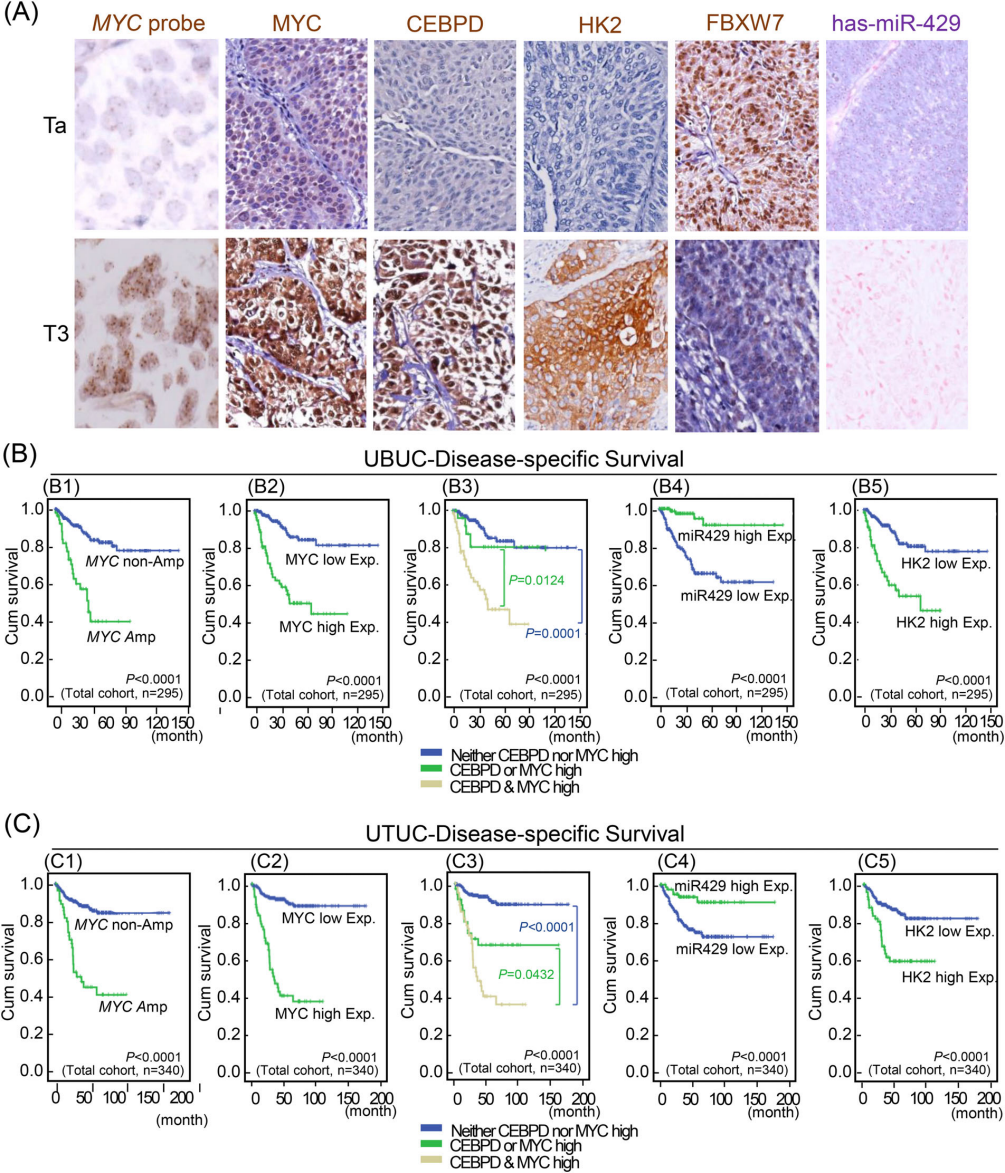
High expression of MYC, CCAAT/enhancer‐binding protein delta (CEBPD) and HK2 along with low FBXW7 and hsa‐miR‐429 expression predicts adverse features and poor patient outcomes in urinary bladder urothelial carcinoma (UBUC) and upper urinary tract urothelial cancer (UTUC). (A) IHC and in situ hybridization showed that *MYC* amplification; high expression of MYC, CEBPD, and HK2; and low expression of FBXW7 and hsa‐miR‐429 were strongly relevant to UBUC patients (*n* = 295) with high tumour stage. (B–C) Moreover, Kaplan‐Meier survival analysis indicated that both *MYC* amplification and high MYC expression are significant survival determinants in UBUC (*n *= 295; B1 and B2) and UTUC (*n* = 340; C1 and C2), while the survival impacts of MYC expression can be further enriched by the coexpression of CEBPD. (B3 and C3), suggesting potential synergistic effects of MYC and CEBPD in the promotion of UC progression. In addition, low expression of miR‐429 and high expression of HK2 also confer a poor prognosis in terms of disease‐specific survival in UBUC (*n* = 295; B4 and B5) and UTUC (*n* = 340; C4 and C5) patients. The UBUC and UTUC cohorts were taken from the biobank of Chi Mei Medical Center that collected specimens after an operation with curative intent between January 1996 and May 2004 as previously described.^22^ This study was approved by the institutional review board of Chi Mei Medical Center (IRB10207‐001). For immunohistochemistry, one representative image was shown upper urinary tract urothelial cancer (UTUC)

With a solid validation in our well‐characterized cohorts comprised of 295 UBUC patients and 340 UTUC patients as mentioned earlier,^22^ the present study disclosed *MYC* amplification, high *MYC* expression, high HK2 expression and low hsa‐miR‐429 expression were correlated with high pathological tumour (pT) stage, nodal metastasis, high histological grade, vascular and perineural invasion, a high mitotic rate and amplification/high expression of CEBPD. Moreover, *MYC* and *CEBPD* amplification and high expression and high HK2 expression showed significant positive correlations with each other but were negatively correlated with hsa‐miR‐429 expression (Tables 1 and 2, Figure 5A and Figure S8) (certain cases exhibited discordant MYC amplification and expression status). To explore the survival impacts of important clinicopathologic parameters and selected biomarkers, survival analysis was conducted at univariate and multivariate levels. In the univariate survival analysis, high pT stage; nodal metastasis; high histological grade; vascular invasion; perineural invasion; high mitotic rates; amplification of *CEBPD* or *MYC;* high expression of CEBPD, MYC, or HK2; low hsa‐miR‐429 expression and multifocality in UBUC and UTUC predicted poor DSS and MeFS (Figure 5B1,2, 4, 5 and C1,2,4,5; Tables 3 and 4). Additionally, when considering the status of both the CEBPD and MYC expression, the presence of both high MYC expression and high CEBPD expression indicated a more aggressive clinical course (Figure 5B3 and C3, Tables 3 and 4). In the multivariate analysis, UBUC patients with a high pT stage and a high mitotic rate and UTUC patients with multifocality and high MYC expression displayed worse DSS and MeFS than other patients (Tables 3 and 4). These data manifested that a high level of MYC, CEBPD, HK2 and low expression of hsa‐miR429 were frequently observed in aggressive UC with unfavourable survival outcomes. Especially, the combination of high expression of MYC and CEBPD represented the worst outcomes in patients with UBUC and UTUC.

**TABLE 3:** Univariate log‐rank and multivariate analyses for disease‐specific and metastasis‐free survival in urinary bladder urothelial carcinoma (UBUC)

			**Disease‐specific survival**					**Metastasis‐free survival**				
			**Univariate analysis**		**Multivariate analysis**			**Univariate analysis**		**Multivariate analysis**		
**Parameter**	**Category**	**Case No**.	**No. of events**	***p*‐value**	**RR**	**95% CI**	***p*‐value**	**No. of events**	***p*‐value**	**RR**	**95% CI**	***p*‐value**
Sex	Male	216	41	.4906	–	–	–	61	.2745	–	–	–
	Female	79	11		–	–	–	16		–	–	–
Age (years)	<65	121	17	.1315	–	–	–	32	.8786	–	–	–
	≥65	174	35		–	–	–	45		–	–	–
Diabetes mellitus	Negative	240	39	.0547	–	–	–	59	.0638	–	–	–
	Positive	55	13		–	–	–	18		–	–	–
Primary tumor (T)	Ta	84	1	**<.0001** *	1	–	**.001** *	4	**<.0001** *	1	–	**.010** *
	T1	88	9		4.276	0.448–40.851		23		3.543	1.021–12.295	
	T2–T4	123	42		15.956	1.673–152.217		50		5.391	1.493–19.466	
Nodal metastasis	Negative (N0)	266	41	**.0001** *	1	–	0.733	61	**<.0001** *	1	–	.072
	Positive (N1–N2)	29	11		1.134	0.551–2.332		16		1.756	0.951–3.243	
Histological grade	Low grade	56	2	**.0016** *	1	–	0.973	5	**.0007** *	1	–	.785
	High grade	239	50		0.972	0.190–4.982		72		1.158	0.404–3.326	
Vascular invasion	Absent	246	37	**.0010** *	1	–	0.148	54	**<.0001** *	1	–	.895
	Present	49	15		0.597	0.297–1.200		23		0.962	0.537–1.721	
Perineural invasion	Absent	275	44	**<.0001** *	1	–	0.093	67	**.0003** *	1	–	.155
	Present	20	8		2.117	0.881–5.085		10		1.748	0.809–3.774	
Mitotic rate (per 10 high power fields)	<10	139	12	**.0001** *	1	–	**.031** *	23	**<.0002** *	1	–	**.050** *
	≥10	156	40		2.110	1.071–4.156	**–**	54		1.660	1.000–2.757	**–**
CEBPD amplification	Nonamplified	232	30	**<.0001** *	–	–	**–**	42	**<.0001** *	–	–	**–**
	Amplified	63	22		–	–	**–**	35		–	–	**–**
CEBPD expression	Low	207	22	**<.0001** *	1	–	0.430	32	**<.0001** *	1	–	**.003** *
	High	88	30		1.475	0.561–3.877		45		2.971	1.453–6.075	
MYC amplification	Low	232	30	**<.0001** *	–	–	–	47	**<.0001** *	–	–	**–**
	High	63	22		–	–	–	30		–	–	**–**
MYC expression	Low	210	19	**<.0001** *	1	–	0.333	34	**<.0001** *	1	–	.495
	High	85	33		1.518	0.653–3.531		43		1.259	0.650–2.439	
miR‐429 expression	Low	194	48	**<.0001** *	1	–	0.352	66	**<.0001** *	1	–	.437
	High	101	4		0.584	0.188–1.813		11		0.751	0.364–1.547	
HK2 expression	Low	225	27	**<.0001** *	1	–	0.728	46	**<.0001** *	1	–	.180
	High	70	25		1.150	0.523–2.531		31		.660	0.359–1.212	

* Statistical significance.

**TABLE 4:** Univariate log‐rank and multivariate analyses for disease‐specific and metastasis‐free survival in upper urinary tract urothelial cancer (UTUC)

			**Disease‐specific survival**					**Metastasis‐free survival**				
			**Univariate analysis**		**Multivariate analysis**			**Univariate analysis**		**Multivariate analysis**		
**Parameter**	**Category**	**Case No**.	**No. of events**	***p*‐value**	**RR**	**95% CI**	***p*‐value**	**No. of events**	***p*‐value**	**RR**	**95% CI**	***p*‐value**
Sex	Male	158	28	.9301	–	–	–	32	.7904	–	–	–
	Female	182	33		–	–	–	38		–	–	–
Age (years)	<65	138	26	.8660	–	–	–	30	.8470	–	–	–
	≥65	202	35		–	–	–	40		–	–	–
Diabetes mellitus	Negative	276	45	.0867				52	.928			
	Positive	64	16					18				
Multifocality	Single	273	43	**.0042** *	1	–	**.003** *	52	**.0196** *	1	–	**.012** *
	Multifocal	62	18		2.474	1.361–4.495		18		2.075	1.175–3.665	
Primary tumor (T)	Ta	89	2	**<.0001** *	1	–	**.035** *	4	**<.0001** *	1	–	.299
	T1	92	9		2.976	0.598–14.808		15		2.536	0.779–8.254	
	T2–T4	159	50		4.993	1.082–23.040		51		3.322	1.006–10.976	
Nodal metastasis	Negative (N0)	312	42	**<.0001** *	1	–	**<.001** *	55	**<.0001** *	1	–	.260
	Positive (N1–N2)	28	19		3.211	1.666–6.191		15		1.446	0.761–2.744	
Histological grade	Low grade	56	4	**.0171** *	1	–	**.045** *	3	**.0019** *	1	–	.070
Vascular invasion	Absent	234	24	**<.0001** *	1	–	.104	26	**<.0001** *	1	–	**.001** *
	Present	106	37		1.653	0.902–3.030		44		2.787	1.516–5.125	
Perineural invasion	Absent	321	50	**<.0001** *	1	–	**.003** *	61	**<.0001** *	1	–	.109
	Present	19	11		3.208	1.475–6.978		9		1.897	0.867–4.152	
Mitotic rate (per 10 high power fields)	< 10	173	27	.1268	–	–	**–**	30	.0581	–	–	**–**
	≥10	167	34		–	–	**–**	40		–	–	**–**
CEBPD Amplification	Nonamplified	264	29	**<.0001** *	–	–	**–**	24	**<.0001** *	–	–	**–**
	Amplified	76	32		–	–	**–**	46		–	–	**–**
CEBPD expression	Low	251	26	**<.0001** *	1	–	.212	24	**<.0001** *	1	–	**.004** *
	High	89	35		1.758	0.725–4.260		46		3.506	1.484–8.282	
MYC amplification	Low	282	33	**<.0001** *	–	–	–	35	**<.0001** *	–	–	–
	High	58	28		–	–		35		–	–	
MYC expression	Low	265	23	**<.0001** *	1	–	**.013** *	27	**<.0001** *	1		**.013** *
	High	75	38		2.651	1.228–5.723		43		2.478	1.214–5.059	
miR‐429 expression	Low	257	55	**.0024** *	1	**–**	.996	64	**<.0001** *	1	–	.948
	High	83	6		0.997	0.386–2.579		6		0.970	0.392–2.401	
HK2 expression	Low	260	35	**<.0001** *	1	–	.142	34	**<.0001** *	1	–	.277
	High	80	26		0.580	0.280–1.200		36		0.688	0.351–1.350	

* Statistical significance.

### 3.5 CEBPD upregulation synergizes with glucose metabolism disorder to promote UC progression

**FIGURE 6:**
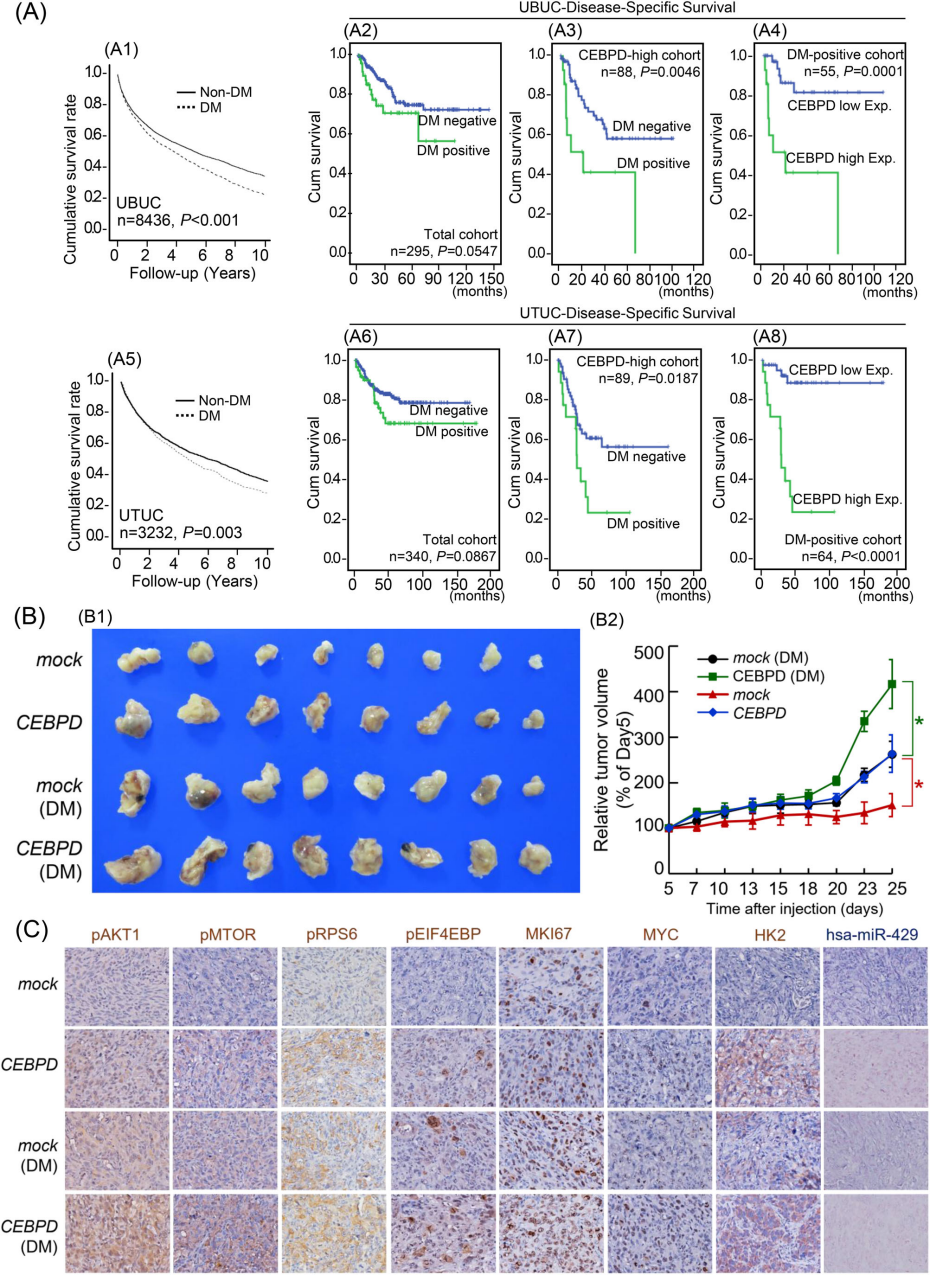
Diabetes mellitus (DM) exacerbates CCAAT/enhancer‐binding protein delta (CEBPD)‐driven tumour aggressiveness. (A) Analysis of the National Health Insurance Research Database (NHIRD) showed that urinary bladder urothelial carcinoma (UBUC) (A1, *n* = 8436, *p *< .001) and upper urinary tract urothelial cancer (UTUC) (A5, *n* = 3232, *p *= .003) patients with concomitant DM had a higher death rate than non‐DM patients. However, unlike the results for the NHIRD, which includes a large number of patients, concomitant DM only displayed marginal significance for the prediction of poor disease‐specific survival (DSS) in UBUC (A2, *n* = 295, *p *= .0547) and UTUC (A6, *n* = 340, *p *= .0867) in our cohort, which includes few cases, indicating that the significance of DM in patients’ outcome might be mild. Interestingly, for those cases with high CEBPD expression in our cohort, the presence of DM conferred a significantly worse prognosis in UBUC (A3, *n* = 88, *p *= .0046) and UTUC (A7, *n* = 89, *p *= .0187). Conversely, tumours harbouring high CEBPD expression were even more aggressive in DM patients in both the UBUC (A4, *n* = 55, *p *= .0001) and UTUC (A8, *n* = 64, *p *< .0001) cohorts. The aforementioned evidence revealed that CEBPD‐mediated aggressiveness in UC can be exacerbated by concomitant hyperglycemic disorder, a symptom that characterizes diabetes, probably because high‐glucose conditions help to reinforce CEBPD‐mediated glycolysis in cancer cells. (B) Experiments on a BFTC909‐derived xenograft model (*n* = 8 for each group) further reinforced that the effects of CEBPD on promoting tumour growth could be exacerbated by concomitant hyperglycemia. First, compared with the mock conditions, CEBPD overexpression promoted the growth of xenografted tumours in SCID/beige mice without high‐fat diet‐induced DM. Accelerated tumour growth driven by CEBPD overexpression was exacerbated in mice with high‐fat‐diet‐induced DM compared with those with CEBPD overexpression alone or induced DM alone (B1). The CEBPD‐induced and DM‐induced effects on tumour aggressiveness and the DM‐induced exacerbation of CEBPD‐driven tumour growth were all statistically significant (B2). (C) IHC analysis of xenografted samples at day 25 post‐injection showed that pAKT1, pMTOR, pRPS6, pEIF4EBP, MKI67, MYC and HK2 were highly expressed and that hsa‐miR‐429 were downregulated in the CEBPD‐overexpressing and/or DM‐induced groups compared to the control group. Of note, the upregulation of MYC and HK2 and downregulation of hsa‐miR‐429 were more prominent in the CEBPD‐overexpressing group than in the mock group, regardless of DM induction. This suggests that the CEBPD‐mediated effects on glycolysis might be regardless of hyperglycemia or normoglycemia conditions. For immunohistochemistry, one representative image was shown. Data are shown as the mean ± SEM. Statistical significance: * *p* < .05

Given that the aforementioned evidence revealed that positive feedback loops between CEBPD and MYC drive metabolic reprogramming toward glycolysis, we further evaluated whether DM, the most common cause of dysregulated glucose homeostasis and high glucose levels in peripheral tissue, is associated with a poor outcome among UC patients in nationwide cohorts. We confirmed the prognostic significance of glucose metabolism disorder in 8436 UBUC patients and 3232 UTUC patients from the NHIRD. DM patients had higher 5‐ and 10‐year mortality rates than non‐DM patients, with hazard ratios of 1.22 (5‐year survival for UBUC patients; 95% CI: 1.14–1.30, *p* < .001), 1.12 (5‐year survival for UTUC patients; 95% CI: 1.01–1.26, *p* = .044), 1.29 (10‐year survival for UBUC patients; 95% CI: 1.22–1.37, *p* < .001) and 1.17 (10‐year survival for UTUC patients; 95% CI: 1.06–1.29, *p* = .003), after adjusting for all comorbidities (Tables 7 and 8, Figure 6A1,5). Interestingly, the mortality risks of age, sex and comorbidities except COPD were irrelevant to the impact of DM status on UBUC and UTUC patients (Table S9), reinforcing DM as an independent prognostic factor in UC.

We then further explored whether DM is a survival determinant in UC and enhances CEBPD‐driven progression in our in‐house cohorts as well as a xenograft model. We found that the prognostic significance of concomitant DM only reached marginal significance in the prediction of inferior DSS in UBUC (*p* = .0547) and UTUC (*p* = .0867) in our cohort (Figure 6A2,6); the significance was probably less pronounced because our study included far fewer cases than the NHIRD. Nevertheless, concomitant DM was a significant predictor of worse outcomes in patients with high tumoral CEBPD expression (UBUC *p =* .0046; UTUC *p* = .0187) (Figure 6A3,7). and vice versa (UBUC *p =* .0001; UTUC *p <* .0001) (Figure 6A4,8).

Aside from clinical significance, deterioration of CEBPD‐driven tumour aggressiveness was also examined in an animal model with induced type 2 DM. In BFTC909‐xenografted SCID/beige mice without induced DM, the tumour size of the CEBPD‐overexpressing group was larger than that of the mock group (Figure 6B). Interestingly, the tumour growth advantage conferred by CEBPD overexpression was even more exacerbated in mice with high‐fat‐diet‐induced DM than in those with mock cell‐derived xenografts with or without DM and in those with CEBPD‐overexpressing cell‐derived xenografts without DM (Figure 6B). The tumour growth advantage was also supported by the upregulation of pAKT1, pMTOR, pRPS6, pEIF4EBP1, MKI67, MYC and HK2 and the downregulation of hsa‐miR‐429 (Figure 6C and Figure S9). Based on the above data, we disclosed DM is synergistically unfavourable to the survival outcome among patients with high expression of CEBPD by means of the aggressive tumour growth stemmed from the metabolic disorder of mTOR signalling and glycolysis.

## 4 DISCUSSION

In the present study, we consolidated the oncogenic features of CEBPD on UC through MAPK‐ and PI3K/AKT‐dependent mTORC1 axis which is the pivotal hub for cell growth. Furthermore, we noticed that *CEBPD* amplification is frequently accompanied by the amplification of *MYC*‐the ‘master regulator’ of glycolysis in our aCGH cohort. Accordingly, this co‐amplification confers more clinical aggressiveness which warrants further exploration of its biological significance. Moreover, we confirmed that CEBPD has a novel carcinogenic ability to impose metabolic reprogramming, facilitating the switch from mitochondrial OXPHOS to glycolysis, resulting in glucose addiction on UC. CEBPD‐induced high glucose demand was verified in SCID/beige mice with high‐fat‐diet‐induced DM. CEBPD accelerated tumour growth more potently in mice with DM than in those without DM. The findings mentioned above were further supported by significant correlations in our large clinical cohort and cohorts from the NHIRD. This CEBPD and MYC‐centric multilayered positive feedback loop enhances oncogenic glycolysis, which ensures cancer growth, especially during glucose metabolism dysregulation, indicating that it could be a target for combined treatment strategies. Albeit we clearly demonstrated the positive interplay of CEBPD and MYC on neoplastic progression, it is still worthwhile to excavate the potential oncogenic significance of CEBPD through the MAPK‐ and PI3K/AKT‐dependent mTORC1 survival signalling.

Clinically aggressive features are correlated with genetic alterations, especially copy number aberration in solid tumours, including UC.^41^ CIN distinguishes invasive UC from less aggressive papillary subtypes.^42^ We previously identified the frequent amplification of chromosomes 3q, 5p, 8q and 19q, as well as copy number losses of 2q, 4q, 5q, 6q, 9p, 9q, 11p, 11q, 13p 17p and 18q in muscle‐invasive UBUC.^23^ Of these changes, the CNG of chromosome 8q is highly recurrent in aggressive UC. Importantly, 8q24 manifests as the most dominant amplification core during UC progression^43^ and is one of the most significant copy number imbalances associated with metastasis development and a poor prognosis in UBUC.^10^ Amplification of *MYC* mapped to chromosome 8q24.21 is commonly observed in aggressive types of cancers and is strikingly related to a high risk of relapse and disease‐related death.^44^ MYC dysregulation induces genome instability such as CIN^18^, ^45^ may explain why *MYC* amplification is strongly linked to copy number aberrations of other oncogenes, such as *HER2* amplification in 40% of breast cancer and 5% of gastric cancer cases.^19^, ^46^ *HER2* was also detected to be coamplified with *MYC* (*p *< .01) in UC. The coamplification of *HER2* and *MYC* was also detected in 6% of UCs that were all in late‐stage.^47^ Moreover, overexpression of MYC has been reported to drive the amplification of *DHFR*, a critical enzyme in de novo purine and thymidylate biosynthesis, in human cell lines and preinvasive cervical cancer.^20^, ^48^ The current study is the first not only to reveal MYC expression induces an increased LOH in vitro as solid evidence of MYC‐induced CIN but also a positive correlation between the *MYC* and *CEBPD* gene dosages and expression levels in vitro and in vivo. Interestingly, an in vitro experiment showed that MYC overexpression induced *CEBPD* amplification but not vice versa. Moreover, *CEBPD* amplification was exclusively identified in patients with MYC gain, while the reverse pattern was not observed. These lines of evidence strongly support the hypothesis that *MYC* gain develops earlier than *CEBPD* gain and that MYC expression drives amplification‐driven CEBPD expression in UC. This is also supported by the fact that MYC amplification is more frequent than CEBPD amplification in UC and can even be observed in preinvasive high‐grade dysplasia, while CEBPD gain is less frequently found and is preferentially observed in an aggressive subset of UCs.^10^, ^49^

An earlier study reported that MYC overexpression in UC does not necessarily depend on its amplification^50^ and that the frequency of MYC overexpression surpasses that of *MYC* amplification in some tumour types, such as gastric adenocarcinoma.^51^ Similarly, a broad analysis of the TCGA database, which contains approximately 9000 specimens comprising 33 tumour types, showed that colon adenocarcinoma and rectum adenocarcinoma exhibit the highest *MYC* mRNA levels, but this upregulation is attributed to Wnt/β‐catenin signalling pathway activity instead of *MYC* amplification; chromophobe renal cell carcinoma (KICH) exhibits the highest MYC protein expression level but has a low *MYC* mRNA level and a low frequency of *MYC* amplification, suggesting that mRNA stability and translational or posttranslational regulation play roles in MYC protein expression levels.^52^ Similarly, *MYCN* (MYCN protooncogene, bHLH transcription factor) is amplified in approximately 20% of neuroblastomas (NBs) and is related to poor outcomes.^53^ Nevertheless, most NB tumours have substantial nuclear MYCN protein expression and relatively low MYCN mRNA levels, implying an alternative route for the protein stabilization of MYCN in this type of tumour.^54^ MYC and many other oncogenes, such as CCNE1, MTOR, JUN, NOTCH1 and AURKA, are targeted for ubiquitin‐proteasome‐mediated destruction by the vital tumour suppressor FBXW7, a component of the SCF E3 ligase complex.^39^ FBXW7 dysregulation is one means of tumorigenesis in various cancer types. Loss of function and low transcription of FBXW7 indicate unfavourable survival outcomes and are related to the aggression of muscle‐invasive BLCA due to MYC accumulation.^55^ CEBPD has been implicated in MTOR signalling enhancement by transcriptionally suppressing *FBXW7*, leading to mammary tumour metastasis.^56^ Our current study is the first to reveal that CEBPD exerts a protective effect by stabilizing MYC by transcriptionally repressing FBXW7 in UC. Although this study did not reveal a novel MYC regulatory mechanism, our results could explain why the MYC protein and mRNA expression patterns are often inconsistent in many cancers.

Dysregulated PI3K/AKT/mTOR pathway is one of the most important oncogenic signallings. In the present study, we also found that CEBPD activates the MAPK3/1 (ERK1/2) and PI3K/AKT/mTORC1 pathways in UC cell lines. MAPK3/1 (ERK1/2) plays a key role in tumour growth by regulating cell cycle progression.^57^ mTOR is a serine‐threonine kinase that forms two multiprotein complexes, namely, mTORC1 and mTORC2.^58^ Both mTORC1 and mTORC2 induce metabolic reprogramming by enforcing protein and lipid synthesis, aerobic glycolysis, and mitochondrial modulation to fuel tumour progression.^59^, ^60^ Of these pathways, aerobic glycolysis is a cancer hallmark for rapid energy production in the presence of oxygen to promote cell growth.^22^, ^61^, ^62^ mTORC1 promotes aerobic glycolysis by promoting the transcription of glycolysis‐related enzymes, including HK2, LDH A/C, pyruvate dehydrogenase kinase and glucose transporters, via hypoxia‐inducible factor 1 (HIF‐1) and MYC.^63^ mTORC2 governs glycolytic metabolism mainly through MYC and AKT.^63^ Apparently, dual regulation of MYC by the AKT/mTORC1 axis and mTORC2 has profound implications for glycolysis. The present study revealed that aerobic glycolysis is enhanced by CEBPD in UC cells by increased glucose uptake following increases in GLUT‐1 and HK2. Of note, we identified CEBPD promotes mitochondrial fission, which favours glycolysis over OXPHOS, supports the increase in ECAR and decrease in OCR in CEBPD‐overexpressing UC cells, reenforcing the glycolytic shift induced by CEBPD. Moreover, CEBPD strengthens glycolytic flux at least partly through MYC regulation, as verified by the partial loss in GLUT‐1 and HK2 expression after siMYC treatment in CEBPD‐overexpressing cells. For the first time, our findings disclosed a novel insight regarding oncogenic characters of CEBPD as an enhancer of glycolysis.

HK2 belongs to the hexokinase family, which is composed of four isoforms (HK1, HK2, HK3 and HK4).^64^ HK2 is a crucial glycolytic enzyme that converts glucose to G6P in the first step of glycolysis^64^ and functions as an oncogene. It has been found to be highly upregulated in cancers and to be associated with poor clinical outcomes.^65^, ^66^ Numerous miRNAs have been found to regulate various metabolic pathways directly by targeting these key enzymes or indirectly by impacting the levels of transcription factors.^67^ Interestingly, various miRNAs participate in glycolytic reprogramming by targeting glucose transporters and glycolytic enzymes in tumours.^68^ However, few previous reports have revealed the role of miRNAs in the regulation of HK2 expression in UC. To date, only hsa‐miR‐125b‐5p has been reported to directly target HK2 to suppress the progression of BLCA.^69^ Our report expanded the spectrum of miRNAs that regulate glycolysis in UC by highlighting the role of miR‐429, where direct transcriptional repression of hsa‐miR‐429 by CEBPD promotes HK2 expression. miR‐429 belongs to the miR‐200 family (i.e., miR‐200a, miR‐200b, miR‐200c, miR‐141 and miR‐429) and functions as a tumour suppressor, tumour promoter or both depending on the tumour type and expression pattern.^70^ miR‐429 has previously been shown to have a role in BLCA by suppressing the migration and invasion of a BLCA cell line by targeting zinc finger E‐box binding homeobox 1 and β‐catenin, both of which are related to epithelial‐mesenchymal transition.^71^ A computational algorithm further revealed an additional role of miR‐429 as a potential negative modulator of anaerobic glycolysis by affecting the gene level of HIF‐1α.^67^ Finally, miR‐429 may serve as a prospective biomarker of clinicopathological traits and prognostic outcomes.^72^ We expanded these known roles in the present study, where we revealed that miR‐429 plays a crucial role in the CEBPD‐induced promotion of glycolysis.

In conclusion, we unearthed that the CIN of MYC drives CEBPD amplification and that the upregulation of CEBPD plays a protective role by stabilizing the MYC protein through the transcriptional suppression of FBXW7 in UC‐derived cell lines. Cell proliferation, glucose addiction, and switching from mitochondrial fusion to fission are promoted as a result of mTOR signalling hyperactivation and MYC‐dependent glycolysis dysregulation induced by CEBPD overexpression. CEBPD also promotes glycolysis by inhibiting hsa‐miR‐429, thus upregulating *HK2* in an MYC‐independent manner. Overall, we are the first to show that glycolysis is promoted via the CEBPD/FBXW7/MYC positive feedback loop and the novel CEBPD/hsa‐miR‐429/HK2 axis. Taken together, our data indicate a novel role of CEBPD in metabolic reprogramming. More importantly, given that directly targeting MYC has been proved difficult, our study disclosed a possible combined treatment strategy targeting both MYC and CEBPD to more effectively prohibit MYC‐initiated tumorigenesis.

## CONFLICT OF INTEREST

The authors declare that they have no conflict of interest.

## Supporting information



Supporting Information
