# Supplementary material for: Biological significance of MYC and CEBPD coamplification in urothelial carcinoma: Multilayered genomic, transcriptional and posttranscriptional positive feedback loops enhance oncogenic glycolysis
Source: Clin Transl Med. 2021 Dec 26;11(12):e674. doi: 10.1002/ctm2.674 (PMC8710299; doi:10.1002/ctm2.674)
Supplement: Supplementary file 1 — Supporting Information [file CTM2-11-e674-s001.docx]

**Biological significance of** ***MYC* and *CEBPD* coamplification in urothelial carcinoma: multilayered genomic, transcriptional, and posttranscriptional positive feedback loops enhance oncogenic glycolysis**

**Running title:** *MYC* and *CEBPD* coamplification in urothelial carcinoma

Ti-Chun Chan^1,2^, Yi-Ting Chen^3^, Kien Thiam Tan^4^, Chia-Ling Wu^4^, Wen-Jeng Wu^5,6,7^, Wei-Ming Li^5,6,7^, Ju-Ming Wang^3^, Yow-Ling Shiue^8^, Chien-Feng Li^1,2,8,*^

**Supplementary Materials**

**Materials and Methods**

**Tumor tissue sample sets**

In this study, we performed immunohistochemistry (IHC) and chromogenic in situ hybridization (CISH) of well-established sample sets consisting of 295 UBUC and 340 UTUC samples from the biobank of Chi Mei Medical Center. The archived tumor samples were collected after surgery with curative intent between January 1996 and May 2004 as previously described ^22^. A total of 32 snap frozen UC samples with paired non-tumor urothelium were evaluated for *CEBPD* mRNA and protein expression. This study was approved by the institutional review board of Chi Mei Medical Center (IRB10207-001).

**Reanalysis of array comparative genomic hybridization data**

Reanalysis of our published aCGH dataset containing 40 UBUC samples ^10^ was performed by using Nexus Copy Number™ software (BioDiscovery, USA) as previously described ^23^ to profile the status of *CEBPD* and *MYC* coamplification. For this analysis, the copy-number gain was defined as log2 ratio >0.2

**Data mining**

We deciphered the correlation between the *CEBPD* and *MYC* gene dosages in The Cancer Genome Atlas (TCGA)-bladder cancer (BLCA) dataset and the association between the *CEBPD* and *HK2* gene expression in the GSE13507 dataset through the Oncomine^TM^ platform (Research Premium Edition).

**Cell culture**

To systemically evaluate the crosstalk between CEBPD and MYC, four human UC cell lines including RT4 (American Type Tissue Culture Collection [ATCC], VA), HT1197 (ATCC, VA), TCCSUP (ATCC, VA) and BFTC909 (Food Industry Research and Development Institute, Taiwan) were screened for the level of *CEBPD* and *MYC* expression. BFTC909 and TCCSUP which showed relatively low endogenous *CEBPD* and *MYC* expression were selected for the study. RT4 was incubated in McCoy’s 5A medium with 10% FBS and 1% penicillin/streptomycin (P/S). HT1197 was maintained in Minimum Essential Media (MEM) with 10% FBS, 1% Non-Essential Amino Acid (NEAA) and 1X Antibiotic-Antimycotic (100X). BFTC909 and TCCSUP were

cultured in Dulbecco's Modified Eagle Medium (DMEM) supplemented with 10% FBS and 1% P/S. All cells were incubated in a humidified incubator containing 5% CO_2_ at 37°C. The cell culture media, fetal bovine serum and antibiotics were all purchased from Gibco.

**Exogenous** **gene overexpression in cell lines**

For the preparation of viral supernatant, Phoenix-AMPHO cells (ATCC, USA) incubated in

DMEM supplemented with 10% FBS, 1% glutamine and 1% P/S were cotransfected

with lentiviral vector contained gene of interest, lentiviral packaging plasmids (psPAX2) and lentiviral envelope plasmid (pMD2.G) dissolved in the Opti-MEM® I Reduced-Serum Medium (Gibco) through PolyJet™ transfection reagents (SignaGen® Laboratories). The cell culture medium contained transfection mixture was replaced with fresh culture medium at 16 hours posttransfection. The viral supernatant was captured by a 0.22-μΜ PES filter (JET Biofil) after purification. BFTC909 and TCCSUP were incubated with culture medium containing 10 µg/mL polybrene and viral supernatant for 24 hours. Afterward, medium containing viral solution was replaced with fresh medium. Following another 24 hours, the cells were incubated with culture medium containing 2 µg/mL puromycin for 72 hours to proceed the selection process and were further maintained in a lower concentration of puromycin (1µg/mL) for further experiments.

**Plasmids and oligomers**

pLKO-AS3w-eGFP, pLKO-AS3w-*CEBPD*-eGFP ^10^, P-Lenti-*c-Myc* -DDK (RC201611L1, Origene) were utilized for the generation of stable mock-expressing, CEBPD-overexpressing, MYC-overexpressing cell lines, respectively. The pLV-mitoDsRed vector (44386, Addgene, USA) embracing a mitochondrial targeting sequence fused to a recombinant red fluorescent tag (dsRed) was used to detect mitochondrial fission and fusion events. SignalSilence® *c-Myc* siRNAs (6341, 6552; Cell Signaling Technology, USA) were used for the knockdown of MYC expression. Analysis of promoter activity was conducted by using the following reporters: The F-box and WD repeat domain-containing 7 (*FBXW7*) promoter (S700873) was ordered from Active Motif. The hsa-miR-429-WT promoter reporter, hsa-miR-429-mutant promoter reporter, hsa-miR-429 mimic, and hsa-miR-429 inhibitor were constructed by TopGen (Taiwan). pKM2L-ph*HKII* (RDB05882) was ordered from Riken (Japan). The pGL4.54[luc2/TK] vector (E5061) was purchased from Promega (USA) served as a control vector. WT-*HK2*-3’-untranslated region (UTR) and mutant-*HK2*-3’-UTR cloned into the pMIR-REPORT Luciferase miRNA Expression Reporter Vector contains firefly luciferase (Topgen Biotechnology, Taiwan) were used for the *HK2*-3’-UTR -reporter assay.

**MicroRNA (miRNA) sequencing**

We performed NGS on both BFTC909 and TCCSUP cells harboring mock or CEBPD expression plasmids. Total RNA was extracted by TRI Reagent® RNA Isolation Reagent (Sigma, USA). Then, 1 μg of high-quality total RNA was used to construct a small RNA library with a TruSeq Small RNA Sample Preparation Kit (Illumina, US) according to its manual instructions. Briefly, total RNA was ligated to the 3’ adaptor and 5’ adaptor sequentially, followed by reverse transcription PCR. A proper cycle of PCR were performed to add the index sequences and finish the cDNA library construction, which was followed by gel purification. The constructed libraries were examined for their quality and quantity via gel electrophoresis, Qubit and qPCR measurement. The libraries that passed the quality check were diluted to the proper concentration and sequenced on an Illumina NextSeq sequencer (Illumina, San Diego, CA).

After sequencing, the FASTX-Toolkit (http://hannonlab.cshl.edu/fastx_toolkit) was used for the quality control process. After alignment, the information obtained was profiled by using miRBase v21. An expectation-maximization (EM) algorithm was used to normalize miRNA expression data. Finally, the expression level of each miRNA was divided by the total number of aligned reads for precise quantification. The miRNAs downregulated in the CEBPD-expressing group with a fold change no less than log2 ratio -1 in at least one cell and log2 ratio exceeding -0.3 in another cell with a significance of *P*<0.05 in duplication tests were selected for further analysis to ensure the significance of the miRNA candidates.

**Real-time quantitative RT-PCR to quantify the mRNA and hsa-miRNA levels**

A Quick-RNA™ Miniprep Kit (Zymo Research, CA, USA) was utilized to extract total RNA (including miRNAs) from the cell lines according to the manufacturer’s instructions. Briefly, the cell pellet was lysed by RNA lysis buffer. The lysis suspension was transferred to a Spin-Away^TM^ Filter with a collection tube. After centrifugation at 15,000 × g for 1 min, the filtered solution was mixed with 100% ethanol and transferred to a Zymo-Spin™ IIICG Column with a collection tube. After centrifugation, the Zymo-Spin™ IIICG Column was washed with RNA wash buffer and incubated with DNase I dissolved in DNA digestion buffer. After that, the Zymo-Spin™ IIICG Column was washed with RNA prep buffer once and RNA wash buffer twice. The RNA was eluted with DNase/RNase-free water through high-speed centrifugation. For tissue RNA preparation, the sample was disrupted and lyzed by ZR BashingBead Lysis Tubes (Zymo) with RNA lysis buffer in vortexer at room temperature for 30 minutes before standard RNA extraction procedure.

For evaluation of the mRNA levels, purified RNA was subjected to cDNA synthesis using the Maxima First Strand cDNA Synthesis Kit (Thermo Scientific). Briefly, a final volume of 20 μL containing 1 μg of total RNA, 4 µL of the reaction mix, 2 µL of Maxima Enzyme Mix and nuclease-free water was incubated at 25°C for 10 min, followed by another incubation at 50°C for 30 min and a final incubation at 85°C for 5 min. Then, the cDNA was mixed with a predesigned TaqMan assay mixture (probe and primer set: *CEBPD* [Hs00270931_m1], *MYC* [Hs00153408_m1], *FBXW7* [Hs00217794_m1] and *HK2* [Hs01034055_g1]; Applied Biosystems) and TaqMan Fast Advanced Master Mix (Applied Biosystems), and then, the final volume was adjusted to 20 µL with distilled water. The mixture was subjected to quantitative RT-PCR to measure the mRNA level via a StepOne Plus System (Applied Biosystems). The thermal cycling conditions were 20 sec at 95 ℃, followed by 40 cycles of 95 ℃ for 1 sec and 60 ℃ for 20 sec. The cycle threshold (Ct) value of the target gene was normalized to that of the reference gene POLR2A, which served as the ∆Ct value. The relative mRNA expression of the target gene to the control gene was estimated using equation 2^-∆∆CT^.

For the determination of miRNA expression, a TaqMan™ Advanced miRNA cDNA Synthesis Kit (Thermo Scientific) was utilized to prepare the cDNA template of miRNA. Briefly, a final volume of 15 μL consisting of an RT primer pool (TaqMan miRNA assay: hsa-miR-429 and snRNA U6), RTase, 10X RT buffer, dNTP with dTTP, RNase inhibitor and nuclease-free water was subjected to the following thermal cycling conditions: 16°C for 30 min, followed by 30 min at 42°C and finally 85°C for 5 min. The cDNA was mixed with a predesigned TaqMan assay mixture (probe and primer set targeting hsa-miR-429, U6 snRNA; Topgen Biotechnology) and TaqMan Fast Advanced Master Mix (Applied Biosystems), and the final volume was adjusted to 10 µL with distilled water. The thermal cycling conditions were 20 sec at 95°C, followed by 40 cycles of 95°C for 1 sec and 60°C for 20 sec. The relative miRNA level was calculated by the 2^-∆∆CT^ formula, and snRNA U6 was used as a reference gene.

**Cell viability and cell proliferation assays**

2,3-Bis-(2-methoxy-4-nitro-5-sulfophenyl) 2H-tetrazolium-5-carboxanilide (XTT) was used to evaluate cell viability. Briefly, 1000 cells were seeded in 96-well microplates and incubated for 24, 48 and 72 hours at 37°C. After that, 150 µL of a mixture of XTT and PMS diluted in RPMI 1640 (no phenol red) with a final concentration of 0.3 mg/mL and 20 µM, respectively, was added to each well. One hour after treatment with the XTT/PMS solution, the absorbance was measured at 450 nm with a reference wavelength at 600 nm in an ELISA reader (GM3000, Promega).

Cell proliferation was evaluated by a Cell Proliferation Assay Kit (Fluorometric, Biovision). Briefly, 1000 cells were seeded in 96-well microplates and incubated for 24, 48 and 72 hours at 37°C. Then, 25 μL of reaction mixture including 1X Nuclear Dye/Cell Lysis Buffer solution and 1X Nuclear Dye was added to each well. After incubation for 15 min, the fluorescence intensity was detected by a microplate reader at excitation/emission wavelengths of 480/538 nm.

**Western blot assays**

For tissue protein extraction, the sample was disrupted and lyzed by ZR BashingBead Lysis Tubes (Zymo) with PRO-PREP Protein Extraction Solution (iNtRON) in Vortexer at 4°C for one hour. For cell protein isolation, the total protein was directly extracted by PRO-PREP Protein Extraction Solution (iNtRON) on ice for 30 minutes. Thirty micrograms of protein were separated on a NuPAGE Bis-Tris Gel (Invitrogen) and transferred onto an Immobilon-P PVDF membrane (Millipore). The membrane was blocked with 5% skim milk (Sigma) and probed overnight with the indicated primary antibody. Following the incubation with the appropriate secondary antibody (HRP Donkey anti-rabbit IgG [BioLegend®, USA, 406401] or HRP goat anti-mouse IgG H&L [Abcam, ab97023]) diluted in 5% skim milk, the protein of interest was visualized with ECL (Thermo Scientific). Primary antibodies against the following proteins were used: phospho-MAPK3/1 (Erk1 [pT202/pY204] + Erk2 [pT185/pY187], Abcam, ab50011, 1:1000), MAPK3/1 (Erk1/2) (Cell Signaling, 4695S, 1:1000), phospho-PI3K (Tyr 607, ab182651, 1:1000), PI3K (4292S, 1:1000), phospho-AKT (Ser473, Cell Signaling, 4060, 1:1000), AKT (Cell Signaling, 4691, 1:1000), phospho-MTOR (Ser2448, Abcam, ab51044, 1:500), MTOR (Abcam, ab32028, 1:500), phospho-RPS6 (Ser235, Abcam, ab80158, 1:10000), RPS6 (Abcam, ab137826, 1:1000), phospho-4E-BP1 (Ser65, Cell Signaling, 9451, 1:500), 4E-BP1 (Cell Signaling, 9644, 1:500), caspase-3 (active) (EPITOMICS, 1476-1, 1:500), FBXW7 (Abcam, ab105752, 1:1000), SKP2 (Abcam, ab124799, 1:1000), MYC (Abcam, ab32072, 1:1000), SLC2A1 (Cell Signaling, ab105752, 1:1000), HK2 (Cell Signaling, 2867, 1:1000), and GAPDH (Abcam, AB181602).

**Flow cytometric analysis**

For cell cycle analysis, harvested cells were washed by PBS three times and were treated with cold 70% of ethanol for fixation at -20°C overnight. After ethanol removal through centrifugation at 300 x g for 5 min, the cell pellet was washed with PBS three times and resuspended in propidium iodide (PI)/ribonuclease (RNase) staining buffer (550825; BD Biosciences, USA) protected from light for 15 min at room temperature. The cell cycle distribution was examined by using a Novocyte™ flow cytometer (ACEA Biosciences, USA).

PI and APC Annexin V were used for apoptosis assay (51-66211E, 559925; BD Biosciences).

Fluorescence compensation was conducted using unstained cells and single-stained cells with treatment of paraformaldehyde and applied to the following apoptosis experiments. Live cells were washed with cold PBS twice and resuspended in 1X Binding Buffer. Each sample was treated with 5 μL of PI and 5 μL of APC Annexin V and incubated for 15min in the dark. The number of apoptotic cells was analyzed by Novocyte™ flow cytometer.

**Glucose uptake assays**

Glucose uptake was evaluated by a Glucose Uptake Assay Kit (Colorimetric, Abcam). A total of 2 x 10^4^ cells were seeded in a 96-well culture plate. After glucose starvation, the cells were treated with Krebs-Ringer-Phosphate-HEPES (KRPH) buffer supplemented with 2% BSA and 1 µM insulin and incubated for 20 min in an incubator at 37°C. 2-Deoxyglucose (2-DG) was added to each well, and the cells were incubated for 20 min at 37°C. After washing with DPBS, the cells were lysed with extraction buffer and frozen/thawed at 20°C once. After that, the samples were heated at 85°C for 40 min and chilled once for 5 min. After 10 µL Neutronization buffer was added, the samples diluted in assay buffer (1:10) were mixed with reaction mix A, which contained assay buffer and enzyme mix, for 60 min at 37°C. The samples were then mixed with extraction buffer and incubated at 90°C for 40 min. After cooling and neutralization with neutralization buffer, Reaction Mix B, which comprised glutathione reductase, substrate and recycling mix, was added to each sample and incubated at 37°C for 15 min. The absorbances were measured at a wavelength of 405 nm with an ELISA reader (GM3000, Promega).

**Lactate level analysis**

Lactate production was assessed using an L-lactate assay kit (Colorimetric, Abcam). Briefly, 2 x 10^6^ cells were harvested and suspended in 200 µL of lactate assay buffer. After thorough mixing, the supernatant was collected following high-speed centrifugation at 4°C. Endogenous LDH was removed using the Deproteinizing Sample Preparation Kit (Abcam). Fifty microliters of each sample were mixed with 50 µL of reaction mix composed of lactate assay buffer, lactate substrate mix and lactate enzyme mix in a 96-well plate and incubated for 30 min. The absorbances were evaluated at a wavelength of 450 nm with an ELISA reader (GM3000, Promega).

**Extracellular acidification rate (ECAR) and oxygen consumption rate** **(OCR) assays**

The ECAR and OCR were assessed by an XFp analyzer (Seahorse Bioscience; USA) according to its manufacturer’s instructions. Briefly, 1 x 10^4^ of cells were seeded in an 8-well cell culture miniplate for 24 hours. Seahorse XF Calibrant Solution was loaded into each well of the sensor cartridge to hydrate the probe tip and incubated in a non-CO_2_ incubator for at least 4 hours before the experiment. For the ECAR assay, 10X stocks of glucose, 2-DG, and oligomycin were dropped into the upper pores of the cartridge. Each stock solution was injected into the lower part of the cartridge during the detection procedure, and the final concentrations of glucose, oligomycin, and 2-DG were 100 mM, 50 μΜ and 500 mM, respectively. Afterward, the sensor cartridge was put in the Seahorse XFp Extracellular Flux Analyzer for calibration and equilibration. Meanwhile, the cells in the miniplate underwent glucose starvation with Seahorse XF base medium for 30 min in a non-CO_2_ incubator. After that, the lower plate of the sensor cartridge inside the XFp Extracellular Flux Analyzer was replaced with a cell-seeding miniplate to initialize the measurement of the ECAR.

For the OCR assay, a 10X stock of oligomycin, carbonyl cyanide-p-trifluoromethoxyphenylhydrazone (FCCP), and rotenone/antimycin A were added sequentially into the proper pores of the sensor cartridge at final concentrations of 10 μΜ, 5 μΜ and 5 μΜ, respectively, after injection. After calibration and equilibration, the culture miniplate with cells incubated in the Seahorse XF base medium containing glucose was substituted for the lower plate of the sensor cartridge, and the measurement was performed. The ECAR and OCR data were analyzed with Agilent Seahorse Wave Desktop software.

**Luciferase reporter assays**

The *FBXW7* promoter reporter (optimized Renilla luciferase, Active Motive) or pGL4.54[luc2/TK] vector (firefly luciferase, Promega) was transfected into 1 x 10^4^ of cells seeded in 96-well culture plates using PolyJet™ In Vitro DNA Transfection Reagent (Signagen) according to its protocol. After 18 hours posttransfection, the transfect medium was replaced with a fresh culture medium. Optimized Renilla luminescence intensity was evaluated 66 hours posttransfection with the LightSwitch™ Luciferase Assay Kit (Active Motive). Briefly, assay solution containing assay buffer and assay substrate equal to the volume of cell culture medium was directly added into each well and incubated for 30 min before assessment with an ELISA reader (GM3000, Promega). A mixture including ONE-Glo® Luciferase Reagent and enzyme (ONE-Glo™ Luciferase Assay System, Promega) was added to each well at a volume equal to the culture medium for 30 min and firefly luminescence signal was detected by an ELISA reader (GM3000, Promega). Finally, the Renilla luminescence value normalized by the firefly luminescence value was used to indicate the *FBXW7* promoter activity.

The hsa-miR-429 promoter sequence (HPRM 22169-PG04; Genecopoeia) was cloned into a dual-reporter backbone encompassing the Gaussia luciferase (Gluc) reporter, and the secreted alkaline phosphatase (SEAP) reporter served as an internal control. The hsa-miR-429 promoter plasmid was transfected into cells by PolyJet™ In Vitro DNA Transfection Reagent, and the luminescence light was estimated by Secrete-Pair™ Dual Luminescence Assay (Genecopoeia). Briefly, 10 µL of culture medium was mixed with 100 µL of Gluc Assay Working Solution composed of substrate GL and 1X Buffer GL-S in a 96-well white plate. After incubation for 1 min at RT, the Gaussia luminescence signal was measured by an ELISA plate reader (GM3000, Promega). To assess the SEAP signal, the culture medium was heated at 65°C for 15 min. After cooling, 10 µL of the heated medium was mixed with SEAP Assay Working SolutEion in a 96-well white plate and incubated for 10 min at RT, followed by the assessment of light intensity. The GLuc activity (GLuc/SEAP ratio) was calculated for all samples.

WT-*HK2*-3’-untranslated region (UTR) and mutant-*HK2*-3’-UTR cloned into the

pMIR-REPORT Luciferase miRNA Expression Reporter Vector contains a firefly luciferase

were cotransfected with pGL4.74[hRluc/TK] into cells, respectively, by using PolyJet™ In Vitro DNA Transfection Reagent. The culture medium was replaced with fresh medium 8 hours posttransfection. After that, cells were treated with hsa-miR-429 mimic or control mimic by using jetPRIME® transfection reagent (Polyplus). The culture medium was replaced with fresh medium 8 hours posttransfection. After the incubation for two days, the luminescence signal was measured by a Dual-Glo® Luciferase Assay System (Promega) as described below: reaction buffer composed of Dual-Glo® Luciferase Reagent and enzyme was added to each well at a volume equal to the culture medium (100 µL), and firefly luminescence was read by ELISA reader (GM3000, Promega) after incubation for 30 min. After that, 100 µL of Dual-Glo® Stop & Glo® Reagent was added to each well and incubated for 10 min to measure the activity of Renilla luciferase by ELISA reader. Finally, the firefly luminescence units were normalized by the Renilla luminescence units to reflect the activity of each *HK2*-3’-UTR reporter.

Mock- and CEBPD-expressing cells were cotransfected with pKM2L-ph*HKII* (Renilla luciferase, Riken) and pGL4.54[luc2/TK] vectors using PolyJet™ In Vitro DNA Transfection Reagent. Moreover, hsa-miR-429 mimic or control mimic was delivered into these cells using jetPRIME® transfection reagent. Fresh culture medium was substituted for the transfection medium 8 hours posttransfection. Two days posttransfection, the luminescence signal was measured by a Dual-Glo® Luciferase Assay System (Promega) as described above. Finally, the Renilla luminescence units were normalized to the firefly luminescence units to indicate *HK2* promoter activity. These data are represented as the relative luminescence units of the CEBPD-expressing group over those of the mock-expressing group.

**Analysis of copy number variation by qPCR**

Genomic DNA was extracted using the QIAamp DNA Mini Kit (Qiagen) according to the manufacturer’s instructions. Briefly, cell pellets were resuspended in 200 μL of DPBS. Twenty microliters of proteinase K and 200 μL of Buffer AL were added to the sample and incubated at 56°C for 10 min. Then, 200 μL of ethanol (100%) was added to the mixture to precipitate genomic DNA. The sample was added to a column with a collection tube and centrifuged at 15,000 × g for 1 min. After removing the waste, the column was washed with AW1 and centrifuged at 15,000 × g for 1 min again. Following discarding the waste, the column was washed again with AW2 and centrifuged at 15,000 × g for 1 min. After discarding the waste, the column was transferred to a new collection tube and centrifuged at the highest speed for 2 min to remove the remaining solution. Appropriate nuclease-free water was added to the central membrane of the column and centrifuged at 15,000 × g for 1 min to collect the purified genomic DNA. The genomic DNA was mixed with TaqMan® SNP Genotyping Assay probes (PIK3R1, Hs06028467_cn; CEBPD, Hs01845884_cn; Hs01879749_cn; Hs02239340_cn) and TaqMan® Genotyping Master Mix (Applied Biosystems), and then the final volume was adjusted to 20 µL with distilled water. The mixture was subjected to quantitative RT-PCR performed with the StepOne Plus System (Applied Biosystems). The thermal cycling conditions were as follows: 10 min at 95 ℃, followed by 40 cycles of 95 ℃ for 15 sec and 60 ℃ for 1 min. The PIK3R1 gene was used as an internal control. The Ct value of the target gene was normalized to that of the reference gene (PIK3R1), and this value was defined as ∆Ct. Relative quantification (RQ) was performed by using the 2^-ΔΔCt^ formula, and the copy number was represented as 2×RQ.

**Next-Generation Sequencing (NGS) based Loss of Heterozygosity (LOH) Analysis**

To further elucidate whether MYC expression enhances genomic instability, which is assumably the causal factor of *CEBPD* amplification, we use NGS-based ACTHRD^TM^ panel (ACT Genomics) to measure the loss of heterozygosity (LOH) in cell lines. For this assay, genomic DNA extracted from cells with or without MYC overexpression was amplified using primer pairs targeting coding exons of analyzed 24 homologous recombination repair (HRR)-related genes (Supplementary Table 1) and 8,833 single nucleotide polymorphisms (SNPs) evenly distributed across the entire genome. Amplicons were enriched and ligated with barcoded adaptors via the indexing PCR amplification with CleanPlex Indexed PCR Primers (Paragon Genomics). Barcoded libraries were subsequently purified using the CleanPlex Targeted Library Kit (Paragon Genomics). The amplified library's quality and quantity were determined using the fragment analyzer (AATI) and Qubit (Invitrogen). Sequencing was performed on the NextSeq 550 sequencer using the High-output flow cell and the NextSeq 500/550 High Output Kit v2.5 (Illumina) according to the manufacturer’s instructions.

Raw reads generated by the sequencer were mapped to the hg19 reference genome using BWA to determine SNPs. Coverage depth was calculated using Bedtools and Samtools. Single nucleotide variants (SNVs) and short insertions/deletions (INDELs) were identified using Pisces. VEP (Variant Effect Predictor) was used to annotate every variant using databases from Clinvar and Genome Aggregation Database. Variants with coverage ≥ 15, variant read counts ≥ 10, and allele frequency ≥ 5% were considered mutations and retained.

Mutations were categorized into five tiers (Deleterious, likely deleterious, variant of unknown significance (VUS), likely benign and benign) according to the standards and guidelines for cancer sequence variants interpretation by the Association for Molecular Pathology, American Society of Clinical Oncology, and College of American Pathologists.

For CNV analysis, amplicons with reading counts in the lowest 1st percentile or the highest 0.2% of all detectable amplicons and amplicons with a coefficient of variation ≥ 0.6 were removed. The remaining amplicons were normalized to correct the pool design bias. ONCOCNV (an established method for calculating copy number aberrations in amplicon sequencing data by Boeva et al., 2014) was applied for the normalization of total amplicon number, amplicon GC content, amplicon length, and technology-related biases, followed by segmenting the sample with a gene-aware model.

**Chromatin immunoprecipitation (ChIP)**

A SimpleChIP® Enzymatic Chromatin IP Kit (Cell Signaling) was used in this study. Briefly, the cells were treated with 1% paraformaldehyde for 10 min and incubated with 1X glycine solution to quench the formaldehyde. After washing with cold PBS containing PMSF, the cell pellets were suspended in 1X Buffer A supplemented with DTT and Protease Inhibitor Cocktail (PIC), incubated for 10 min on ice, and vortexed for 3 min. After centrifugation, the supernatant was discarded. The pellet was resuspended in ice-cold 1X Buffer B with DTT and centrifuged again to remove the supernatant. The pellet was treated with 1X Buffer B with DTT containing micrococcal nuclease at 37°C for 20 min to digest chromatin DNA. After quenching the digestive reaction with EDTA, the pellet was suspended in 1X ChIP Buffer with PIC and incubated on ice for 10 min. The nuclear membrane was broken down by sonication, and fragmented cross-linked chromatin was captured after centrifugation. Fragmented chromatin was incubated with a primary antibody against CEBPD (Santa Cruz, sc-365546) at 4°C with rotation overnight. After that, the mixture was incubated with protein G magnetic beads for 2 hours at 4°C with rotation. Afterward, the complex of precipitates and protein G beads were rinsed with low-salt buffer three times and high-salt buffer once for 5 min per step. The precipitated DNA was purified from the antibody/protein G complex and subjected to quantitative RT-PCR. The thermal cycling conditions were as follows: 3 min at 95 ℃, followed by 40 cycles of 95 ℃ for 15 sec and 60 ℃ for 1 min. The primers used to target the CEBPD-responsive element binding site on the *hsa-miR-429* promoter were as follows: forward: 5'-GGTTCTTCCCTGGGCTTC-3'; reverse: 5'-AGTGTTAGAGTCAAGCTGGGAAAT-3'.

**IHC**

Formalin-fixed, paraffin-embedded (FFPE) blocks were sliced into 4-μm thick slices and transferred onto silane-coated slides. The slides were placed in a 65°C oven for 20 min to melt the paraffin. For deparaffinization and rehydration, the sections were sequentially soaked in xylene, 100% ethanol, 95% ethanol and 75% ethanol for 5 min each and were then incubated in distilled water for 5 min. Next, the sections were immersed in citrate buffer (pH 6) and boiled in a microwave oven for 20 min for antigen retrieval. After cooling at room temperature, the sections were washed with PBS three times and incubated with peroxidase-blocking solution (Dako) to quench endogenous peroxidase activity. After washing with PBS, the sections were incubated with the primary antibody against the target protein for one hour at room temperature. Primary antibodies against the following proteins were used: MYC (Abcam, ab32072), CEBPD (Abcam, ab184911), HK2 (Cell Signaling, 2867), FBXW7 (Abcam, ab105752), phospho-AKT (Cell Signaling, 4060), phospho-mTOR (Abcam, ab51044), phospho-4E-BP1 (Cell Signaling, 9644), phospho-RPS6 (Abcam, ab80158), and MKI67 (Abcam, ab66155). And then with a secondary antibody (REAL™ EnVision™/HRP, rabbit/mouse (ENV), Dako) for 30 min at RT. Staining was visualized with EAL™ DAB+ Chromogen diluted in REAL™ Substrate Buffer (Dako). Hematoxylin was used for the nuclear stain. Finally, the slices were dehydrated by soaking in 75%, 95% and 100% ethanol for 5 min and mounted in UltraCruz® Aqueous Mounting Medium with DAPI. The IHC staining results were examined with an optical microscope and quantified into H-scores by three expert pathologists (CFL, TJC & WSL) as previously mentioned ^22^.

**CISH**

The human *MYC* gene was detected by a CISH assay. Briefly, FFPE slides were placed in a 65°C oven for 20 min. After that, the slices were deparaffinized and rehydrated by soaking in xylene and ethanol (100%, 95% and 75%) for 5 min each. Then, the slices were soaked in 3% H_2_O_2_ for 5 min. After washing with distilled water and air drying, the slices were immersed in 0.2 N HCl for 20 min with shaking and then washed three times with distilled water. Next, the slices were soaked in pretreatment buffer (1 N NaSCN in Tris-HCl, pH 8) for 30 min in an 80°C water bath. After washing with distilled water and saline-sodium citrate (SSC) buffer, pepsin dissolved in protease buffer (0.9% NaCl, pH 1.5) was used to treat slices for 20 min in a 37°C water bath. Following washing with SSC buffer, the slices were covered with 4% paraformaldehyde for 10 min. After rinsing, the slides were dehydrated using cold 75%, 95% and 100% ethanol for 5 min each and stored at -20°C for at least one hour. The slices were incubated with 10 μL of Zyto*Dot*SPEC MYC probe following air-drying. Then, the slices were covered with a coverslip, sealed with rubber sealant, denatured for 5 min at 95°C and put in a humidity chamber at 37°C overnight. The next day, the coverslips were removed from the slides, and the slices were washed for 5 min in SSC buffer at 80°C and rinsed with distilled water once. Following blocking with 3% BSA, an anti-digoxigenin antibody (NBP2-31191, Novus) was applied to the slices for 60 min at RT. The slices were further treated with secondary antibody (ENV, Dako) for 30 min following washing with TBS buffer. The signal was visualized using EAL™ DAB+ Chromogen diluted in REAL™ Substrate Buffer (Dako). Hematoxylin was used for nuclear staining. The dehydration and mounting process were performed according to the IHC staining protocol described above.

**In situ hybridization detection of miRNAs**

The IsHyb In Situ Hybridization Kit (BioChain) was used to detect the level of hsa-miR-429 according to the manufacturer’s instructions. Briefly, the FFPE slides were incubated in a 65°C oven for 20 min, deparaffinized in xylene, and rehydrated with gradient ethanol (high to low concentrations from 100 % to 95% to 75%). The slices were fixed with 4% DEPC-paraformaldehyde at RT for 20 min and rinsed with DEPC-PBS twice. The specimens were treated with 10 μg/ml Proteinase K at 37°C for 15 min and washed once with DEPC-PBS. Next, the slices were fixed again with 4% DEPC-paraformaldehyde at RT for 15 min and washed in DEPC-PBS. The samples were then incubated with the prehybridization solution at 50°C for 4 hours and treated with a digoxigenin-labeled RNA oligo probe against hsa-miR-429 (Exiqon) at 55°C for 16 hours. Then, the slides were rinsed with 2X SSC for 10 min at 45°C, with 1.5 X SSC for 10 min at 45°C and then with 0.2 X SSC twice at 37°C for 20 min. The samples were incubated with AP-conjugated anti-digoxigenin antibody diluted in PBS (1:100) for 4 hours at RT following incubation with blocking solution for one hour at RT. The slices were washed with PBS three times and rinsed with 1X alkaline phosphatase buffer twice for 5 min each time. The target RNA was visualized with an optical microscope after incubating with a mixture of nitro-blue tetrazolium (NBT) and 5-bromo-4-chloro-3'-indolyphosphate (BCIP) diluted in 1X alkaline phosphatase buffer in the dark for 18 hours at RT. The Nuclear Fast Red solution (Sigma) was utilized for nuclear counterstaining. The slices were mounted following the dehydration procedure as described above.

**Xenograft animal models**

Thirty-two of four-week-old SCID/beige male mice were purchased from BioLASCO (Taiwan). The animal study was approved by the Institutional Animal Care Committee of Chi-Mei Medical Center (Approval number 109041701). The mice were randomly housed in four individualized ventilating cages (IVCs) with a 12-hour light/dark cycle. After one week of feeding the normal diet (OYDMFG22; BioLASCO, Taiwan), two of these four groups were switched to a high-fat diet regimen (58Y1, TestDiet) for the duration of the study to induce type 2 diabetes mellitus (DM), as described in previous research ^24^. The body weight and fasting blood glucose of mice were robustly increased upon switching to a high-fat diet (Supplementary Figure 1). *Mock*- or *CEBPD*-overexpressing BFTC909 cell lines (1 × 10^7^ cells) resuspended in cold PBS mixed with high-concentration Matrigel (354248, Corning Life Sciences, USA) were injected subcutaneously into 9-week-old mice fed a normal diet or a high-fat diet. The tumor diameters in mice were measured at an indicated time using calipers and calculated by the following equation: V (mm)^3^= (π/6) X width (mm)^2^ X length (mm). The mice were sacrificed with asphyxiation 25 days after transplantation. The tumors were dissected from the mice and soaked in 10% formalin to prepare FFPE blocks for further IHC analysis.

**Analysis of data from the National Health Insurance Research Database (NHIRD)**

We conducted a population-based longitudinal observational cohort study in Taiwan from 2000 to 2010 to analyze whether DM comorbidity impacts the prognosis of UBUC and UTUC by using the NHIRD database. Catastrophic illness registry data were also included in this database, and comorbidity information of patients was collected. The inclusion criteria of the bladder cancer cohort group were as follows: age over 20 years old and UBUC diagnosis (International Classification of Disease [ICD]-9 code 188.9) from 2000 to 2008 or UTUC diagnosis (ICD-9 code 189.1 or 189.2). The exclusion criteria were as follows: age less than 20 years and incomplete demographic data. From both our bladder cancer and UTUC groups, patients with simultaneous DM (ICD-9 code 250.X0 and 250.X2) were identified. After matching age, sex, index year, and all comorbidities, we included 3 times the number of patients without DM to use as a comparison group. The diagnosed comorbidities included chronic kidney disease (CKD) (ICD-9 code 580-589), hypertension (ICD-9 code 401-405), chronic obstructive pulmonary disease (COPD) (ICD-9 code 490-496), stroke (ICD-9 code 430-438), cardiovascular disease (CVD) (ICD-9 code 410-414), peripheral vascular disease (PVD) (ICD-9 code 250.7, 785.4, 443.81, 440-448), hyperlipidemia (ICD-9 code 272.0-272.4), urinary tract disease (ICD-9 code 590-599), and benign prostatic hyperplasia (BPH) (ICD-9 code 600). .

**Statistics**

The correlations and associations between targeted genes or proteins and clinicopathologic variables, as well as comparisons for various functional and animal studies, were evaluated by Spearman's rank correlation coefficient, Chi-square test, or Mann-Whitney U test as appropriate by using SPSS software (version 14.0, IBM, USA). Disease-specific survival (DSS) and metastasis-free survival (MeFS) were estimated and plotted through log-rank tests and Kaplan-Meier curves, respectively. A multivariate Cox proportional hazards model was used to evaluate the independent prognostic impacts of selected parameters. Kaplan-Meier curves were drawn and the cumulative survival rate and log-rank statistic for the NHIRD data were assessed by using SAS version 9.3 (SAS Institute, Cary, NC). Both univariate and multivariate Cox proportional hazards regressions were performed after adjusting for comorbidities to measure the risk of UBUC and UTUC. For all analyses, differences with a two-tailed p-value lower than 0.05 were considered significant.


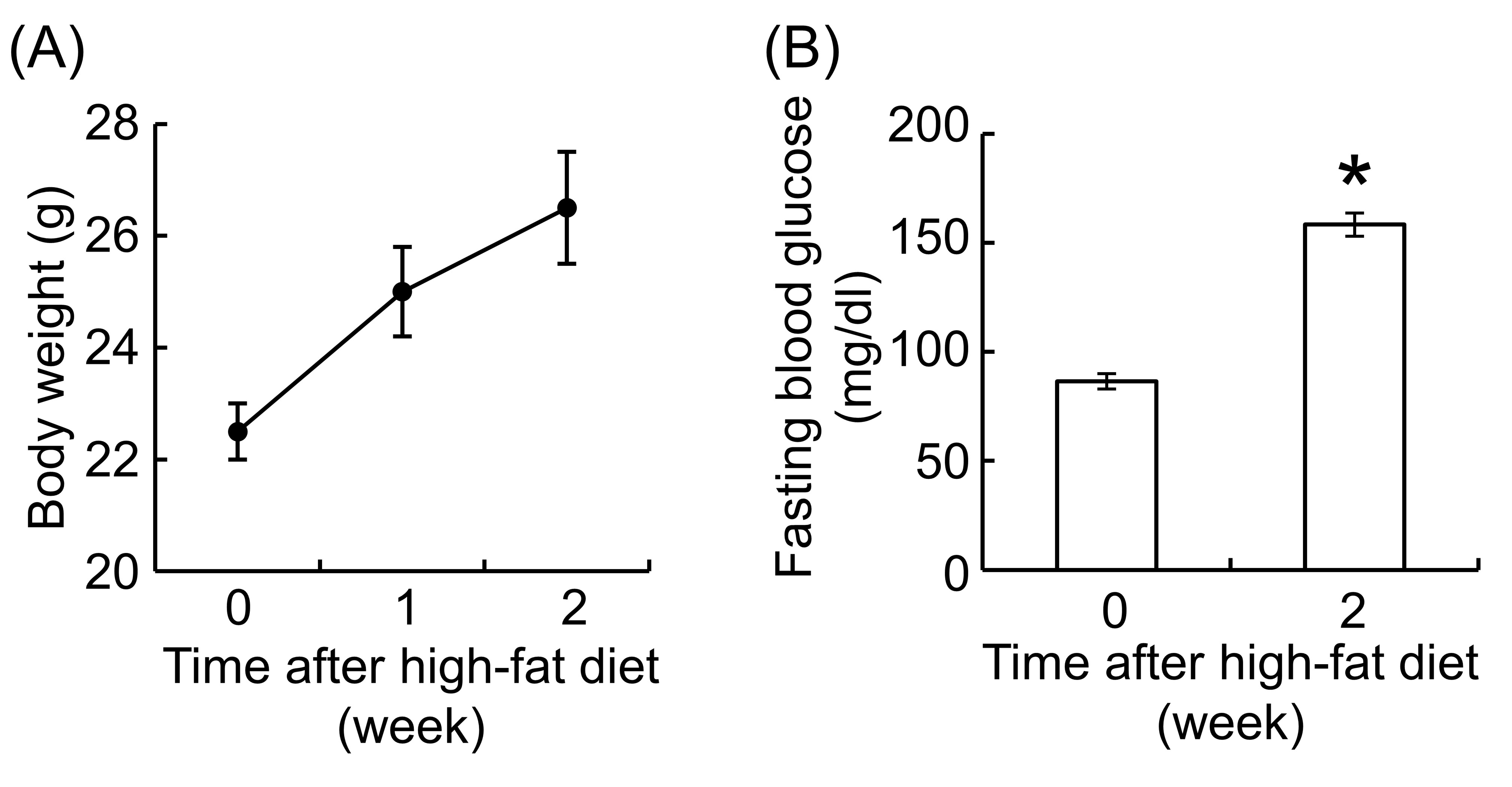


**Supplementary Figure 1. High-fat diet-fed SCID/beige mice exhibit an increase in body weight and fasting blood glucose.**

SCID/beige mice (n=16) were fed a high-fat diet to establish an experimental model of type 2 diabetes mellitus (DM). The body weight (A) and fasting blood glucose levels (B) of mice were measured after feeding with 60% fat for the indicated times. The average body weight of 6-week-old mice was 22.8 g and was increased to 25.7 g and 27.7 g following high fat intake for 7 and 14 days, respectively. A high-fat diet also increased fasting blood glucose levels from 86.5 mg/dl in 6-week-old mice to 158.36 mg/dl in 8-week-old mice. Data were shown as the mean ± SEM. Statistical significance: **P* < 0.0001 (Mann-Whitney U test).


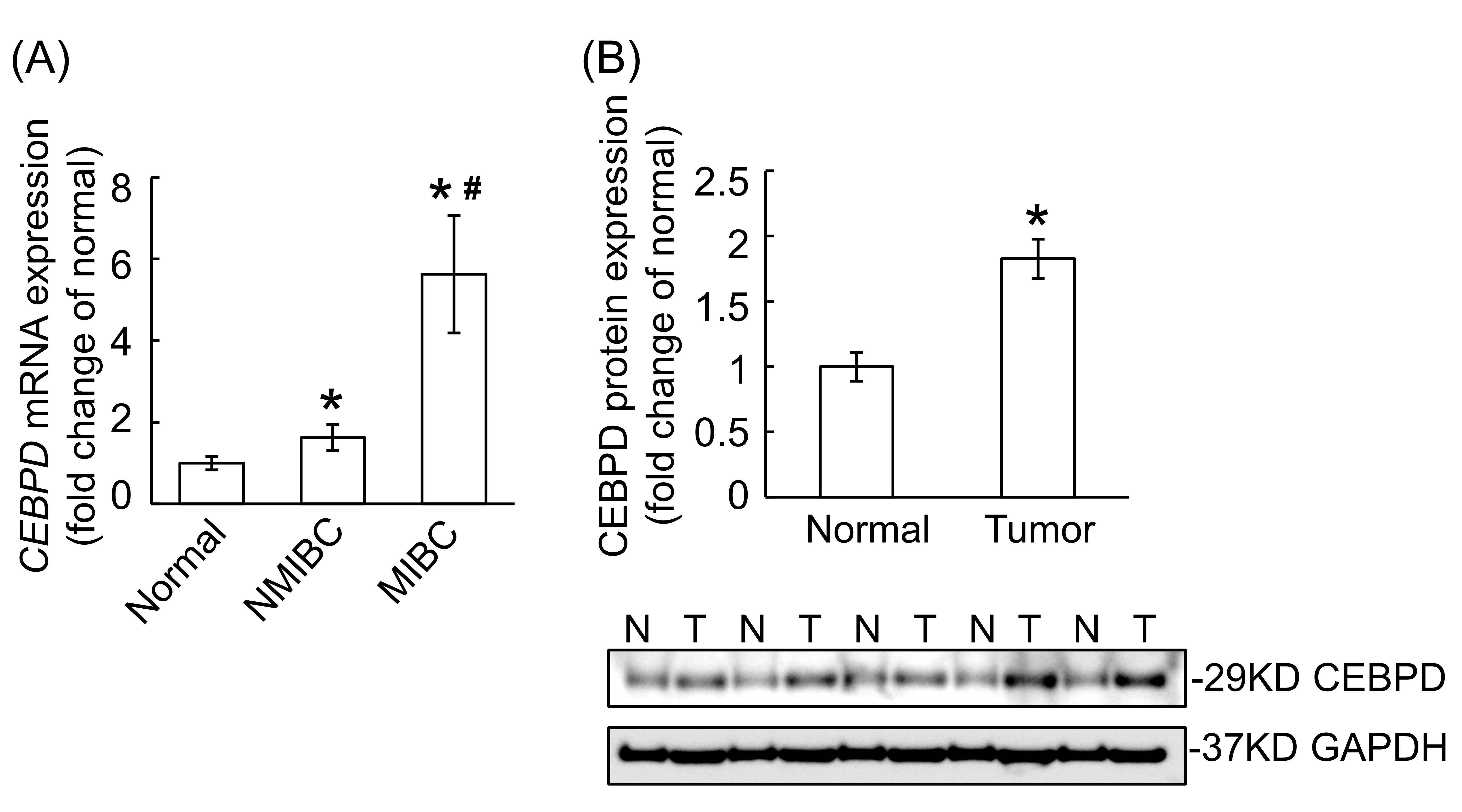


**Supplementary Figure 2. The mRNA and protein level of CEBPD were increased in the UC specimens.**

**(A)** Quantitative RT-PCR indicated a higher *CEBPD* transcript expression in UC than non-tumor urothelium. Intriguingly, the mRNA level of *CEBPD* showed a stepwise escalation from non-tumor urothelium, non-muscle invasive bladder cancers (NMIBC), to Muscle invasive bladder cancers (MIBC). MIBC (n=9) have the highest *CEBPD* transcript followed by NMIBC (n=23) and non-tumor urothelium (Normal, n=32). (NMIBC vs Normal, **p*<0.001; MIBC vs Normal, **p*=0.048; MIBC vs NMIBC, #*p*=0.006;) (B) Immunoblotting showed that UC samples (Tumor, T, n=32) present a higher CEBPD protein level compared to the non-tumor urothelium (Normal, N, n=32). Data were shown as the mean ± SEM. Statistical significance: *^#^*P* < 0.05 (Mann-Whitney U test).


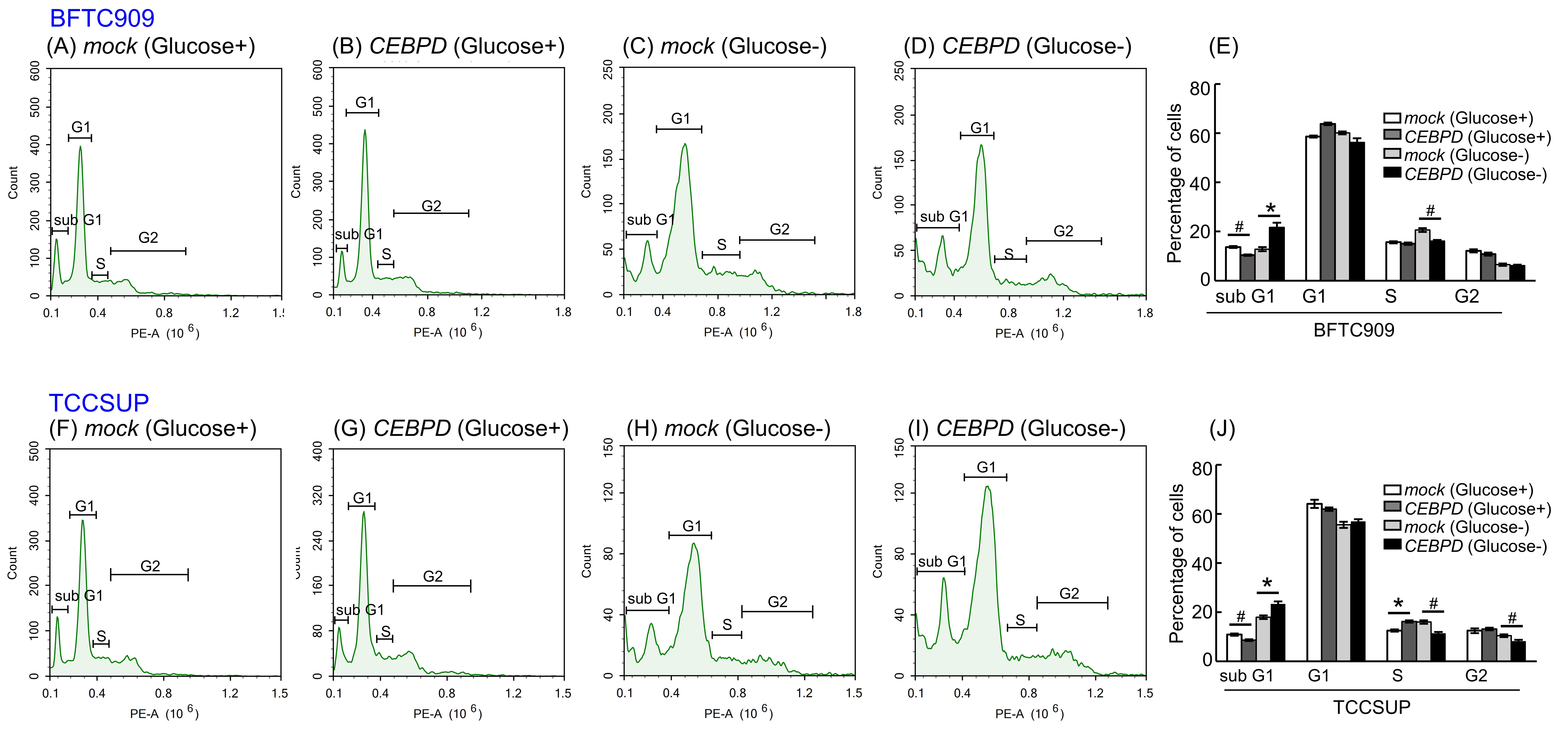


**Supplementary Figure 3. Glucose deprivation leads to adverse apoptosis in CEBPD-overexpressing BFTC909 and TCCSUP.**

*Mock*-expressing and *CEBPD*-overexpressing BFTC909 and TCCSUP were treated with glucose-containing or glucose-free DMEM for 72 hours and applied to the cell cycle analysis. (A, B, F, G) The number of cells at sub G1 phase was declined in the CEBPD-overexpressing BFTC909 and TCCSUP compared with those of mock groups in a glucose-containing medium. (C, D, H, I) Reversely, glucose starvation markedly caused accumulated cells at sub G1 after *CEBPD* overexpression in BFTC909 and TCCSUP. (E, F) Statistical data were presented as mean ± SEM (n = 3). ^#^*P*, **P* < 0.05.





**Supplementary Figure 4. Glucose starvation significantly causes apoptosis in BFTC909 and TCCSUP with CEBPD overexpression.**

Apoptosis assay was applied to the *mock*- and *CEBPD*-infected BFTC909 and TCCSUP treated with glucose-containing or glucose-free medium for 72 hours, respectively. (A, B, F, G) The number of apoptotic cells was slightly decreased in *CEBPD*-infected cells than those of mock-expressing cells in a glucose-containing medium. (C, D, H, I) Glucose removal dramatically elevated the apoptotic cell numbers in BFTC909 and TCCSUP with CEBPD overexpression. Statistical graphics were presented as mean ± SEM (n = 3). ^#^*P*, **P* < 0.05. APO: apoptosis


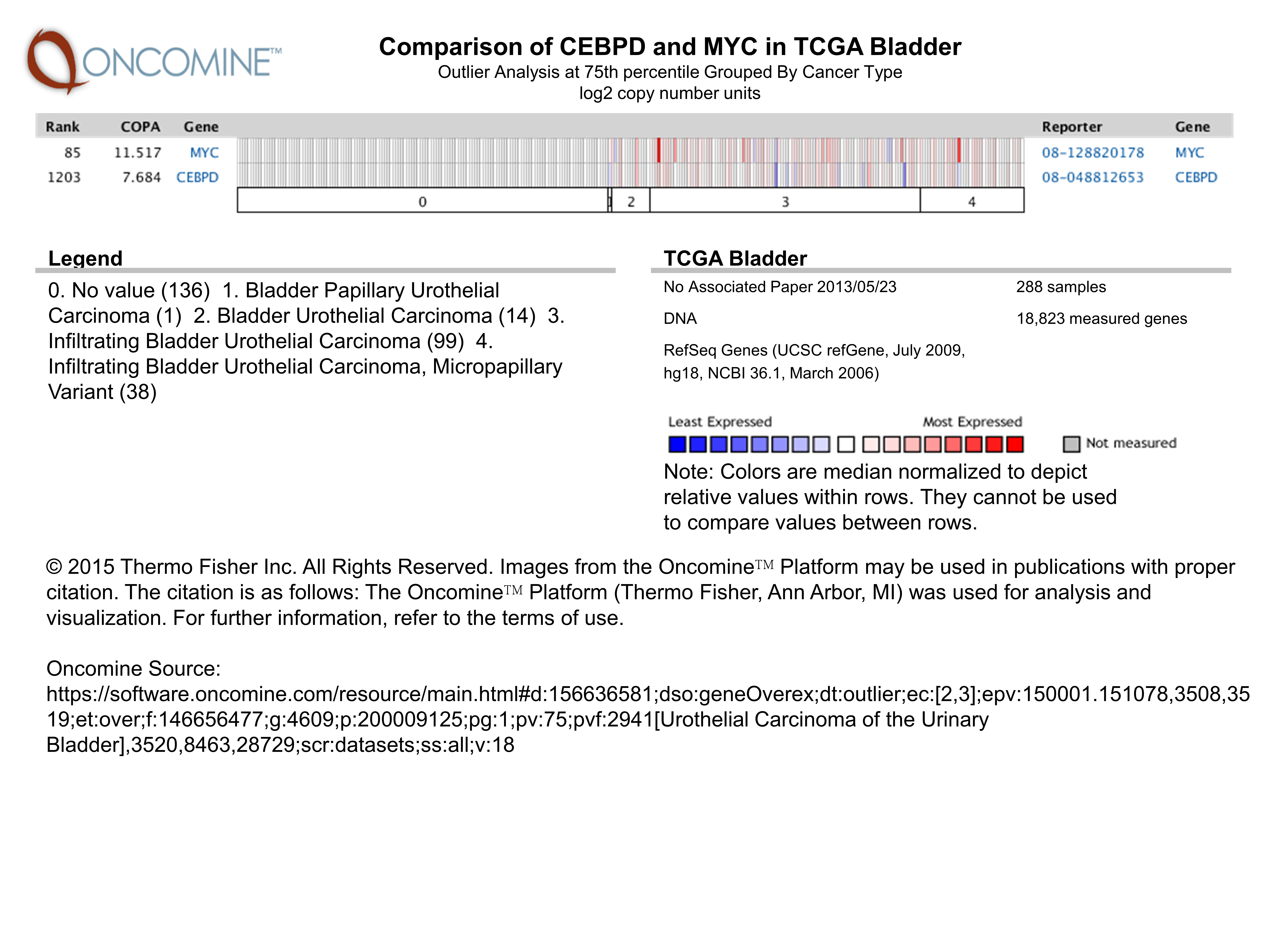


**Supplementary Figure 5. Reappraisal of the TCGA dataset through the Oncomine platform showed a significant positive correlation between the *CEBPD* and *MYC* gene dosages.**


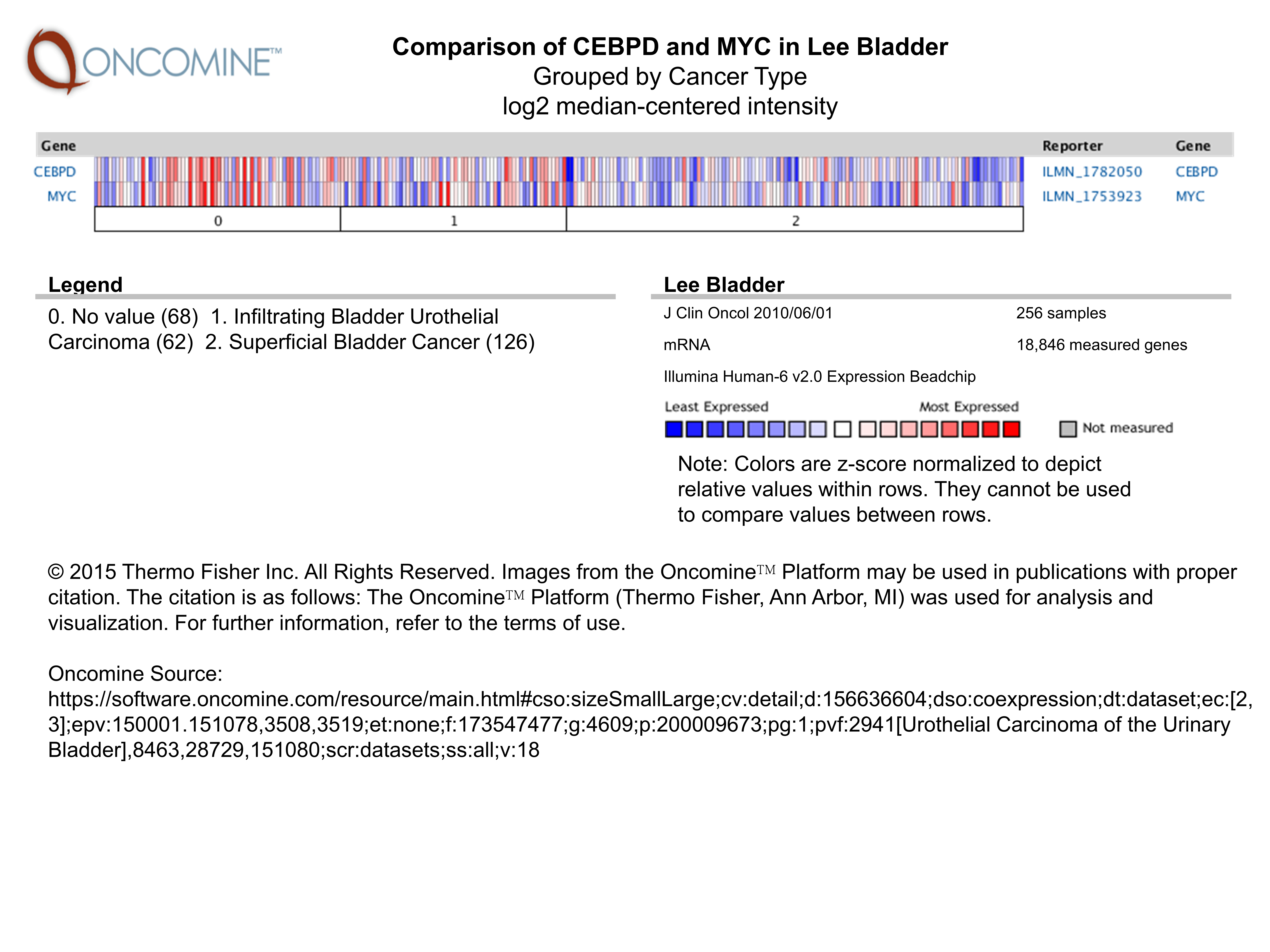


**Supplementary Figure 6. Reappraisal of the TCGA dataset through the Oncomine platform showed a significant positive correlation between the mRNA level of *CEBPD* and *MYC*.**


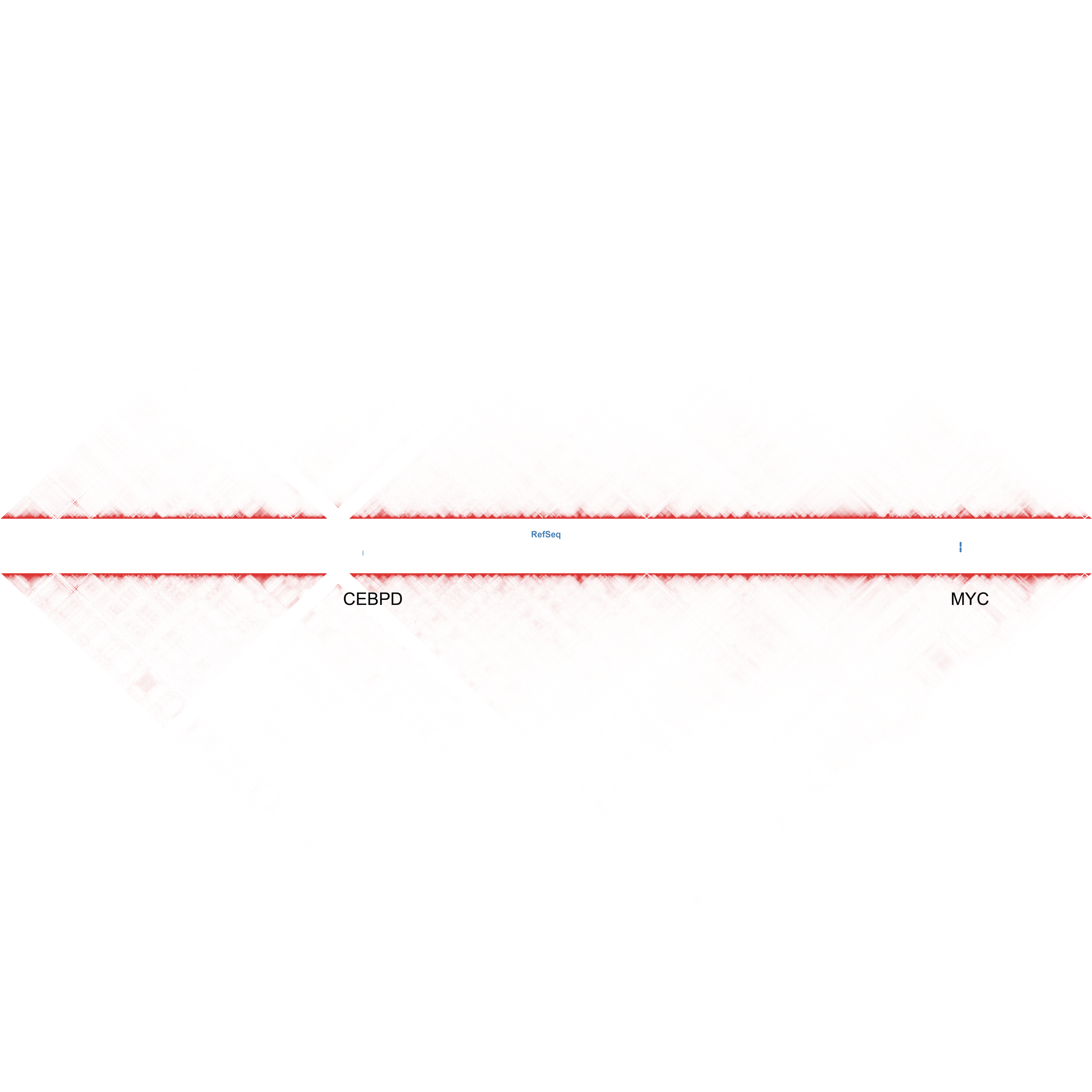


TCCSUP Cell

BFTC909 Cell

**Supplementary Figure 7. Contact frequency map of intra-TAD loops calculated in TCCSUP and BFTC909 cells are illustrated for the high-throughput chromosome conformation capture data for each cell line.** The same genomic region (chromosome 8) is shown on the upper (TCCSUP) and lower (BFTC909) panels, respectively. The gene loci of *CEBPD* (red line) and *MYC* (blue line) genes are indicated. Based on this analysis, there are not predicted intra-TAD loops between *CEBPD* and *MYC* gene.


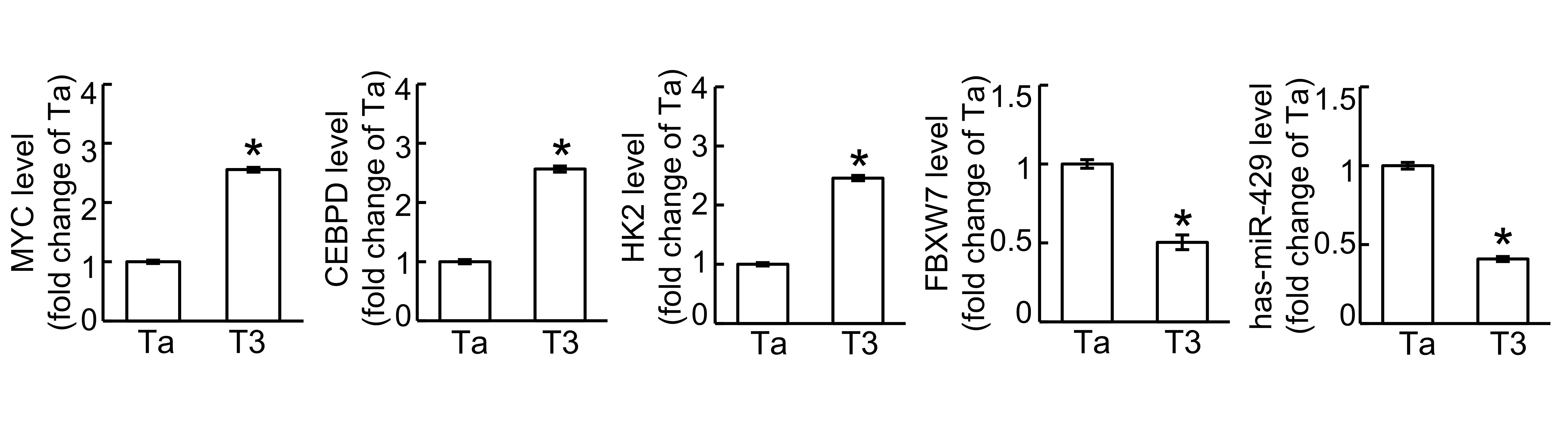


**Supplementary Figure 8.** Quantification of IHC on representative tumor samples shown in figure 5A indicated that the expression of MYC, CEBPD, and HK2 are higher while FBXW7 and has-miR-429 are lower in UBUC samples with higher primary tumor (pT) status. **P* < 0.05


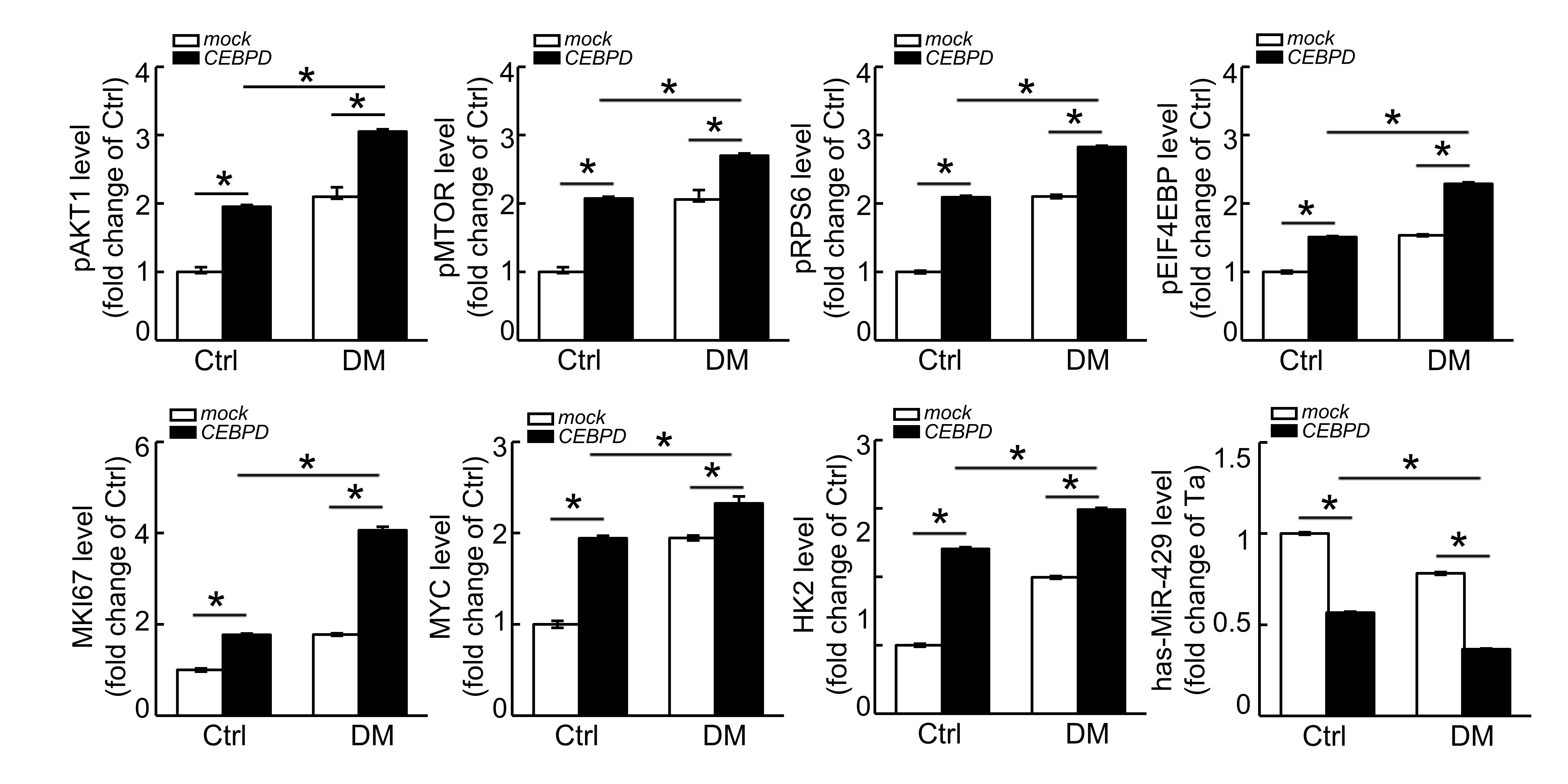


**Supplementary Figure 9.** Quantification of IHC on representative xenografted tumor samples shown in figure 6C indicated that the expression level of pAKT1, pMTOR, pRPS6, pEIF4EBP, MKI67, MYC and HK2 are the highest yet that hsa-miR-429 is strikingly decreased in the xenograft tumors harboring CEBPD overexpression and are implanted onto the mice with high fat diet-induced DM. **P* < 0.05

**Supplementary Table 1. homologous recombination repair-associated genes included in ACTHRD^TM^ panel**

| *ARID1A* | *ATM* | *ATR* | *ATRX* | *BARD1* | *BRCA1* | *BRCA2* | *BRIP1* |
| --- | --- | --- | --- | --- | --- | --- | --- |
| *CDK12* | *CHEK1* | *CHEK2* | *FANCA* | *FANCL* | *FANCM* | *HDAC2* | *NBN* |
| *PALB2* | *PPP2R2A* | *PTEN* | *RAD51* | *RAD51B* | *RAD51C* | *RAD51D* | *RAD54L* |

**Supplementary Table 2. *MYC* and *CEBPD* copy number status in our aCGH cohort comprised of 40 UBUC samples.**

| **Tissue banking no.** | **Sex** | **Age (years)** | **pT status** | **pN status** | **Histological grade** | **Subsequent distal metastasis** | **Status** | ***MYC* gene dosage** | ***CEBPD* gene dosage** |
| --- | --- | --- | --- | --- | --- | --- | --- | --- | --- |
| B1 | M | 83 | T4 | N1 | High | Present | DOD | Gain | Gain |
| B13 | M | 71 | T3 | N0 | High | Present | DUK | Gain | No gain |
| B3 | F | 78 | T2 | N0 | High | Present | AWD | Gain | Gain |
| B16 | M | 91 | T2 | N0 | High | Not identified | NED | No gain | No gain |
| B17 | M | 55 | T2 | N0 | High | Not identified | NED | No gain | No gain |
| B5 | F | 77 | T2 | N0 | High | Not identified | NED | No gain | No gain |
| B11 | F | 55 | T1 | N0 | High | Not identified | NED | No gain | No gain |
| B7 | F | 68 | T1 | N0 | High | Not identified | NED | No gain | No gain |
| CM1757 | F | 65 | T1 | N0 | High | Not identified | NED | No gain | No gain |
| CM1760 | F | 66 | T1 | N0 | High | Not identified | NED | No gain | No gain |
| CM1384 | M | 81 | T1 | N0 | High | Present | DOD | Gain | No gain |
| CM1558 | M | 83 | Ta | N0 | High | Not identified | DUK | No gain | No gain |
| CM1746 | F | 81 | Ta | N0 | Low | Not identified | NED | No gain | No gain |
| CM1748 | M | 72 | Ta | N0 | High | Not identified | NED | Gain | No gain |
| CM1789 | M | 25 | Ta | N0 | Low | Not identified | NED | No gain | No gain |
| CM1354 | M | 65 | T2 | N0 | High | Not identified | NED | No gain | No gain |
| CM1241 | M | 77 | T2 | N0 | High | Not identified | NED | No gain | No gain |
| CM1685 | M | 65 | T1 | N0 | High | Not identified | NED | Gain | No gain |
| 25211427 | M | 85 | T2 | N0 | High | Not identified | DUK | No gain | No gain |
| CM1679 | M | 75 | T4 | N0 | High | Not identified | DUK | No gain | No gain |
| 20510705 | F | 69 | T2 | N0 | High | Present | DOD | Gain | No gain |
| CM1435 | M | 49 | T1 | N0 | High | Not identified | NED | No gain | No gain |
| CM1438 | M | 64 | T3a | N0 | High | Not identified | NED | No gain | No gain |
| CM1476 | F | 71 | T2 | N0 | High | Not identified | DUK | No gain | No gain |
| CM1484 | M | 55 | T2 | N2 | High | Present | AWD | Gain | No gain |
| CM1497 | M | 81 | T3 | N0 | High | Present | DOD | No gain | No gain |
| CM1526 | M | 76 | T3 | N0 | High | Present | DOD | Gain | Gain |
| CM1469 | M | 85 | T3 | N2 | High | Present | DOD | Gain | Gain |
| CM1552 | M | 81 | T3 | N2 | High | Not identified | DUK | No gain | No gain |
| CM1578 | M | 52 | T3 | N0 | High | Present | DOD | No gain | No gain |
| CM1670 | F | 77 | T3 | N0 | High | Present | DOD | No gain | No gain |
| CM1526 | M | 76 | T3 | N0 | High | Present | DOD | Gain | Gain |
| CM1594 | M | 87 | T3 | N0 | High | Not identified | NED | No gain | No gain |
| CM1653 | M | 73 | T3 | N0 | High | Not identified | NED | No gain | No gain |
| CM1679 | M | 75 | T4 | N0 | High | Present | DOD | No gain | No gain |
| CM1681 | M | 57 | T4 | N1 | High | Present | DOD | Gain | Gain |
| CM1688 | F | 75 | T4 | N0 | High | Not identified | NED | No gain | No gain |
| CM1691 | M | 79 | T4 | N0 | High | Present | DOD | Gain | Gain |
| CM1707 | M | 73 | T4 | N0 | High | Present | DOD | Gain | Gain |
| CM1711 | F | 77 | T4 | N0 | High | Not identified | DUK | No gain | No gain |

aCGH: Array-based Comparative Genomic Hybridization

DOD: dead of disease

DUK: dead of unknown cause

AWD: alive with disease

NED: no evidence of disease

**Supplementary Table 3. Mutation involving homologous recombination repair-associated genes**

| **Cell line** | **Gene symbol** | **Chromosome** | **Exon** | **Variant counts** | **Total depth** | **Variant Allele frequency** | **NM ID** | **cDNA change** | **Amino acid change** | **Consequence** | **Variant classification** |
| --- | --- | --- | --- | --- | --- | --- | --- | --- | --- | --- | --- |
| BFTC909 | *BRIP1* | 17 | 6 | 1481 | 4288 | 34.5% | NM_032043 | c.587A>G | p.N196S | Missense | VUS |

**Supplementary Table 4. Copy number alteration involving homologous recombination repair-associated genes in parental BFTC909 cell**

| **Cell line** | **Gene (Exon/Intron)** | **Chromosome** | **Copy number** |
| --- | --- | --- | --- |
| BFTC909 | *BRCA1* (Exon 1-3) | 11 | 4 |
| BFTC909 | *CDK12* | 17 | 1 |

**Supplementary Table 5. Loss of Heterozygosity Analysis of TCCSUP cell line**

| **Sample** | **Total SNP analyzed** | **No. of homozygous SNP before MYC overexpression** | **No. of heterozygous SNP before MYC overexpression** | **# LOH SNP after MYC overexpression** | **% SNP after MYC overexpression** |
| --- | --- | --- | --- | --- | --- |
| TCCSUP | 8833 | 6232 | 2592 | 233 | 9.0% |
| BFTC909 | 8833 | 5745 | 3067 | 538 | 17.5% |

**Supplementary Table 6. Top 7 miRNAs most significantly altered by CEBPD in both BFTC909 and TCCSUP cells as determined by miRNA-targeted RNA sequencing.**

|  | Fold change (log2 ratio) of CEBPD overexpression vs. mock | | Mean fold change (log2 ratio) | miRNA targets* | |
| --- | --- | --- | --- | --- | --- |
|  | BFTC909 | TCCSUP |  | Validated targets | Predicted targets |
| hsa-miR-429 | -1.98540628042077 | -0.439976032358798 | -1.212691156 | WASF3\|XIAP\|MYC\|ZEB1\|OSTF1\|BCL2\|ZFPM2\|SOX2\|RERE\|ZEB2 | ERMP1\|ATP2B1\|CYP1B1\|SCD\|MRAS\|LPAR1  \|AFF1\|TMTC1\|CHST11\|LFNG\|SDC2\|FBXO32  \|FBN2\|TNFSF9\|EGR1\|CD70\|ZNF281\|VEGFA  \|TREM1\|CEBPD\|TGFBR1\|EIF5A2\|OGFRL1  \|OMA1\|TIMP2\|REXO1\|HK2\|CBX5\|KLF4\|IFNGR2\|ZBTB20\|JUN\|OSMR\|ID2 |
| hsa-miR-200b-3p | -1.97431791983769 | -0.376314167256636 | -1.175316044 | CCNE2\|WASF3\|KDR\|XIAP\|ETS1\|ZEB1\|BCL2\|CREB1\|E2F3\|PTPN12\|ZFPM2\|VEGFA\|MATR3\|RERE\|SMAD2\|RND3\|FLT1\|ZEB2 | CYP1B1\|LPAR1\|AFF1\|TMTC1\|CHST11\|LFNG\|CD70\|LURAP1L\|ZNF281\|EIF5A2\|HK2\|KLF4\|ID2\|ERMP1\|SCD\|MRAS\|EFEMP1\|ARRDC3\|FBN2\|EGR1\|TREM1\|TGFBR1\|OGFRL1\|TIMP2\|CBX5\|IFNGR2\|ZBTB20\|JUN\|OSMR |
| hsa-miR-200a-3p | -1.91136241160369 | -0.303114184513026 | -1.107238298 | CCNE2\|WASF3\|MAPK14\|CTNNB1\|ZEB1\|TRAPPC2P1\|GDAP1\|YAP1\|ZFPM2\|SMAD3\|SMAD2\|KEAP1\|DLX5\|ZEB2 | SRD5A1\|CPA4\|AFF1\|TMTC1\|SLC38A1\|PXK\|SDC2\|FAM111A\|ZNF281\|B4GALT1\|THBS1\|IL1RAP\|JADE2\|BAIAP2L1\|PECAM1\|ERMP1\|SCD\|MRAS\|NOX5\|DMWD\|C16orf74\|FBXO32\|OLFML3\|PLXND1\|SLC12A8\|TGFBR1\|RXRA\|TMEM181\|SCARB1\|CBX5\|ST5\|LDLR\|ZBTB20\|JUN\|ZNF512B |
| hsa-miR-125b-5p | -1.742689470514 | -0.391063884995372 | -1.066876678 | CYP24A1\|HMGA2\|BMF\|LIN28B\|TBC1D1\|RAF1\|RPS6KA1\|TP53\|NES\|KLF13\|ERBB2\|NTRK3\|MAPK14\|ETS1\|PIGF\|SGPL1\|IRF4\|SMO\|BCL2\|BCL3\|ERBB3\|MMP13\|GRIN2A\|DGAT1\|NKIRAS2\|GLI1\|BAK1\|AKT1\|TNFAIP3\|LIN28A\|E2F3\|PRDM1\|CBFB\|VDR\|MUC1\|ABTB1\|ARID3B\|FGFR2\|HK2\|BBC3\|HMGA1\|E2F2\|IGF2\|CDKN2A\|BMPR1B | ERMP1\|SCD\|PDXK\|TRIB1\|AFF1\|IER2\|SLC38A1\|TRAF3IP2\|SAA2SAA4\|LFNG\|PXK\|PPME1\|C16orf74\|P2RX4\|TSPAN15\|CD70\|SAA1\|DMPK\|VEGFA\|B4GALT1\|DUSP5\|MLLT1\|ARHGAP40\|SREBF2\|MILR1\|EIF5A2\|RXRA\|RPS6KA1\|SAA4\|HK2\|SCARB1\|PLXNB2\|ST5\|KLF4\|JADE2\|DHCR7\|BAIAP2L1\|ECE1\|ZNF512B\|PECAM1 |
| hsa-miR-200a-5p | -1.64673292712704 | -0.786910654326486 | -1.216821791 |  | SLC1A3\|SLC38A1\|TRAF3IP2\|PXK\|FAM111A\|VEGFA\|THBS1\|EIF5A2\|HK2\|HAUS6\|FAM89A\|PECAM1\|FZD1\|ERMP1\|MRAS\|NAAA\|FBXO32\|CNOT8\|TGFBR1\|TIMP2\|CBX5\|APOBEC3B\|ZBTB20 |
| hsa-miR-138-1-3p | -0.603465924005436 | -1.4100225584554 | -1.006744241 |  | ATP2B1\|SCD\|TENM2\|COLGALT1\|AFF1\|TRAF3IP2\|F3\|AMZ1\|DUSP5\|TFEB\|CDH13\|RXRA\|HAUS6\|ECE1  \|ZBTB20\|OSMR |
| hsa-miR-138-5p | -0.495810768306496 | -1.1773117209991 | -0.836561245 | CASP3\|ARHGEF3\|SUZ12\|RHOC\|BLCAP\|EED\|MXD1\|EZH2\|CDH1\|IGF1R\|CCND3\|SLC45A3\|ROCK2\|SNAI2\|SIRT1\|EID1\|VIM\|RELN\|PTK2\|SOX4\|HIF1A\|TERT\|H2AFX | EPAS1\|SLC2A1\|SLC38A1\|CHST11\|TRAF3IP2\|LFNG\|PXK\|LTBP4\|ADM\|TGM2\|VEGFA\|THBS1\|SREBF2\|COL12A1\|EIF5A2\|OMA1\|TOM1\|ID3\|PECAM1\|SCD\|MRAS\|TENM2\|COLGALT1\|PDXK\|NINJ1\|NOX5\|DMWD\|ABCF2\|ARRDC3\|FBXO32\|FBN2\|CNOT8\|TREM1\|EPHB2\|RXRA\|RPS6KA1\|CRAT\|TMEM181\|PLXNB2\|CPSF1\|CDA\|CBX5\|LDLR\|APOBEC3B\|ECE1\|ZBTB20\|PDPN |

Validated targets and predicted targets based on the Miranda database (http://cbio.mskcc.org/miRNA2003/miranda.html)

**Supplementary Table 7. Demographic characteristics and comparison of the incidence rate of comorbidities between non-DM and DM cohorts in UBUC or UTUC from NHIRD database.**

|  | Urinary bladder urothelial carcinoma (n=8436) | | | | | Upper urinary tract urothelial carcinoma (n=3232) | | | | |
| --- | --- | --- | --- | --- | --- | --- | --- | --- | --- | --- |
|  | Non-DM (n=6327) | | DM (n=2109) | |  | Non-DM (n=2424) | | DM (n=808) | |  |
|  | No. of events | (%) | No. of events | (%) | p-value | No. of events | (%) | No. of events | (%) | p-value |
| Age, mean ±SD | 70.59 | (10.49) | 70.51 | (10.43) |  | 69.91 | (9.59) | 69.78 | (9.63) |  |
| <60 | 1046 | (16.5) | 345 | (16.4) | 0.852 | 342 | (14.1) | 122 | (15.1) | 0.487 |
| ≥60 | 5281 | (83.5) | 1764 | (83.6) |  | 2082 | (85.9) | 686 | (84.9) |  |
| Sex |  |  |  |  |  |  |  |  |  |  |
| Female | 1996 | (31.5) | 669 | (31.7) | 0.882 | 1533 | (63.2) | 508 | (62.9) | 0.850 |
| Male | 4331 | (68.5) | 1440 | (68.3) |  | 891 | (36.8) | 300 | (37.1) |  |
| Comorbidities |  |  |  |  |  |  |  |  |  |  |
| Chronic kidney disease | 2100 | (33.2) | 717 | (34.0) | 0.497 | 1051 | (43.4) | 342 | (42.3) | 0.608 |
| Hypertension | 4750 | (75.1) | 1574 | (74.6) | 0.685 | 1989 | (82.1) | 656 | (81.2) | 0.580 |
| COPD | 3068 | (48.5) | 1047 | (49.6) | 0.359 | 1140 | (47.0) | 381 | (47.2) | 0.951 |
| Stroke | 744 | (11.8) | 275 | (13.0) | 0.118 | 218 | (9.0) | 80 | (9.9) | 0.440 |
| Ischemic heart disease | 392 | (6.2) | 152 | (7.2) | 0.101 | 117 | (4.8) | 41 | (5.1) | 0.777 |
| PVD | 135 | (2.1) | 46 | (2.2) | 0.896 | 40 | (1.7) | 13 | (1.6) | 0.936 |
| Hyperlipidemia | 1945 | (30.7) | 656 | (31.1) | 0.754 | 1299 | (53.6) | 442 | (54.7) | 0.582 |
| Benign prostatic hyperplasia | 3309 | (52.3) | 1095 | (51.9) | 0.763 | 633 | (26.1) | 202 | (25.0) | 0.531 |
| Urinary tract disease | 4906 | (77.5) | 1627 | (77.1) | 0.707 | 2022 | (83.4) | 660 | (81.7) | 0.256 |

**Supplementary Table 8. Relative mortality risk between non-DM and DM cohorts at different follow-up times in UBUC or UTUC from NHIRD database.**

|  | Urinary bladder urothelial carcinoma (n=8436) | | | | | | | Upper urinary tract urothelial carcinoma (n=3232) | | | | | | |
| --- | --- | --- | --- | --- | --- | --- | --- | --- | --- | --- | --- | --- | --- | --- |
|  | Non-DM  (n=6327) | | DM  (n=2109) | | Non-DM vs. DM | | | Non-DM  (n=2424) | | DM  (n=808) | | Non-DM vs. DM | | |
|  | No. of deaths | (%) | No. of deaths | (%) | Adjusted HR | (95% CI) | p-value | No. of deaths | (%) | No. of deaths | (%) | Adjusted HR | (95% CI) | p-value |
| 3-year  follow-up | 2480 | (39.2) | 946 | (44.9) | 1.17 | (1.08-1.26) | <0.001* | 865 | (35.7) | 310 | (38.4) | 1.06 | (0.93-1.20) | 0.410 |
| 5-year  follow-up | 3040 | (48.0) | 1182 | (56.0) | 1.22 | (1.14-1.30) | <0.001* | 1097 | (45.3) | 413 | (51.1) | 1.12 | (1.01-1.26) | 0.044* |
| 10-year  follow-up | 3783 | (59.8) | 1479 | (70.1) | 1.29 | (1.22-1.37) | <0.001* | 1365 | (56.3) | 518 | (64.1) | 1.17 | (1.06-1.29) | 0.003* |

Adjusted for age, sex, and all comorbidities. *Statistical significance

**Supplementary Table 9**. **Comparison of demographic characteristics and comorbidities on mortality rate between non-DM and DM cohorts in UBUC or UTUC from NHIRD database**

| Urinary bladder urothelial carcinoma (n=8436) | | | | | | | | | | | | Upper urinary tract urothelial carcinoma (n=3232) | | | | | | | | |
| --- | --- | --- | --- | --- | --- | --- | --- | --- | --- | --- | --- | --- | --- | --- | --- | --- | --- | --- | --- | --- |
|  | Non-DM (n=6327) | | DM  (n=2109) | | | | Non-DM v.s. DM | | | | p-value of interaction | Non DM  (n=2424) | | DM  (n=808) | | | Non-DM v.s. DM | | | p-value of interaction |
|  | No. of deaths | (%) | No. of deaths | (%) | | | Adjusted HR  (95% CI) | | | p-value |  | No. of deaths | (%) | No. of deaths | (%) | | Adjusted HR  (95% CI) | | p-value |  |
| Age |  |  |  |  | | |  | |  |  |  |  |  |  |  | |  |  |  |  |
| <60 | 180 | (20.8) | 165 | (31.5) | | | 1.59 | | (1.32-1.91) | <0.001* | 0.053 | 67 | (22.4) | 55 | (33.3) | | 1.45 | (1.03-2.03) | 0.032* | 0.055 |
| ≥60 | 450 | (19.5) | 1314 | (27.7) | | | 1.26 | | (1.19-1.35) | <0.001* |  | 223 | (21.2) | 463 | (26.9) | | 1.13 | (1.02-1.26) | 0.021* |  |
| Sex |  |  |  |  | | |  | |  |  |  |  |  |  |  | |  |  |  |  |
| Female | 194 | (19.6) | 475 | (28.4) | | | 1.26 | | (1.13-1.41) | <0.001* | 0.729 | 181 | (20.5) | 327 | (28.3) | | 1.23 | (1.08-1.40) | 0.002* | 0.322 |
| Male | 436 | (20.0) | 1004 | (28.0) | | | 1.31 | | (1.22-1.41) | <0.001* |  | 109 | (23.5) | 191 | (26.3) | | 2.69 | (2.12-3.42) | <0.001* |  |
| Comorbidities |  |  |  |  | | |  | |  |  |  |  |  |  |  | |  |  |  |  |
| Chronic kidney disease |  |  |  |  | | |  | |  |  |  |  |  |  |  | |  |  |  |  |
| No | 464 | (20.0) | 928 | (28.1) | | | 1.32 | | (1.22-1.42) | <0.001* | 0.321 | 184 | (22.2) | 282 | (27.9) | | 1.23 | (1.07-1.41) | 0.004* | 0.323 |
| Yes | 166 | (19.4) | 551 | (28.1) | | | 1.24 | | (1.12-1.37) | <0.001* |  | 106 | (20.3) | 236 | (27.1) | | 1.13 | (0.97-1.31) | 0.118 |  |
| Hypertension |  |  |  |  | |  | |  | |  |  |  |  |  |  | |  |  |  |  |
| No | 212 | (21.1) | 323 | (29.2) | | 1.35 | | (1.18-1.53) | | <0.001* | 0.432 | 66 | (23.6) | 86 | (28.0) | | 1.21 | (0.94-1.56) | 0.134 | 0.929 |
| Yes | 418 | (19.3) | 1156 | (27.8) | | 1.28 | | (1.19-1.37) | | <0.001* |  | 224 | (21.0) | 432 | (27.4) | | 1.18 | (1.05-1.31) | 0.004* |  |
| COPD |  |  |  |  | |  | |  | |  |  |  |  |  |  | |  |  |  |  |
| No | 337 | (18.7) | 725 | (28.8) | | 1.41 | | (1.29-1.53) | | <0.001* | 0.005* | 151 | (20.3) | 276 | (28.5) | | 1.32 | (1.15-1.52) | <0.001* | 0.051 |
| Yes | 293 | (21.4) | 754 | (27.5) | | 1.19 | | (1.10-1.30) | | <0.001* |  | 139 | (22.9) | 242 | (26.4) | | 1.06 | (0.91-1.22) | 0.471 |  |
| Stroke |  |  |  |  | |  | |  | |  |  |  |  |  |  | |  |  |  |  |
| No | 592 | (20.0) | 1242 | (27.9) | | 1.30 | | (1.22-1.39) | | <0.001* | 0.543 | 276 | (21.5) | 452 | (27.3) | | 1.19 | (1.07-1.33) | 0.001* | 0.514 |
| Yes | 38 | (17.8) | 237 | (29.4) | | 1.24 | | (1.06-1.45) | | <0.001* |  | 14 | (20.6) | 66 | (28.7) | | 1.09 | (0.81-1.45) | 0.577 |  |
| Ischemic heart disease |  |  |  | |  | |  |  | |  |  |  |  |  | |  |  |  |  |  |
| No | 612 | (19.9) | 1345 | | (27.9) | | 1.31 | (1.23-1.39) | | <0.001* | 0.543 | 281 | (21.4) | 486 | | (27.6) | 1.20 | (1.08-1.33) | 0.001* | 0.231 |
| Yes | 18 | (18.2) | 134 | | (30.1) | | 1.20 | (0.97-1.47) | | 0.089 |  | 9 | (27.3) | 32 | | (25.6) | 1.04 | (0.69-1.58) | 0.850 |  |
| PVD |  |  |  | |  | |  |  | |  |  |  |  |  | |  |  |  |  |  |
| No | 621 | (19.9) | 1442 | | (28.1) | | 1.29 | (1.21-1.37) | | <0.001* | 0.883 | 287 | (21.5) | 508 | | (27.5) | 1.18 | (1.06-1.30) | 0.002* | 0.669 |
| Yes | 9 | (18.0) | 37 | | (28.2) | | 1.45 | (0.97-2.15) | | 0.069 |  | 3 | (21.4) | 10 | | (25.6) | 1.94 | (0.84-4.50) | 0.123 |  |
| Hyperlipidemia |  |  |  | |  | |  |  | |  |  |  |  |  | |  |  |  |  |  |
| No | 364 | (18.6) | 1089 | | (38.1) | | 1.30 | (1.22-1.40) | | <0.001* | 0.688 | 122 | (21.6) | 244 | | (26.3) | 1.09 | (0.94-1.26) | 0.251 | 0.131 |
| Yes | 266 | (21.9) | 390 | | (28.1) | | 1.28 | (1.14-1.44) | | <0.001* |  | 168 | (21.4) | 274 | | (28.6) | 1.28 | (1.11-1.48) | <0.001* |  |
| Benign prostatic hyperplasia |  |  |  | |  | |  |  | |  |  |  |  |  | |  |  |  |  |  |
| No | 329 | (20.6) | 685 | | (28.1) | | 1.27 | (1.16-1.38) | | <0.001* | 0.651 | 221 | (21.5) | 385 | | (28.2) | 1.19 | (1.06-1.33) | 0.004* | 0.921 |
| Yes | 301 | (19.1) | 794 | | (28.1) | | 1.32 | (1.21-1.43) | | <0.001* |  | 69 | (21.6) | 133 | | (25.8) | 1.18 | (0.97-1.44) | 0.101 |  |
| Urinary tract disease |  |  |  | |  | |  |  | |  |  |  |  |  | |  |  |  |  |  |
| No | 163 | (19.9) | 319 | | (29.4) | | 1.41 | (1.23-1.60) | | <0.001* | 0.125 | 51 | (22.5) | 97 | | (30.0) | 1.25 | (0.98-1.59) | 0.069 | 0.707 |
| Yes | 467 | (19.8) | 1160 | | (27.8) | | 1.26 | (1.18-1.35) | | <0.001* |  | 239 | (21.3) | 421 | | (27.0) | 1.17 | (1.04-1.31) | 0.007* |  |

Adjusted for age, sex, and other comorbidities. *Statistical significance
